# Real-life Evaluation of an Interactive Versus Noninteractive e-Learning Module on Chronic Obstructive Pulmonary Disease for Medical Licentiate Students in Zambia: Web-Based, Mixed Methods Randomized Controlled Trial

**DOI:** 10.2196/34751

**Published:** 2022-02-24

**Authors:** Elena Schnieders, Freda Röhr, Misho Mbewe, Aubrey Shanzi, Astrid Berner-Rodoreda, Sandra Barteit, Valérie R Louis, Petros Andreadis, Gardner Syakantu, Florian Neuhann

**Affiliations:** 1 Heidelberg Institute of Global Health (HIGH), Faculty of Medicine and University Hospital, Heidelberg University Heidelberg Germany; 2 School of Medicine and Clinical Sciences, Levy Mwanawasa Medical University Lusaka Zambia; 3 SolidarMed Lusaka Zambia

**Keywords:** distance education, randomized controlled trial, personal satisfaction, knowledge, user-centered design, chronic obstructive pulmonary disease, interactive, noninteractive, low- and middle-income country, LMIC, mobile phone

## Abstract

**Background:**

e-Learning for health professionals in many low- and middle-income countries (LMICs) is still in its infancy, but with the advent of COVID-19, a significant expansion of digital learning has occurred. Asynchronous e-learning can be grouped into interactive (user-influenceable content) and noninteractive (static material) e-learning. Studies conducted in high-income countries suggest that interactive e-learning is more effective than noninteractive e-learning in increasing learner satisfaction and knowledge; however, there is a gap in our understanding of whether this also holds true in LMICs.

**Objective:**

This study aims to validate the hypothesis above in a resource-constrained and real-life setting to understand e-learning quality and delivery by comparing interactive and noninteractive e-learning user satisfaction, usability, and knowledge gain in a new medical university in Zambia.

**Methods:**

We conducted a web-based, mixed methods randomized controlled trial at the Levy Mwanawasa Medical University (LMMU) in Lusaka, Zambia, between April and July 2021. We recruited medical licentiate students (second, third, and fourth study years) via email. Participants were randomized to undergo asynchronous e-learning with an interactive or noninteractive module for chronic obstructive pulmonary disease and informally blinded to their group allocation. The interactive module included interactive interfaces, quizzes, and a virtual patient, whereas the noninteractive module consisted of PowerPoint slides. Both modules covered the same content scope. The primary outcome was learner satisfaction. The secondary outcomes were usability, short- and long-term knowledge gain, and barriers to e-learning. The mixed methods study followed an explanatory sequential design in which rating conferences delivered further insights into quantitative findings, which were evaluated through web-based questionnaires.

**Results:**

Initially, 94 participants were enrolled in the study, of whom 41 (44%; 18 intervention participants and 23 control participants) remained in the study and were analyzed. There were no significant differences in satisfaction (intervention: median 33.5, first quartile 31.3, second quartile 35; control: median 33, first quartile 30, second quartile 37.5; *P*=.66), usability, or knowledge gain between the intervention and control groups. Challenges in accessing both e-learning modules led to many dropouts. Qualitative data suggested that the content of the interactive module was more challenging to access because of technical difficulties and individual factors (eg, limited experience with interactive e-learning).

**Conclusions:**

We did not observe an increase in user satisfaction with interactive e-learning. However, this finding may not be generalizable to other low-resource settings because the post hoc power was low, and the e-learning system at LMMU has not yet reached its full potential. Consequently, technical and individual barriers to accessing e-learning may have affected the results, mainly because the interactive module was considered more difficult to access and use. Nevertheless, qualitative data showed high motivation and interest in e-learning. Future studies should minimize technical barriers to e-learning to further evaluate interactive e-learning in LMICs.

## Introduction

### Background

Medical education in sub-Saharan Africa (SSA) has expanded significantly in the last 3 decades as countries in the region have tried to address the critical shortfall of key health workers [1]. However, several factors threaten to impede developments on this front. These include a lack of teaching infrastructure and adequately trained medical teaching staff and the challenges many health professionals face as they attempt to manage heavy teaching workloads alongside priorities in clinical practice [1]. Another factor that affects advances in training clinicians is *brain-drain*—health professionals with critical teaching skills and experience relocate to high-income countries (HICs) in pursuit of better remuneration and employment conditions [2]. Although these systemic challenges threaten to impede medical education, there is a critical need to find ways to improve the educational and teaching experiences of students and lecturers in low-income settings, in which e-learning has been explored as a catalyst [3].

e-Learning is considered as potent as traditional classroom learning alone in a low-resource context [4], with several benefits. For instance, materials can be accessed at any time and in any geographic location using an internet connection, content may be available for offline access after download, and materials can be studied at the student’s own pace [5,6]. Furthermore, e-learning access is scalable, thus facilitating teaching large numbers of students, and updating the content is also more efficient [6]. e-Learning is considered potentially cost-effective owing to reduced costs of instruction, travel, and classroom infrastructure [5-7]. However, the initial implementation of e-learning and its running costs are expensive, which can be a challenge, especially in low- and middle-income countries (LMICs) [4,5,8]. Often, e-learning in LMICs does not progress past the pilot stage because the e-learning approach is not adapted to the individual needs of the institution and is frequently not implemented sustainably—a phenomenon coined *pilot-itis* [8].

As with traditional classroom learning, e-learning is a heterogeneous learning method, which means there are different ways of learning on the web. An aspect is the difference between interactive and noninteractive e-learning. Interactive e-learning is defined as content that reacts to a learner’s actions [9]. Examples of interactive e-learning include quizzes, interactive interfaces, virtual patients, and serious games. Virtual patients often involve learners in interactive clinical scenarios with a virtual person to teach clinical reasoning skills [10]. Serious games are technology-based games to teach a certain skill, mindset, or provide information [11]. Noninteractive e-learning, on the other hand, is defined as learning through static, nonresponsive web-based resources, such as PowerPoint slides without interactive elements, PDF scripts, or videos [3,12].

In health education research, interactive e-learning is often deemed more effective than noninteractive e-learning. Several studies in HICs have shown a positive effect of interactive e-learning on user satisfaction or knowledge compared with noninteractive e-learning [13-19]. In addition, knowledge frequently increases when user satisfaction is high [13,14,18]. However, studies comparing an interactive e-learning method with a noninteractive e-learning method for health care personnel in LMICs are rare, which potentially makes assumptions about the effectiveness of interactive e-learning in LMICs difficult for lecturers and other stakeholders. A study conducted in Colombia, an upper-middle–income country, compared learning on the commonly used e-learning platform Moodle with learning using an interactive intelligent tutor system. The latter fared better in their evaluation of medical students’ knowledge, learning efficiency, and usability [20].

An e-learning system for medical licentiate (ML) students was set up in 2016 at the Chainama College of Health Sciences in Lusaka, Zambia, which is now part of the Levy Mwanawasa Medical University (LMMU). In addition, third- and fourth-year students received tablets to facilitate e-learning access [21,22]. The e-learning system was then assessed using a mixed methods format and considered functional in these settings. However, the program faced some challenges, as students’ and lecturers’ use of the e-learning platform was low. Possible explanations were the low quality of the tablets used and insufficient training with the technology. Another shortcoming was the low availability of diverse and multimedia e-learning content, as mainly noninteractive materials were available [21-23].

This study aims to contribute to the multimedia e-learning content at the LMMU by providing targeted e-learning materials on chronic obstructive pulmonary disease (COPD). COPD is a noncommunicable, chronic but preventable disease that occupies the seventh place in the worldwide list of years of life lost [24,25]. Of 196 million people >40 years in SSA, approximately 26 million were estimated to have COPD in 2010, and the literature suggests that >80% of COPD deaths occur in LMICs worldwide [26,27]. To treat COPD, health care workers need to be aware of the disease, its diagnosis and management, and adequate guidelines, such as the international guidelines of the Global Initiative for Chronic Obstructive Lung Disease (GOLD) [25]. However, this is not sufficient, as COPD is mostly underrepresented in medical education in SSA, leading to COPD underdiagnosis [28-32]. Improved COPD education for health care workers in low-resource settings is essential, as smoking and old age—the disease’s key cause and risk factor, respectively—have been increasing in LMICs, predicting growth in COPD cases [25].

### Study Objectives

The overarching objective of this web-based study is to compare learning outcomes from an interactive and noninteractive e-learning module on the topic of COPD for ML students following a mixed methods randomized controlled trial (RCT). The aim was to improve the understanding of real-life e-learning quality and delivery at the LMMU. Subsequently, the primary outcome for this study was user satisfaction, and the secondary outcomes were usability, short- and long-term knowledge gain, and barriers to e-learning access for ML students. These outcomes were determined quantitatively by web-based questionnaires and qualitatively by web-based rating conferences that explored how students experienced e-learning. On the basis of findings from previous studies, we hypothesized that an interactive e-learning module would be more effective in increasing learners’ satisfaction and knowledge gain than a noninteractive module. It should be noted that most previous studies were conducted in HICs and not in a low-income setting.

## Methods

### Overview

This study adheres to the CHERRIES (Checklist for Reporting Results of Internet E-Surveys) checklist and the CONSORT-EHEALTH (Consolidated Standards of Reporting Trials of Electronic and Mobile Health Applications and Online Telehealth) guidelines for reporting eHealth and mobile health RCTs (Multimedia Appendix 1) [33,34]. Qualitative data results are presented according to the COREQ (Consolidated Criteria for Reporting Qualitative Research) checklist [35]. This mixed methods study used an explanatory sequential design in which qualitative findings were used to clarify quantitative results.

### Study Setting and Design

The RCT with an allocation ratio of 1:1 took place in Zambia, a lower-middle–income country. The trial was conducted on the web at the LMMU in Lusaka, Zambia, for 11 weeks between April and July 2021. The LMMU was established in 2018 and has become the largest health training institution in the country and the fourth public university [36]. e-Learning at the Chainama College of Health Sciences, now part of the LMMU, was successfully implemented in 2016/2017. The study design aimed to evaluate interactive and noninteractive e-learning in a real-life setting, meaning no study-related and specific e-learning training was provided [21-23].

### Ethics

The study protocol was approved by the ethics committee of the Heidelberg University and the local ethics committee of the LMMU (Heidelberg S-691/2020; LMMU 00007/20). The trial was not registered in accordance with the International Committee of Medical Journal Editors [37].

### Study Sample

ML students in their second, third, and fourth year of the Bachelor of Clinical Sciences program at the LMMU were invited to participate. It was assumed that existing knowledge on COPD was low and that all students had computer literacy, as the technology experience of ML students was assessed to be moderate in 2017 [23]. As there were only approximately 200 ML students in the second, third, and fourth year in the Bachelor of Clinical Sciences program at the LMMU, instead of a sample size calculation, a convenience sample of all eligible students was chosen. A sample size of approximately 50 participants was deemed feasible, considering consent and attrition rates.

### Study Materials

#### Development and Testing

With the aid of FN, who received training at the center for key competencies in didactics at the Heidelberg University, ES developed both e-learning modules. The modules were then uploaded for asynchronous use on the e-learning platform Moodle. Given the e-learning implementation in 2016/2017, it was assumed that all students had access to the e-learning platform and electronic devices [21-23]. Three study team members (FN, PA, and ES) tested the web-based e-learning material before the trial on different digital devices, such as desktop computers and smartphones. Changes were incorporated before the start of the study, and no further changes were made.

#### Content

Both modules contained key information from the GOLD report 2021, specialist literature, and pulmonological experts [25,38, 39]. The GOLD report is a document published annually that summarizes global information on COPD through the latest scientific literature. Essential knowledge on COPD definition, epidemiology, etiology, symptoms, diagnosis, severity assessment, differential diagnosis, therapy, and prognosis was included in the e-learning modules at the appropriate level according to the curriculum of the ML program. By continuously comparing slides on subtopics and copying and pasting information from one module to the other, it was ensured that both modules comprised the same content scope.

#### Standard Material—Noninteractive

The noninteractive e-learning module on COPD for the control group included an average of 5 bullet points per slide with several figures and tables (see Multimedia Appendix 2 for screenshots of the noninteractive module).

#### Interactive Material

The intervention group was provided access to a voice-over interactive e-learning module designed with iSpring Suite (see Multimedia Appendix 3 for screenshots of the interactive module) [40]. The interactive module was composed of a simple interactive environment that allowed the user to control the representation of information and receive predetermined feedback on activities [9]. In more detail, the interactive module included the following items sorted from representation control to obtaining feedback: interactive interfaces including drag and drop options; interactive X-ray images to be explored with the curser; a puzzle; a 10-step virtual patient, including different question paths representing a typical COPD exacerbation case; and 3 short multiple-choice quizzes, for which participants received feedback. Furthermore, the principles of adult learning by Taylor and Hamdy [41] were incorporated into the module. For example, the learner had to complete certain tasks several times, which challenged existing knowledge on COPD and might have put the learner in a *dissonance phase* as existing knowledge might have been incomplete. This *dissonance phase* was followed by a *refinement phase* in which the learner received information on the problem's solution.

### Outcome Measures

#### Overview

The primary outcome was learner satisfaction based on a comparison of interactive and noninteractive e-learning modules. The secondary outcomes were system usability and short- and long-term knowledge gain. After study initiation, an outcome was added—identified barriers to asynchronous e-learning—as feedback from participants revealed usability issues. These end points were determined quantitatively with questionnaires using a web-based survey tool and qualitatively by 2 rating conferences conducted via Zoom [42-44].

#### Quantitative

The usability, including internet use and comprehensibility of the questionnaires in the web-based survey tool, was tested by a local study team member before study onset, and changes were made accordingly [43]. The user satisfaction questionnaire contained 8 questions on a 5-point Likert scale displayed on 1 page; therefore, 40 points were achievable in the overall user satisfaction. The usability of the modules was tested according to the System Usability Scale (SUS), a validated usability score from 0 to 100, in which 68 could be interpreted as an average according to a curved grading scale [44]. The two knowledge gain tests assessing short- (knowledge gain test 1 [KT1]) and long-term (knowledge gain test 2 [KT2]) knowledge gain were composed of 15 multiple-choice questions, where each question counted as 1 point. The knowledge questionnaires displayed 1 question per page, resulting in 15 pages per test. The knowledge questions were all answerable with the presentation and partly derived from questions of German medical exams because questions on COPD from previous Zambian medical exams were not available. The answers could be reviewed and changed with the back button. All questions in web-based questionnaires had to be completed to submit the results. The questionnaires can be found in Multimedia Appendix 4.

#### Qualitative

When assessing a teaching intervention, qualitative data from rating conferences can shed light on the quantitative findings. This method is based on school quality assessments. The results of quantitative evaluation data are displayed to a representative group of up to 12 students, and the following discussion provides in-depth insight into individual motivations and opinions of the participants [42].

### Quantitative Evaluation

With the support of local study team members (AS and PA) and the local study coordinator (MM), the principal investigator (ES) conducted recruitment, randomization, and actual implementation of the trial from Germany. This was possible, as everything was conducted on the web because of the COVID-19 pandemic.

#### Recruitment and Randomization

All eligible students were invited to participate via email on April 20, 2021, and recruitment continued until April 30, 2021 (see Multimedia Appendix 5 for the study information sheet). Email addresses were obtained from the ML course coordinator (AS). Compensation for study participation and internet use related to the study were airtime vouchers, with a value of 200 Zambian Kwacha (US $10.9), to be received at the end of the entire study period.

Students willing to participate sent informed consent via email. Afterward, all participating students were equally randomized into the intervention and control groups using the random number function in Excel (Microsoft Corporation) and a blocked randomization list with a block size of 2 participants [45].

Participants were informally blinded to their group allocation for the first part of the study, as it was not stated in the information sheet which e-learning methods were being compared.

#### Phase 1: Evaluation of Satisfaction, Usability, and Short-term Knowledge Gain

Following randomization, the participants were invited on May 1, 2021, to participate in their respective e-learning module that was accessible using their e-learning platform account. The e-learning module could be studied asynchronously with the e-learning platform and application. Study participants only had access to their respective e-learning modules. Participants were informed that completing the e-learning module and filling the questionnaires would take approximately 45 minutes, but no time limit was set. Participation reminders were sent on May 7, 11, and 20, 2021, and through local study team members by class representatives. The e-learning platform was down for a few hours on May 4 and 7, 2021, but participants were given until May 31, 2021, to complete these tasks. It was possible to contact the principal investigator via email and a local study coordinator during the entire study. In the case of nonsolvable technical difficulties with the e-learning platform or the internet, individual students were sent a link to their respective e-learning module that was uploaded onto the cloud, whereas students in the control group received a PDF file [46]. The latter was not possible for students in the intervention group, as the interactive presentation could not be saved as a PDF file. Study participants receiving the cloud link or PDF file were asked not to share the information with other participants.

After finishing the module, each participant was directly invited to complete the user satisfaction, SUS, and KT1 questionnaires on the web [43]. Participants stated their study ID in web-based questionnaires to protect personal data. They were asked not to use the presentation or any other additional help to answer the questions. As their log-in information to the e-learning module was not verified, participants were considered to have completed their respective e-learning modules by filling out the web-based questionnaires.

Participants who dropped out of the study because they could not complete phase 1 were labeled *initial study dropouts*, whereas participants who completed it were *first-part participants*. First-part participants were categorized as *early responders* if they completed the module directly or after 1 reminder and as *late responders if* they completed the module and survey after 2 or more reminders.

#### Phase 2: Evaluation of Long-term Knowledge Gain

Four weeks after phase 1, on June 28, 2021, the first-part participants were invited to complete the KT2 [43]. They were asked not to use the e-learning module or any other resource for help.

#### Data Extraction

Pseudonymized data from the web-based questionnaires were automatically transferred to an Excel spreadsheet, thereby maintaining data integrity and security, and then prepared for statistical computing.

#### Analysis

##### Overview

Statistical analysis of the quantitative data was performed using the programming language R (version 4.0.3; R Foundation for Statistical Computing) and the packages *psych* and *likert* [47,48]. A *P* value <.05 was considered statistically significant. Cohen *d* was assessed using a web-based tool, and a post hoc power analysis was calculated with the program G*Power (version 3.1; Erdfelder, Faul, and Buchner) [49].

##### Characteristics of Study Participants

Only participants who completed the web-based questionnaires and therefore were considered to have completed their respective e-learning module were analyzed for primary and secondary outcomes, resulting in a modified intention-to-treat analysis. Characteristics of first-part participants and initial study dropouts, as well as characteristics of rating conference participants, were compared using a 2-tailed *t* test and responding chi-square tests.

##### Quantitative Comparison of the Two Modules

Differences in questionnaire results between the intervention and control groups were evaluated using the Mann-Whitney *U* test. The difference between the two knowledge gain tests’ scores of each group was calculated using a paired Wilcoxon test.

##### Factors Influencing Satisfaction, Usability, and Knowledge

We used linear regression, the Mann-Whitney *U* test, and the Kruskal-Wallis test to analyze whether several factors influenced overall user satisfaction, system usability, and knowledge gain test scores. If a factor with >2 subgroups, such as study year (second, third, and fourth), had a statistically significant influence on the questionnaire result, multiple pairwise comparisons were calculated using the R-function *pairwise.wilcox.test*.

### Qualitative Evaluation

#### Overview

ES recruited rating conference participants by email and acted as a moderator. Before the study commenced, she had no relationship with the rating conference participants. Additional participants present were 4 extra study team members, including AB and FN, for transcription purposes. They took field notes and audio recordings, which ES later used for transcription.

#### Recruitment

Approximately 2 weeks after the first study period, on June 16 and 17, 2021, a total of 2 rating conferences took place via Zoom. More than half of the first-part participants (24/41, 59%) were invited to receive sufficient data saturation. Participants in the rating conferences were purposively sampled to be representative of the overall study population that completed the first part of the study. The purposive sampling was stratified for each allocation group according to sex, time of participation (early responder and late responder), and age (<25 years and >25 years), as the mean age (24.3, SD 4.8 years) of first-part participants was approximately 25 years. Rating conference participants received an additional airtime voucher (200 Zambian Kwacha) as compensation.

#### Phase 3: Conducting the Rating Conferences

Quantitative results from the web-based questionnaires were presented in the rating conferences, which lasted 60 minutes each. The following discussion was semistructured into four parts: satisfaction, usability, knowledge, and e-learning. Each subpart commenced with open questions from the moderator and probes, where appropriate. The semistructured interview guide was not pilot-tested; it was, however, internally reviewed, and a final version was agreed upon by the research team. An active discussion among all participants of the rating conferences was encouraged.

#### Analysis

The principles for coding and analyzing data were determined in advance. The determined codes and themes were not dependent on their prevalence in the entire data set but rather established through salience in the data. The analysis focused on a detailed description of the data using inductive data-driven analysis. Semantic rather than latent themes were identified, and finally, the analysis was approached in a realist manner, implying what was said was directly linked to its meaning [50]. The data were analyzed using thematic analysis, according to Braun and Clarke [50]. FR and ES examined the data set and identified codes and themes, which were structured into a preliminary coding tree using the NVivo program (version 12; QSR International). The coding tree was then finalized through continuous review of the data set, codes, and themes and an ongoing discussion between the two researchers responsible for data analysis. Afterward, the final coding tree was used by both researchers independently to code the data set again, and any discrepancies were discussed collaboratively. The final coding tree consisted of the following structure: the comparison of the e-learning modules regarding satisfaction, usability, and knowledge, access to the e-learning material, opinions on e-learning and improvement suggestions, and study limitations.

## Results

### Quantitative Evaluation of Phases 1 and 2

#### Characteristics of Study Participants

**Figure 1:**
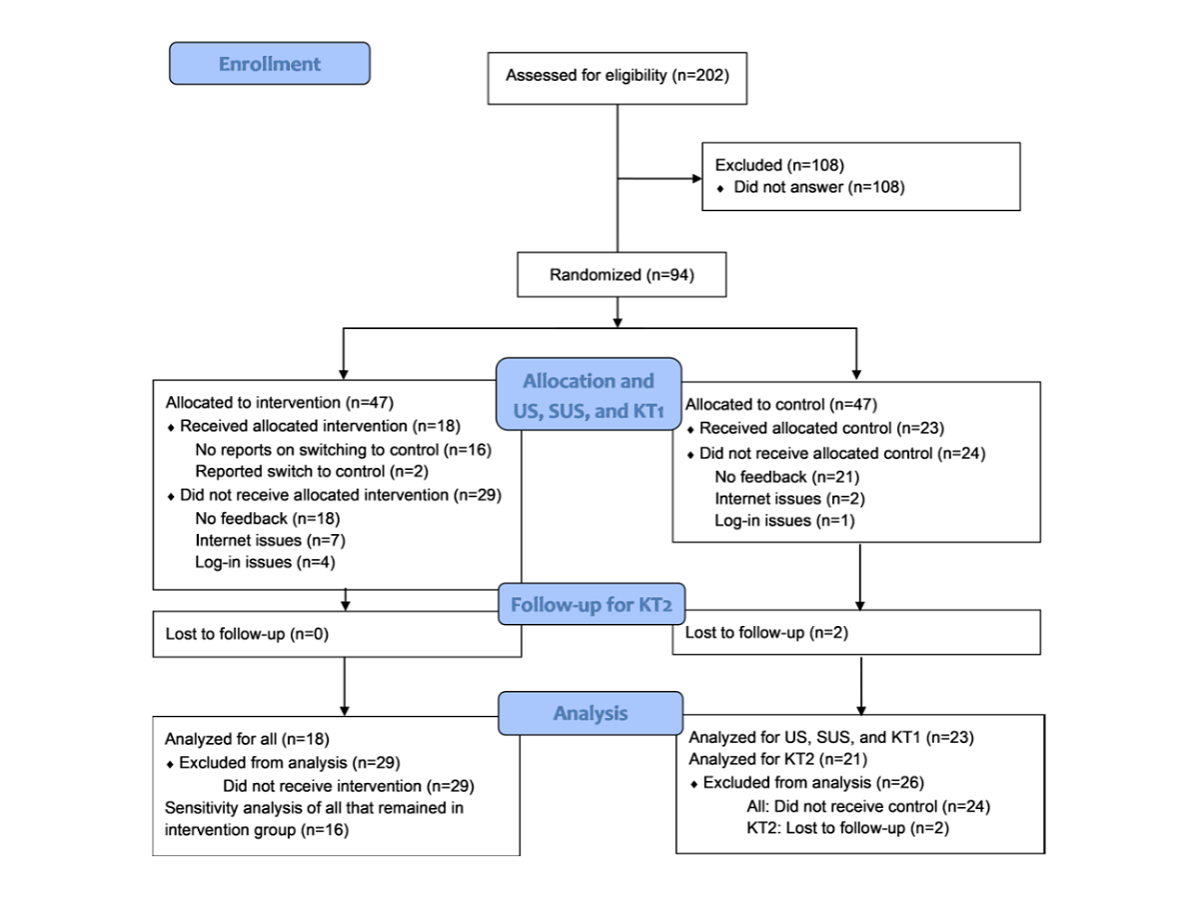
CONSORT (Consolidated Standards of Reporting Trials) 2010 flow diagram. KT1: knowledge gain test 1; KT2: knowledge gain test 2; SUS: System Usability Scale; US: user satisfaction.

In total, 202 ML students, predominantly in their second year of study, were identified as eligible for participation. Of these, 47% (94/202) of the students signed up for the study. Ultimately, 44% (41/94) of these students participated in the first part of the study and were analyzed. The participant flow and reasons why enrolled participants did not complete the e-learning module and questionnaires (initial study dropouts) are shown in Figure 1. If a student had filled out the web-based questionnaire, it was assumed that they had also received their allocated intervention or control. In all, 2 students in the intervention group later reported in the rating conference that they had switched groups; however, a post hoc sensitivity analysis that excluded these 2 students revealed no differences in the outcomes. The KT2 was completed by 39 first-part participants, as 2 students were lost to follow-up. All participants who started filling out web-based questionnaires also completed them.

Table 1 shows the characteristics of first-part participants, initial study dropouts, and first-part participants in intervention and control groups. There were significantly more female students that were enrolled but did not complete the first part of the study. Apart from that, characteristics did not differ significantly.

**Table 1:** Characteristics of first-part participants, initial study dropouts, and first-part participants in intervention and control groups.

Characteristics		First-part participants (n=41)	Initial study dropouts (n=53)	*P* value		Intervention (n=18)		Control (n=23)
Age (years), mean (SD)		24.3 (4.8)	23.4 (5.4), n=40	.44		23.6 (3.5)		24.9 (5.7)
Sex (female), n (%)		14 (34)	34 (64)	.007		6 (33)		8 (35)
Group (intervention), n (%)		18 (44)	29 (55)	.41		N/A^a^		N/A
**Study year, n (%)**					.53			
	2	29 (71)	40 (75)			14 (78)		15 (65)
	3	5 (12)	8 (15)			2 (11)		3 (13)
	4	7 (17)	5 (9)			2 (11)		5 (22)

^a^Not applicable.

#### Quantitative Comparison of the Two Modules

##### Primary Outcome: User Satisfaction

**Figure 2:**
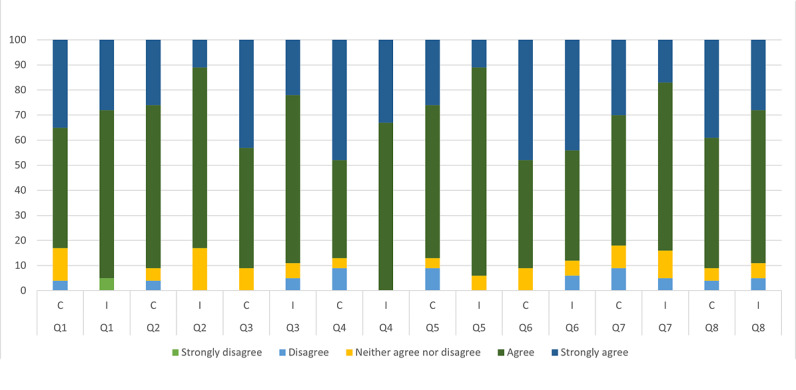
Results of user satisfaction questions of the intervention and control groups in percent. Q1: I enjoyed the module. Q2: I am satisfied with the module. Q3: My COPD knowledge increased significantly. Q4: My interest in COPD increased. Q5: Module’s key messages were clear. Q6: Module is relevant for medical practice. Q7: It was easy to learn with the module. Q8: I would recommend the module to a friend. C: control; COPD: chronic obstructive pulmonary disease; I: intervention Q: question.

**Figure 3:**
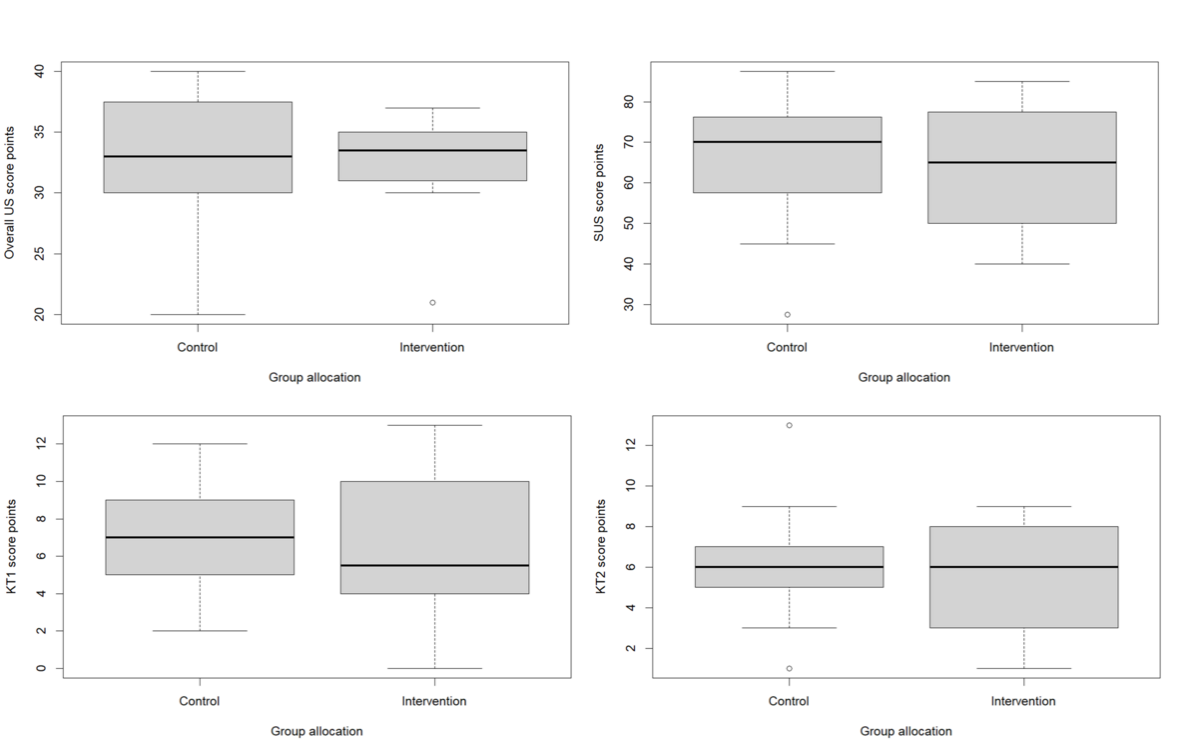
Box plots of different questionnaire results of the intervention and control groups. Box plots show median, first quartile, third quartile, minimum, maximum, and outliers. KT1: knowledge gain test 1; KT2: knowledge gain test 2; SUS: System Usability Scale; US: user satisfaction.

Results for user satisfaction were not statistically different between the intervention and control groups (Table 2). Bar plots of each user satisfaction question result for both groups are shown in Figure 2. Figure 3 depicts, among other things, the overall user satisfaction scores of the intervention and control groups in a box plot.

**Table 2:** Questionnaire results.

Parameters	Intervention (n=18)	Control (n=23)	*P* value
User satisfaction (n=41), median (Q1,^a^ Q3^b^)	33.5 (31.3, 35)	33 (30, 37.5)	.66
System Usability Scale (n=41), median (Q1, Q3)	65 (50.6, 76.9)	70 (57.5, 76.3)	.36
KT1^c^ (n=41), median (Q1, Q3)	5.5 (4, 9.3)	7 (5, 9)	.26
Self-reported time for e-learning module (minutes; n=41), median (Q1, Q3)	51.5 (45, 60)	55 (40, 63)	.92
KT2^d^ (n=39), median (Q1, Q3)	6 (3, 7)	6 (3.3, 7.8)	.88
KT^e^ difference (test 1-2), n=39, median (Q1, Q3)	0.5 (−2, 3)	0 (−1, 5)	.58

^a^Q1: first quartile.

^b^Q3: third quartile.

^c^KT1: knowledge gain test 1.

^d^KT2: knowledge gain test 2.

^e^KT: knowledge gain test.

##### Secondary Outcomes: Usability and Knowledge Gain

The SUS and KT1 scores and self-reported time spent learning with the e-learning module did not differ statistically significantly between the intervention and control groups (Table 2). However, the data indicated that the intervention group stated slightly lower system usability and received a slightly lower KT1 score. In addition, there were no statistically significant differences in the KT2 and knowledge test scores between the two groups. The sample size for these 2 analyses was 39, as 2 participants were lost to follow-up. Furthermore, each group had a knowledge test score difference close to 0, and the analysis also confirmed that the KT1 and KT2 scores of each group were not significantly different. Figure 3 shows the different questionnaire scores of the intervention and control groups in boxplots.

#### Factors Influencing Satisfaction, Usability, and Knowledge

The influence of the following factors on user satisfaction, SUS, KT1, and KT2 scores was evaluated: additional study resources, age, device, participant environment, response time for study participation, sex, study year, and time spent learning (Table 3). The influence of the study year on the SUS score was statistically significant. Further analysis revealed a significant difference in SUS scores between second- and fourth-year students, and fourth-year students correlated with a higher SUS score. In addition, a significant correlation was found between the study year and KT1 score. However, when testing for multiple pairwise comparisons to further determine which study years differed significantly in their KT1 scores, no statistically significant differences were found. Most likely, the difference in KT1 score between the second and third study years caused the overall significant correlation, as third-year students had a higher median KT1 score than second-year students, and the *P* value of that combination was the lowest at .06.

**Table 3:** *P* values of correlations between different factors with questionnaire results.

Factors	US^a^ (n=41)	SUS^b^ (n=41)	KT1^c^ (n=41)	KT2^d^ (n=39)
Sex	.48	.37	.52	.48
Age	.45	.24	.85	.39
Study year	.17	.04	.03	.11
Response time	.08	.93	.88	.57
Environment	.20	.35	.76	.71
Device	.27	.44	.19	.11
Other resource	.25	.08	.07	.41
Time	.29	.15	.06	.49

^a^US: user satisfaction.

^b^SUS: System Usability Scale.

^c^KT1: knowledge gain test 1.

^d^KT2: knowledge gain test 2.

### Qualitative Evaluation of Phase 3

#### Characteristics of Study Participants

We invited 24 first-part participants to participate in the rating conferences (see *Methods* for sample size and recruitment procedures), of whom 54% (13/24) replied and participated in 2 rating conferences. Table 4 shows the characteristics of all rating conference participants and the rest of the first-part participants and the characteristics of both rating conference groups. No statistically significant differences were found among groups.

**Table 4:** Characteristics of rating conference participants versus other first-part participants and rating conferences.

Characteristics		Rating conference participants (n=13)	Other first-part participants (n=28)	*P* value	Rating conference 1 (n=7)	Rating conference 2 (n=6)	*P* value
Age (years), mean (SD)		26 (6.9)	23.5 (3.4)	.24	26 (6.1)	26 (8.3)	.99
Group (intervention), n (%)		8 (62)	10 (36)	.23	4 (57)	4 (67)	.99
Sex (female), n (%)		4 (31)	10 (36)	.99	3 (43)	1 (17)	.68
**Study year, n (%)**				.67			.88
	2	8 (62)	21 (75)		4 (57)	4 (67)	
	3	2 (15)	3 (11)		1 (14)	1 (17)	
	4	3 (23)	4 (14)		2 (29)	1 (17)	

#### Qualitative Results

In addition to their own views, participants also relayed the views of other participants absent in the conference as they had communicated with other study members. As the results of these 2 perspectives did not differ, they are presented together.

##### Primary Outcome User Satisfaction and Secondary Outcome Usability

Students often reported that it was their first time learning about COPD and expressed gratitude for the opportunity. Comparing the two e-learning methods, satisfaction and usability were linked and, therefore, assigned a category together. Participants reported challenges in accessing both e-learning modules, resulting in lower satisfaction:

I think it really affected my happiness because I don’t like ending things halfway or something taking that long.

Participant 7, control, female

The noninteractive module took a long time to load and sometimes just crashed while viewing the presentation; however, access to the interactive module seemed to be impeded even more as a few students from that group (3/8, 38%) reported that they were not able to finish learning with it or could not access it at all:

I was not able to get into anything.

Participant 5, intervention—changed to control, male

In addition, the interactive software was described as challenging to use once having gained access to it. This was mainly because of technical difficulties, as it was reported (4/8, 50%) that going back and reviewing the interactive e-learning module was difficult, and the graphics were poorly presented on students’ phones.

These access challenges using the interactive module led to intervention participants using alternative methods to learn about COPD. Further research on the web (2/8, 25%) or gaining access to the e-learning module of the noninteractive group (2/8, 25%) were reported:

I failed to use the interactive instead I managed to access the non-interactive.

Participant 5, intervention—changed to control, male

An explanation of how access to the noninteractive e-learning module was achieved was not given by the participants in question. An additional challenge for students in the intervention group was the limited e-learning experience with interactive e-learning:

Some people [said]: ‘ah I gave up’ after trying to use it.... Because some of them it was the first time having to use that interactive session. So, some of them didn’t even know they had to actually click some of those things.

Participant 9, intervention, male

A few students (2/8, 25%) reported that they would have preferred the noninteractive e-learning module because it was simpler, and the interactive e-learning module was deemed complicated:

I thought like it was a little bit clustered [cluttered]. Like I actually had to search around and see where exactly I have to go back to. So yeah otherwise, other than that I would have actually even preferred to have the PowerPoint one.

Participant 9, intervention, male

However, other students (3/8, 38%) declared being satisfied with the interactivity of the intervention module, as “it’s like you are having your lecturer right there” (participant 12, intervention, male). They enjoyed “the imagery parts where you could actually click on things” (participant 10, intervention, male).

Furthermore, rating conference statements showed that there was no gender dimension regarding access to interactive e-learning, and female and male participants struggled to access the interactive module alike.

##### Secondary Outcome Knowledge Gain

Both groups regarded the knowledge gain test’s difficulty as adequate. Nevertheless, there was a discrepancy between the participants’ views of its feasibility and the overall outcome. When confronted with the results, some participants (5/13, 39%) viewed the impeded access to the e-learning modules as the reason for the average marks of both groups:

I think the reason why the performance was average is probably because maybe the majority were not able to finish their modules, so I guess.

Participant 6, control, female

Nonetheless, more members of the interactive group (4/8, 50%) linked their increased barriers in accessing and using their e-learning module with their reduced knowledge gain test results:

I feel that the ones that had the control maybe they had a slightly easier way of going back to certain things that they had to read over.... I think if people had more experience to actually go back to the interactive sessions, I think there would have been better marks than that.

Participant 9, intervention, male

##### Secondary Outcome Barriers to e-Learning

**Figure 4:**
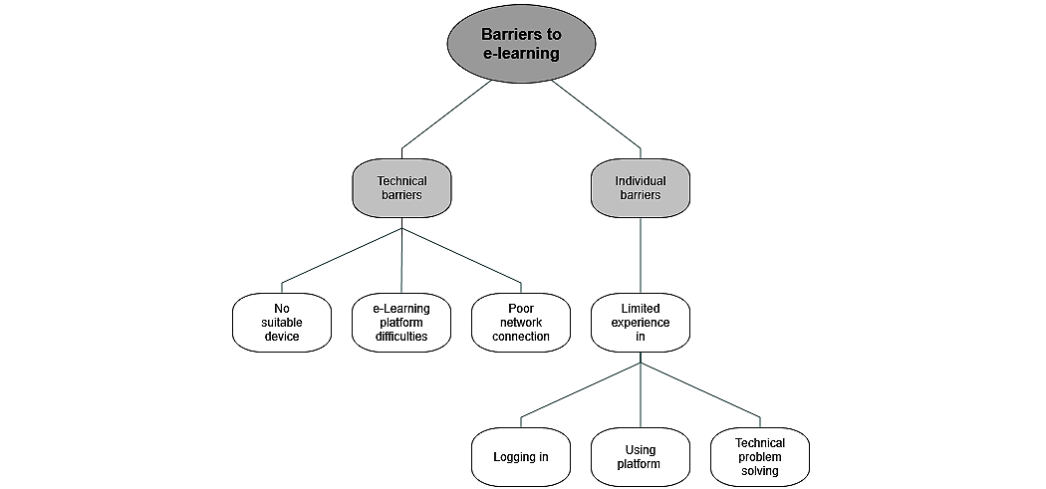
Barriers to e-learning.

A few barriers to access the e-learning material are mentioned above; however, the following results provide a more comprehensive overview. Figure 4 depicts the identified barriers, which can be divided into technical and individual barriers. Technical barriers identified were limited access to digital devices compatible with the e-learning platform, technical challenges with the e-learning platform, including log-in and the e-learning software itself, and internet access. Determined individual barriers occurred because of the limited e-learning experience of participants. These included limited knowledge on logging in to Moodle, using the e-learning platform and the software of the e-learning modules, and problem solving if a technical issue occurred. The difference between technical and individual barriers was that individual barriers were user generated.

A student reported that the tablets that were initially distributed when the e-learning program was implemented were not being used by him or by some of his fellow students. The reason was that the device “just lags and then it will fail to load” (participant 9, intervention, male). In addition, students (4/13, 31%) reported that access to other suitable electronic devices was difficult for some participants:

Yes, they did have smartphones, but not the ones that would load the e-learning module.

Participant 12, intervention, male

Some students encountered technical challenges when trying to log in to the e-learning platform (2/13, 15%), as they could not generate new passwords themselves:

It took me quite a, I think a few days. I had to actually get in touch with the HIGH IT personal from the university to actually help me with my username and my password.

Participant 9, intervention, male

When fellow students of this particular participant heard that he was able to log in, they “were actually shocked to say how did you manage?” (participant 9, intervention, male).

Furthermore, the software of the e-learning modules was considered a barrier in various ways. Participants often reported that the modules were “taking long to load” (participant 12, intervention, male) or that the system “just froze...it didn’t have anything to do with the network” (participant 7, control, female). Once they had gained access to the e-learning materials, some participants (4/13, 31%) stated difficulties in going back in the presentation or viewing the graphics on their phones. This was mainly the case for the interactive e-learning modules. Often, study participants (5/13, 39%) reported that difficulties vanished when using a larger electronic device, such as a laptop or desktop computer:

So I had the same experience when I used my phone, but when I switched to the PC it was like working.

Participant 12, intervention, male

I needed to use a laptop I think for me to have access.

Participant 1, intervention, male

Participants (5/13, 39%) stated that the internet connection posed another barrier to accessing the e-learning modules. The connection had to be fast, loading the modules took a long time, and some students were located in areas with very limited internet access. When asked why there were so many study dropouts, a participant replied the following:

For the people that I got to ask, one of them was in an area that had really horrible network. So, she only got the email like time after the whole participation thing had passed. So maybe the main reason was that everything had network issues and maybe things were not syncing or loading as fast as some people, because they were in a different area.

Participant 7, control, female

The use of the e-learning platform Moodle, and consequently students’ e-learning experience with it, was reported to be low. Other methods of web-based learning, although not asynchronous, were used during the COVID-19 pandemic:

We once tried to use Moodle at the school, but it never worked out, so we switched to Zoom or Google Meet.

Participant 12, intervention, male

This limited experience frequently impeded participants’ access as they forgot their e-learning platform log-in details and had restricted knowledge about the e-learning platform and software or technical problem solving if an issue emerged:

So, others had forgotten how to use it. So, I find instead of putting their username, they were putting in the email address with the correct password. So, they were failing to login.

Participant 1, intervention, male

Most of the people that we have in our class haven’t used the e-learning modules or used Moodle. So, they had challenges with navigating through.

Participant 9, intervention, male

##### Opinions of e-Learning and Suggestions for Future Improvement

It was evident that, despite the access challenges, students’ motivation and their opinions regarding e-learning were positive, especially in a pandemic context:

I think it’s actually a good development. And I think it would help, especially in this time where we are actually battling with Corona. It would actually help. And then it gives you also a chance to actually do it at your own time and you don’t feel rushed. So, you actually prepare for it.

Participant 9, intervention, male

However, it was also mentioned that asynchronous e-learning was fairly difficult and lacked interaction with teachers (5/13, 39%):

It would have been better if there was someone explaining it.

Participant 7, control, female

A student’s opinion was that e-learning “can work as a backup where physical learning is not possible due to limited space or as a way of revising with students” (participant 11, control, male).

Finally, participants gave several suggestions for improvement, such as developing an e-learning software compatible with their phones or otherwise access to suitable gadgets, improving the log-in to the e-learning platform, and using more e-learning, which should also be more standardized in its presentation:

And I think with a little bit more usage I think I would get experience in terms of how to really navigate it well and yeah. I think that’s the issue. I think using it more and not having issues with the logging in, I think would really, really help.

Participant 9, Intervention, Male

I just feel like if it could be more consistent just for people to get a grip of it that would be nice.

Participant 8, control, male

## Discussion

### Comparison of Interactive Versus Noninteractive e-Learning

#### Primary Outcome User Satisfaction and Secondary Outcome Usability

##### Principal Findings and Explanations

In contrast to the initial hypothesis derived from studies on HICs, there were no significant differences among the groups in the primary outcome of user satisfaction in this low-resource setting [13,14,18,19]. This suggests that both modules were received similarly. The overall user satisfaction in both groups was acceptable. The median SUS score of both modules was assessed as average. Furthermore, there was no significant difference in SUS scores between the two modules, implying that both were equally challenging to use. However, contrary to the quantitative data, qualitative data showed that the interactive e-learning module had lower usability than the noninteractive module. The interactive module was harder to access, as multiple students could not finish it, it was not correctly displayed on the phones, and revising it was difficult. The interactive module was also harder for some students, as they were not familiar with interactive e-learning. Qualitative data also indicated that usability challenges negatively influenced students’ satisfaction with the modules, thereby linking these 2 distinct outcomes. There are several possible explanations for the lack of differences in user satisfaction between the two groups.

A reason could be an insufficient number of study participants to show an effect. Owing to many dropouts, the size of the analyzed population was limited. Furthermore, the difference in user satisfaction between both groups was small, and a post hoc power analysis revealed a low power of 7%, leading to the conclusion that quantitative data might be insufficient to prove or disprove the assumed hypothesis.

Another reason could be that the increased usability issues of the interactive module may have had a negative effect on the user satisfaction rating, as indicated by the qualitative data. Gunesekera et al [51] conducted a literature review that supports this assumption on the relationship between usability and satisfaction. Better usability results in a higher motivation to learn [52]. Nevertheless, the correlation is not as simple as it seems. Davids et al [53] conducted a study in South Africa using a similar approach. However, they compared their original interactive e-learning module with a revised version in which all usability issues were addressed. Yet, comparable with this study, there were no significant differences in satisfaction, usability, and knowledge gain between the two groups. When analyzing the objective usability through a video of the study, however, there were significantly fewer problems in the intervention group, resulting in objective usability differences among groups. When assuming that there were indeed usability differences but no user satisfaction differences between the two groups, the results of Davids et al [53] contradict the conclusion of the literature review by Gunesekera et al [51].

Another explanation for the lack of quantitative difference in satisfaction between the two groups could be that the participating students were more familiar with traditional teaching methods and less familiar with interactive e-learning than students in HICs [7]. Consequently, this could impede the rating of satisfaction and usability of interactive materials. The qualitative data of this study further supports this interpretation, as some participants in the intervention group were overwhelmed with the interactive technology or preferred the noninteractive presentation because it was simpler, possibly because of a lack of experience with interactive e-learning. Additional evidence for this was that fourth-year students rated the usability of their e-learning modules significantly higher than second-year students. They might have been exposed to e-learning technology longer and therefore found it easier to use.

Finally, as the 8 user satisfaction questions selected were not validated, they may not have accurately portrayed user satisfaction.

##### Comparison With Previous Work

When considering these results in context with the existing literature, studies with similar findings are rare. Nevertheless, most studies use distinct tools to assess user satisfaction, which limits comparisons. For the most part, studies that compared the user satisfaction of interactive and noninteractive e-learning for health care personnel demonstrated results in favor of the interactive e-learning method [13,14,18,19,54]. However, they were mostly conducted in HICs. Koka et al [14] provided an example of this. Their study was conducted in Switzerland and showed that paramedics undergoing an interactive e-learning module had increased knowledge of the National Institutes of Health Stroke Scale and higher satisfaction with the learning method than paramedics watching a video of the same learning content [14]. Another example is the RCT implemented by Lee et al [19] in Taiwan. In this study, undergraduate medical students were randomized to receive an interactive multimedia module or PowerPoint presentation slides. Although no significant difference in knowledge gain was observed among groups, the intervention group received significantly higher user satisfaction scores [19]. Nevertheless, there are studies that, as this study, show no difference in user satisfaction, comparing interactive with noninteractive e-learning [55,56].

Overall, the results of this study invoke the question of whether ease of use is a more important factor for user satisfaction than content presentation. Given that this study’s findings differed from the conclusions of similar studies in HICs, they further raise the question of equity in access to knowledge and education via e-learning in LMICs.

#### Secondary Outcome Knowledge Gain

##### Principal Findings and Explanations

Both groups received low to average KT1 and KT2 scores. This could indicate that both e-learning modules were not able to convey as much information as expected. However, another possibility is that the knowledge tests did not measure the true knowledge as they were not validated.

An additional finding of this study was that there was no significant difference between the KT1 and KT2 scores of each group. Assuming that both knowledge tests were equally challenging, this indicates that there was no significant knowledge loss after 6 weeks for both groups. This result could be interpreted as an advantage for both e-learning courses. However, it was not compared with a group that only received traditional classroom teaching, for example, and therefore cannot be contextualized.

Contrary to other studies, the analysis of this work also revealed no significant difference in short- or long-term knowledge gain between the two groups [13-18]. This was potentially related to qualitative data, which indicated that impeded access to the interactive e-learning module made it harder for students in the intervention group to learn the material or even look up information during the knowledge test. Participants were told not to use any material to help answer the knowledge questions; however, this was not verifiable.

##### Comparison With Previous Work

There have been several RCTs, including the one by Koka et al [14] that postulate interactive e-learning increases knowledge better than noninteractive e-learning. However, they were all conducted in HICs. Velan et al [17] showed in a randomized crossover trial that interactive e-learning modules were significantly more effective in improving medical students’ knowledge about the adequate use of imaging than PDF-based modules. DeBate et al [15] compared an interactive e-learning module for secondary prevention of eating disorders using a flat-text e-learning module in an RCT. They concluded that the interactive module was better at improving students' skill-based knowledge and self-efficacy but not overall knowledge [15]. Morgulis et al [16] demonstrated in an RCT that an interactive e-learning module significantly increased knowledge about leukemia better than existing web-based resources in senior medical students.

However, it seems that the hypothesis does not always hold true. Apart from the RCT by Lee et al [19], other studies provide additional examples. Suppan et al [55,56] conducted 2 web-based RCTs with student paramedics and emergency medicine personnel in Switzerland. The intervention group received a gamified e-learning module about personal protective equipment for COVID-19, whereas the control group received flat-text COVID-19 guidelines for prehospital emergency medicine use. The primary end point was the difference in postintervention knowledge between the two groups, and, as in this study, it was not statistically significant. Another study conducted with Canadian medical students compared an interactive e-learning module on global health with PDF articles on the same topic. Although participants’ satisfaction with the interactive module was higher, no difference in postintervention knowledge was detected [54].

### Barriers to e-Learning

#### Principal Findings and Explanations

There were 56% (53/94) of study dropouts, possibly because of problems accessing the e-learning modules. The identified barriers to e-learning were of a technical and individual nature. Technical barriers included limited access to suitable electronic devices and difficulties with the e-learning platform, including log-in and software issues (eg, long loading times, crashing, and poor graphics presentation). An additional technical barrier was insufficient internet access. The e-learning platform can also be used via an application that would have probably increased the technical usability; however, this was possibly not known to all study participants. Because of the COVID-19 pandemic, the small information technology (IT) support team at LMMU was overwhelmed by many tasks when participants needed access to the e-learning platform. This may explain the insufficient capacity to instruct all students before the study. Individual barriers may be summarized as limited *e-learning culture* owing to low e-learning use and encompassed restricted e-learning experience in logging in to the e-learning platform, using the e-learning platform and software, and technical problem solving if technical issues occurred. In addition, the lack of communication with teachers was often viewed as having a negative impact. Among the study dropouts, there was a significantly higher number of female students, which may indicate that this student group was more affected by these barriers. A possible reason could be inadequate technology experience, as a questionnaire in 2017 indicated that female ML students had low technology experience, whereas male ML students had moderate experience [23].

It is assumed that had this study been conducted on campus, some of these hindrances, especially regarding the e-learning infrastructure (suitable devices and internet), could have potentially been avoided. However, because of the COVID-19 pandemic, participants had limited access to facilities at the LMMU campus.

#### Comparison With Previous Work

Most of the identified barriers, such as poor e-learning infrastructure, including device and internet availability or insufficient interaction with a teacher, are well known in the literature on e-learning in LMICs, and some are known from previous studies at the LMMU [1,4,22]. An example is a survey in the Philippines that assessed barriers encountered by medical students when trying to learn on the web after the COVID-19 pandemic had just hit. Identified barriers also included limited access to electronic devices and the internet. However, students also struggled to adapt to the new learning method [57]. This may suggest that, as in this study, some barriers to e-learning in LMICs are set beyond the technical infrastructure, as they might also be dependent on the individual characteristics of e-learning students. These individual barriers may be inherent to nascent e-learning systems in a low-resource context.

#### e-Learning Use

Barteit et al [22] assessed e-learning platform use as low in 2017. Unfortunately, this appears unresolved, as some participants reported that they did not use the e-learning platform to study. Furthermore, most students had e-learning platform accounts but had not used them regularly, so some had forgotten their log-in details. Explanations for this low use are difficult to discern because of the various stakeholders involved in an e-learning system. In 2017, reasons included the low quality of the tablets, insufficient e-learning training for students and lecturers, and average quality of the e-learning material, with low motivation of teachers to update and improve the content [22]. As the aims of this study did not include the evaluation of the use of the e-learning platform, only assumptions can be made for low use. Several factors should be considered to promote e-learning use in a low-resource context, some of which may be applied insufficiently at the LMMU: up-to-date information should be conveyed in the e-learning material, the practicality of e-learning should be advocated while e-learning services should be expanded, e-learning should be user friendly, sufficient technology training should be provided to students and lecturers, and individual motivation toward e-learning should be increased to promote overall e-learning use [7]. The IT resources during the implementation of this study were strained, meaning there may be insufficient IT resources to promote these factors to increase e-learning use at the LMMU.

### Strengths and Limitations

This study is the first to compare interactive and noninteractive e-learning for students in clinical sciences or comparable studies in Zambia and one of the first known in a lower-middle–income country. As the value of e-learning in low-resource countries is increasingly recognized, especially during the COVID-19 pandemic, it is important to assess different e-learning methods in these settings, and the mixed methods design of this study allowed a comprehensive overview of the subject. However, this study had several limitations. They can be structured into general study limitations, limitations associated with the web-based study format, and shortcomings of the e-learning module comparison.

The analyzed study sample might be biased because only users were evaluated and not the original sample (because of many dropouts). This also resulted in a small sample size of the analyzed population and low post hoc power. However, the sample still seems to represent the overall group of students in the ML course quite well. In addition, the principal investigator developed the e-learning content, which could have affected the results. Social desirability could have also influenced participants’ statements in rating conferences, as the principal investigator was also a rating conference moderator. We attempted to circumvent this bias in qualitative data acquisition by repeatedly asking the participants for their honest opinions, building rapport, and probing for details.

Because of the COVID-19 pandemic, this study was conducted on the web, which poses further limitations. Recruitment was completed by email, which could have limited the number of students enlisted. Furthermore, insufficient internet access and connectivity may have affected the students’ completion of the web-based questionnaires and communication in the rating conferences because of dialogue loss.

Qualitative data suggested that the interactive module was more difficult to access and use; therefore, the comparison of the two e-learning modules was likely limited by the experienced technical problems. In addition, some students gained access to the other e-learning module but were analyzed for their originally assigned module; however, a post hoc sensitivity analysis that excluded these 2 students showed no differences in the assessed outcomes. Finally, participants may have looked up answers on the web, done teamwork, or unblinded themselves through conversations with other participants. Although such behavior affects outcome variables, it is most likely a reflection of learning in real-world circumstances.

### Suggestions for Further Research

Secondary results suggested that the current relevant question may not be interactive versus noninteractive e-learning at the LMMU but the ease of access to e-learning. Although students’ motivation for e-learning was high, the e-learning program at the LMMU still faces several challenges. These can and should be addressed through further e-learning training for all students and lecturers and the promotion of continuous implementation of e-learning as an integral part of the curriculum. Increased use, in turn, would likely help improve the user experience of the e-learning platform. Additional resources should be allocated for IT personnel and infrastructure, if possible and needed. Future studies comparing interactive and noninteractive e-learning for health care personnel in low-resource settings such as Zambia should ensure that potentially limiting factors in the technical access to e-learning materials are mitigated. This could be achieved by uploading the study content for offline use to a set number of tablets. However, this would likely decrease external validity.

### Conclusions

In contrast to previous studies conducted in HICs, interactive and noninteractive e-learning were not significantly different in terms of user satisfaction and knowledge gain. However, these results may not be generalizable to other low-resource settings because the post hoc power was low, and the e-learning system at the LMMU has not yet reached its full potential. Consequently, barriers to accessing e-learning, which were of a technical and individual nature, may have affected the results, particularly as the interactive module was deemed harder to access and use. The extent to which some limitations were inherent to the nascent e-learning system, as opposed to the result of impaired e-learning access, is difficult to assess. Future studies should minimize technical e-learning barriers to further evaluate interactive e-learning in LMICs.

## Appendix

Multimedia Appendix 1 CONSORT-EHEALTH checklist (V 1.6.1).

Multimedia Appendix 2 Screenshots of the noninteractive module [25,38,39].

Multimedia Appendix 3 Screenshots of the interactive module [25,38,39].

Multimedia Appendix 4 Questionnaires.

Multimedia Appendix 5 Study information sheet.

## Abbreviations

BLiZ: Blended Learning in Zambia
CHERRIES: Checklist for Reporting Results of Internet E-Surveys
CONSORT-EHEALTH: Consolidated Standards of Reporting Trials of Electronic and Mobile Health Applications and Online Telehealth)
COPD: chronic obstructive pulmonary disease
COREQ: Consolidated Criteria for Reporting Qualitative Research
GOLD: Global Initiative for Chronic Obstructive Lung Disease
HIC: high-income country
IT: information technology
KT1: knowledge gain test 1
KT2: knowledge gain test 2
LMIC: low- and middle-income country
LMMU: Levy Mwanawasa Medical University
ML: medical licentiate
RCT: randomized controlled trial
SSA: sub-Saharan Africa
SUS: System Usability Scale
