# Supplementary material for: Real-life Evaluation of an Interactive Versus Noninteractive e-Learning Module on Chronic Obstructive Pulmonary Disease for Medical Licentiate Students in Zambia: Web-Based, Mixed Methods Randomized Controlled Trial
Source: JMIR Med Educ. 2022 Feb 24;8(1):e34751. doi: 10.2196/34751 (PMC8914755; doi:10.2196/34751)
Supplement: Multimedia Appendix 2 [file mededu_v8i1e34751_app2.pdf]

Screenshots non-interactive module

COPD

Chronic Obstructive Pulmonary Disease

Introduction

Welcome to our e-learning module about COPD! Please make sure to be in a quiet environment and that you have enough time to complete the course as well as answer a few questions afterwards. Altogether it should take about 45 minutes. Thank you for participating!

Time

Please write down the time it is now. You have as much time as you want to complete the module. We just would like to know how much time you had to invest in the end.

Course content

- Definition
- Epidemiology and Etiology
- Pathogenesis
- Symptoms
- Diagnosis
- Classification of severity
- Differential diagnosis
- Therapy
- Prognosis

Definition

- COPD= Chronic obstructive pulmonary disease
- A chronic, not curable but preventable and treatable lung disease with persistent respiratory symptoms and irreversible airflow obstruction
- The cause is an inflammation reaction of the lungs due to exposure to noxious particles or gases

Epidemiology

- No epidemiological data on COPD in Zambia can be found
- In 2010, global prevalence was 11.7%
- In 2015, 3.2 Million deaths were attributed to COPD worldwide (3rd leading cause of death in adults worldwide)

→90% of global deaths occur in Low-and Middle-Income countries (LMICs)

- Prevalence is predicted to rise, especially in LMICs, due to increased smoking rates and an aging population
- More men than women are affected but women are slowly catching up due to increased smoking rates

Etiology (1)

- Mix of environmental and genetic (host) factors
- Environmental factors:

  1. Smoking → is in 90% of the cases the cause for COPD worldwide

  2. Indoor and outdoor air pollution: especially indoor air pollution through burning of biomass (charcoal, dung, wood) → within LMICs people with a lower socioeconomic status and women are more affected

  3. Abnormal lung development due to for example early childhood lung infections or low birthweight

  4. Abnormal lung function due to Asthma, history of Tuberculosis or recurrent respiratory infections

Etiology (2)

- Genetic/ host factors:

  1. Alpha 1 –Antitrypsin deficiency → COPD patients under 50 years should be tested for this genetic abnormality
  2. Age → CODP prevalence increases with age

Pathogenesis

- Chronic inflammation in small airways, induced by inhaled noxious agents

→Airway remodeling: fibrosis and loss of parenchyma, bronchial instability and hypersecretion of mucus

→Over time typically fixed obstruction with collapse of bronchioles/bronchi during forced expiration

→Hyperinflation of lung (lung emphysema) with reduced ventilation and consequently pulmonary hypertension and chronic cor pulmonale

Symptoms

- Chronic cough (may be purulent during infectious exacerbations)
- High sputum production
- Dyspnea (initially while exercising, later constantly)
- History of repeated lower respiratory tract infections
- Pursing of lips: during expiration patient closes lips slightly → by breathing out with resistance, air trapping in the lungs is prevented by maintaining intrabronchial pressure → prevents collapse of small airways

Diagnosis

- Patient history (risk factors, symptoms…)
- Physical examination:

➢ signs of emphysema: barrel chest (form of thorax), cyanosis, silent chest (decreased breath sounds), hyper resonant percussion sound, coarse crepitations, expiratory rhonchi…

- Usually: when symptoms and risk factors present → spirometry
- Other possible tests (for differential diagnoses): chest X-Ray, microbiological sputum analysis, blood tests and ultrasound

Spirometry (1)

- An objective lung functioning test to measure airflow limitation
- With COPD and Asthma → both show obstruction (air is trapped inside lung vs. restriction (not enough air gets inside lung)):

➢ FEV1↓(Forced Expiratory Volume in 1 second)

➢ FEV1/FVC ratio↓(Forced Expiratory Volume in 1 second/Forced Vital Capacity ratio)

Spirometry (2)

→To distinguish Asthma and COPD a fast-acting bronchodilator (i.e. Salbutamol) is needed (Asthma has a reversible airflow obstruction)

→ If post-bronchodilator FEV1/FVC ratio is still < 0.7 (70%), then a COPD is likely

BUT: Spirometry is often not available in Zambia

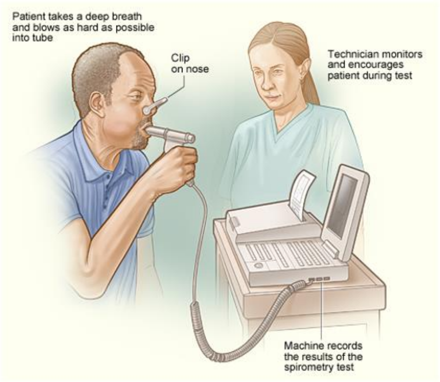

https://en.wikipedia.org/wiki/Spirometer

Spirometry curves

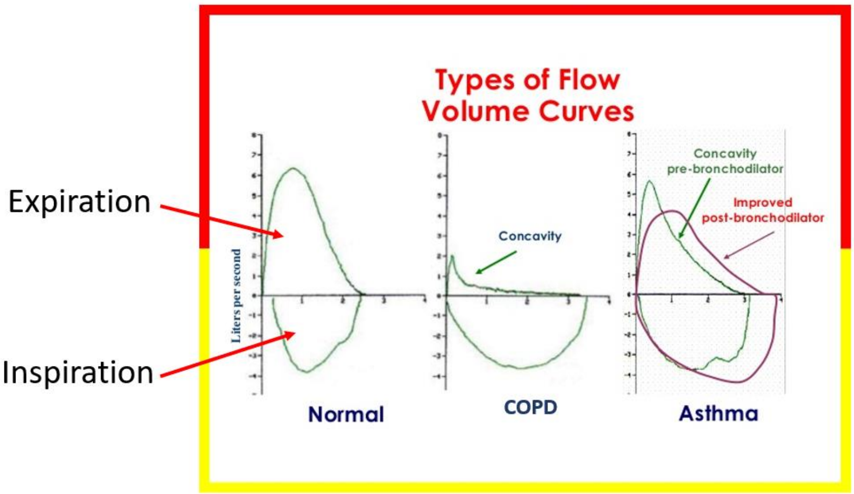

https://www.slideshare.net/ashrafeladawy/spirometry-basics-2

Spirometry curves

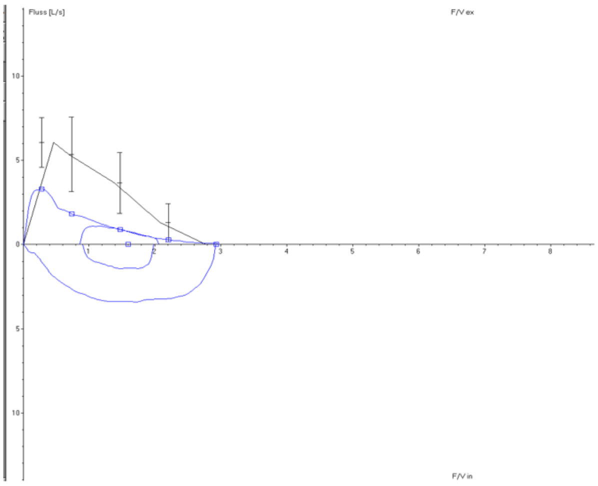

Real life flow-volume curve of a COPD patient, blue= COPD

Alternative diagnosis

- With Microspirometers (small, handheld spirometers), questionnaires or PEF devices (Peak Expiratory Flow)
- Examples:

➢ COPD-6 Microspirometer

➢ CDQ (COPD Diagnostic Questionnaire)

➢ CAPTURE Questionnaire in combination with PEF

CXR in COPD

- COPD typically causes hyperinflation of the lung and lung emphysema

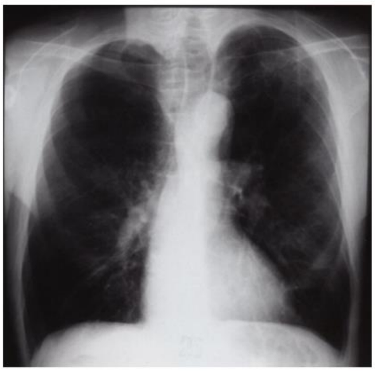

- Low diaphragm
- Barrel chest (round thorax)
- Increased radio transparency (darker lungs)
- Wide intercostal spaces
- Horizontal rips

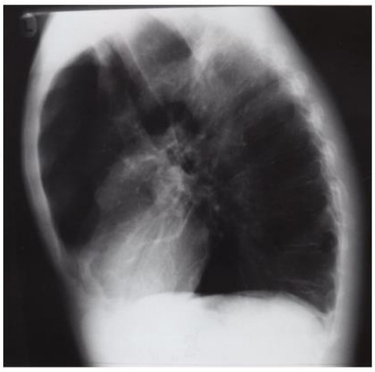

https://next.amboss.com/de/article/3h0Sd
f#Z0f05e84bc654d358a399384fdd42cdd0

Classification of severity by GOLD (Global Initiative of Obstructive Lung Disease)

<u>Severity of airflow limitation</u>:

- With spirometry

GOLD group	FEV1 (% of predicted)
1	≥80
2	50-79
3	30-49
4	<30

Severity of symptoms:

- CAT Questionnaire (COPD Assessment Test) → measures symptoms
  - Comprises 8 questions about cough, sputum production, chest tightness, dyspnea, limitation of physical activity, fear of leavingthe house, sleep, and energy; each to be recorded on a scale of 1 – 6
- mMRC Questionnaire (Modified British Medical Research Council) → measures dyspnea
  - 0 = dyspnea on strong exertion
  - 1 = dyspnea when climbing stairs
  - 2 = dyspnea when walking on even ground
  - 3 = dyspnea when walking on even ground, < 100 m
  - 4 = dyspnea when getting dressed / undressed

ABCD-scheme of GOLD classification:

- 2 parameters to determine group: symptoms + exacerbations in the last year (acute worsening of COPD symptoms over a short period of time for more than 24 hours)

Patient group	Exacerbations/year	mMRC	CAT
A	≤1	0-1	<10
B	≤1	≥2	≥10
C	≥2	0-1	<10
D	≥2	≥2	≥10

Differential diagnosis (1)

- Asthma: early onset in life, reversible airflow obstruction, symptoms vary daily, symptom-free intervals, allergic component → Bronchodilator test
- Tuberculosis: Chest X-Ray and microbiological confirmation
- Congestive Heart Failure: Dilated heart in chest X-Ray and pulmonary edema → restriction, not obstruction
- Bronchiectasis: High sputum production, chest X-Ray shows dilated bronchi and thick bronchial walls

Differential diagnosis (2)

- Bronchial carcinoma: Chest X-Ray
- Gastroesophageal reflux: can cause chronic cough
- Pulmonary embolism
- Vocal cord dysfunction

→ CODP is diagnosed by exclusion, bronchial carcinoma and Tuberculosis need to be ruled out

Therapy

Non-Pharmacological therapy:

- Smoking cessation
- Vaccinations: Pneumococcal vaccine and Influenza vaccine
- Pulmonary rehabilitation (education, respiratory exercises, physical activity, self-management inventions)
- Osteoporosis prophylaxis: Vitamin D3 and Calcium; lifestyle of patients but also treatment with corticosteroids can increase risk of osteoporosis
- Oxygen therapy: If paO2 is <55 mmHg or <60 mmHg with right heart failure

Pharmacological therapy:

- Bronchodilators:

➢Beta-2 Agonists: Short acting (SABA) or long acting (LABA)

➢Anticholinergics: Short acting (SAMA) or long acting (LAMA)

→ Short acting bronchodilators for acute relief and long acting bronchodilators for long-term therapy

Corticosteroids:

➢ICS→ only in combination with LABA

- Roflumilast: Selective PDE4-inhibitor with strong anti-inflammatory effect, indicated in severe COPD III/IV
- Theophyllin: because of low efficiency and relevant adverse effects / interactions only used as reserve

Group	Therapy
A	Bronchodilators (short or long acting)
B	LAMA or LABA, if not enough: LAMA+LABA
C	LAMA, with continuous exacerbations: LAMA+LABA or LABA+ICS
D	LAMA+LABA<br>With continuous exacerbations: LAMA+LABA+ICS<br>Possibly Roflumilast

Prognosis (1)

- 30% of COPD patients are diagnosed when already in GOLD stages 3 or 4
- Important: early diagnosis! → for patients in GOLD stages 3 and 4 life expectancy is significantly reduced
- COPD can be treated and its progression can be slowed when diagnosed and treated early

Prognosis (2)

<u>Risk factors for an unfavourable progression:</u>

- ➢Many exacerbations
- ➢High age
- ➢Hypercapnia
- ➢Long-term therapy with corticosteroids
- ➢Other severe comorbidity (heart failure…)

Sources

- Gold Report 2020
- Amboss, Chapter „COPD“
- Herold, Gerd: Innere Medizin, Köln. 2019., pages 348-354

Thank you for participating in this e-learning module! Now, to finish, we would like you to take a knowledge test and fill out a user satisfaction survey. You can find the instructions in the email you received.
