# Supplementary material for: Real-life Evaluation of an Interactive Versus Noninteractive e-Learning Module on Chronic Obstructive Pulmonary Disease for Medical Licentiate Students in Zambia: Web-Based, Mixed Methods Randomized Controlled Trial
Source: JMIR Med Educ. 2022 Feb 24;8(1):e34751. doi: 10.2196/34751 (PMC8914755; doi:10.2196/34751)
Supplement: Multimedia Appendix 3 [file mededu_v8i1e34751_app3.pdf]

Screenshots interactive module

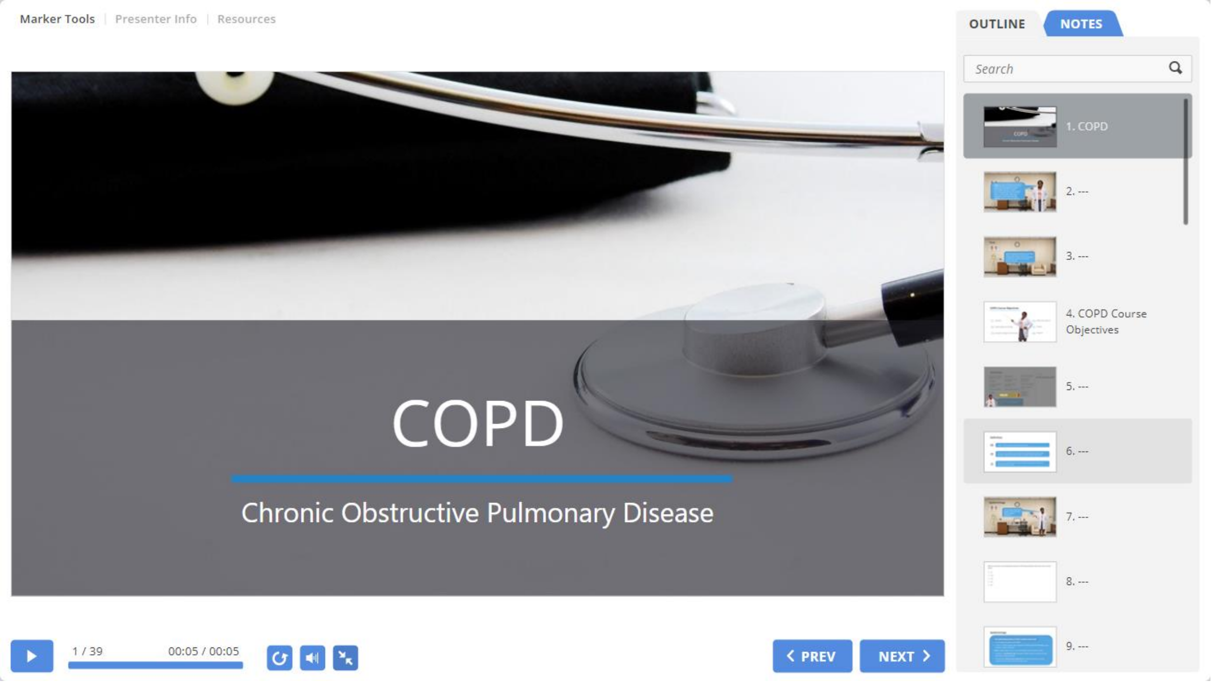

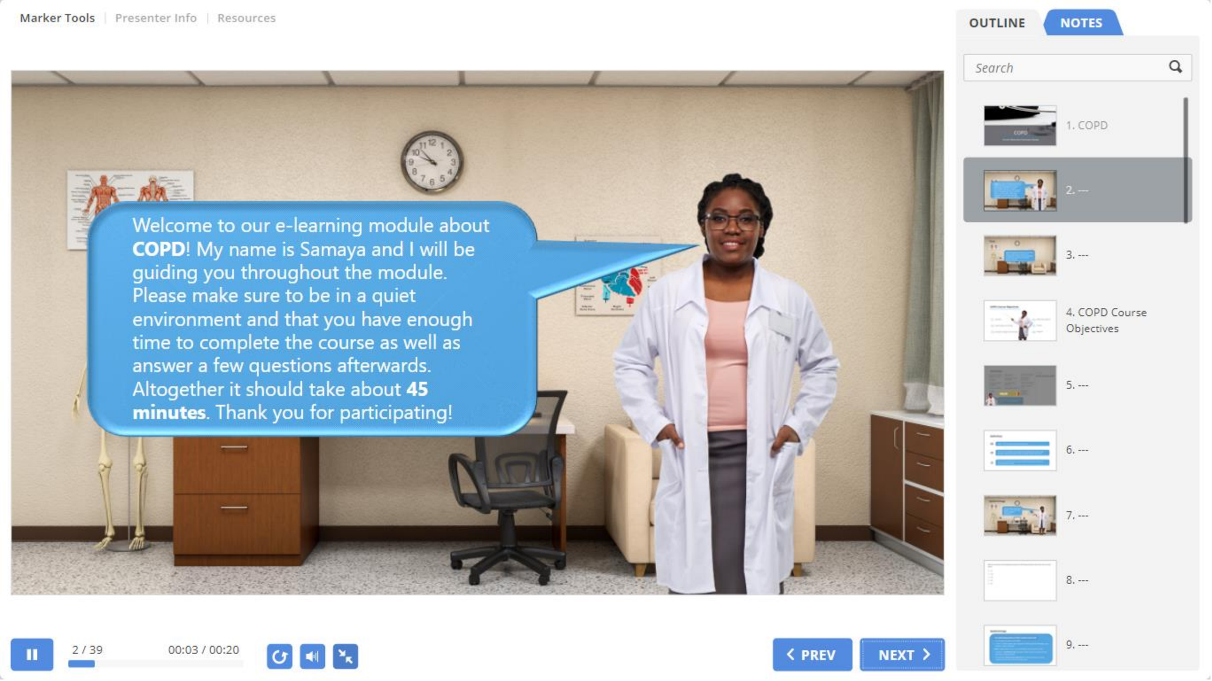

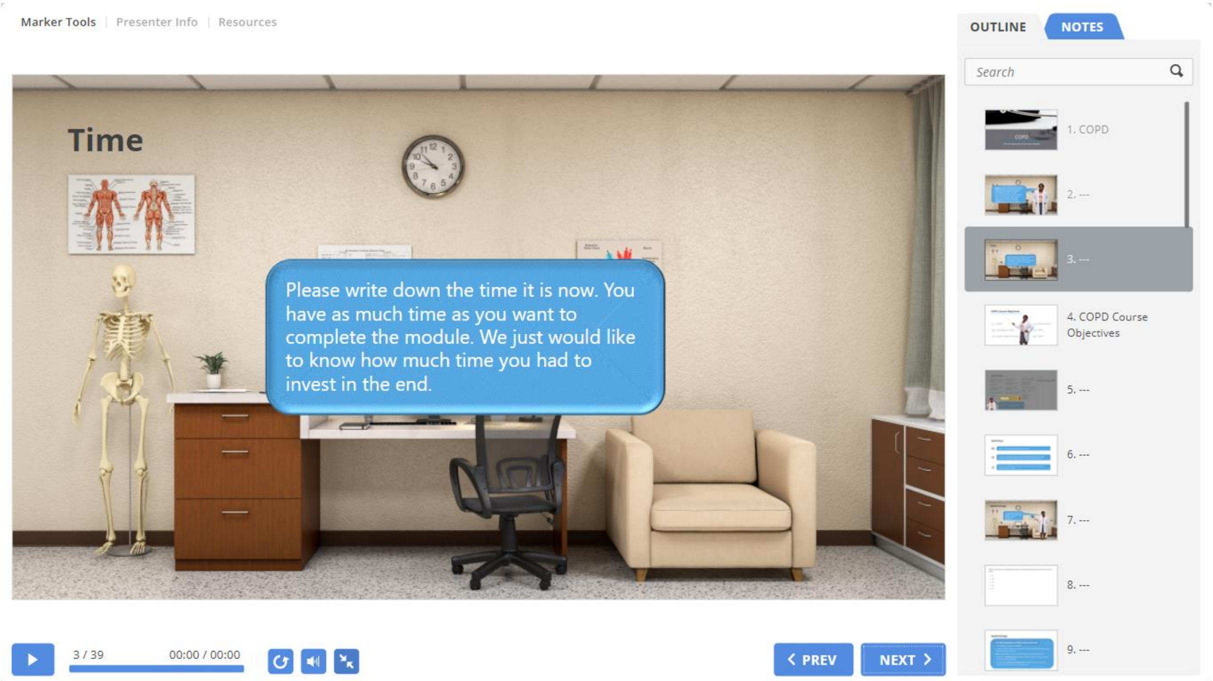
Marker Tools
Presenter Info
Resources
Time
Please write down the time it is now. You have as much time as you want to complete the module. We just would like to know how much time you had to invest in the end.
3 / 39
00:00 / 00:00
PREV
NEXT
OUTLINE
NOTES
Search
1. COPD
2. ---
3. ---
4. COPD Course Objectives
5. ---
6. ---
7. ---
8. ---
9. ---

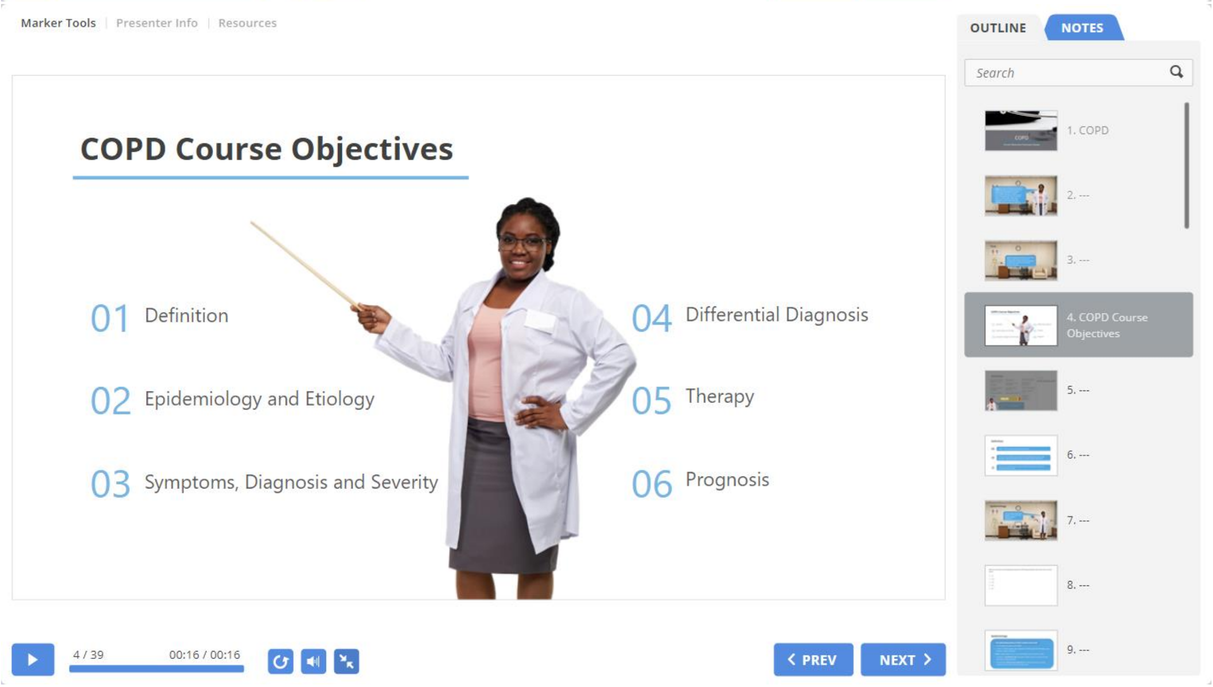
Marker Tools
Presenter Info
Resources
COPD Course Objectives
01 Definition
02 Epidemiology and Etiology
03 Symptoms, Diagnosis and Severity
04 Differential Diagnosis
05 Therapy
06 Prognosis
4 / 39
00:16 / 00:16
PREV
NEXT
OUTLINE
NOTES
Search
1. COPD
2. ---
3. ---
4. COPD Course Objectives
5. ---
6. ---
7. ---
8. ---
9. ---

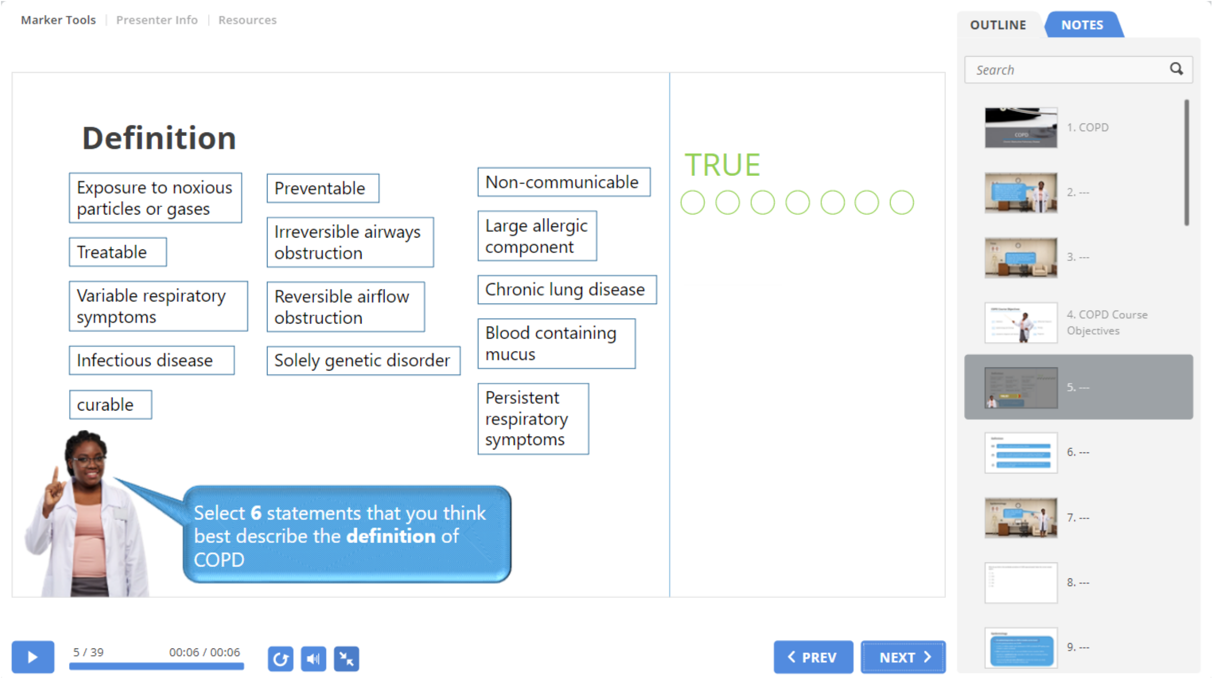

Marker Tools | Presenter Info | Resources
Definition
Exposure to noxious particles or gases
Treatable
Variable respiratory symptoms
Infectious disease
curable
Preventable
Irreversible airways obstruction
Reversible airflow obstruction
Solely genetic disorder
Non-communicable
Large allergic component
Chronic lung disease
Blood containing mucus
Persistent respiratory symptoms
Select 6 statements that you think best describe the definition of COPD
TRUE
5 / 39
00:06 / 00:06
PREV
NEXT
OUTLINE
NOTES
Search
1. COPD
2. ---
3. ---
4. COPD Course Objectives
5. ---
6. ---
7. ---
8. ---
9. ---

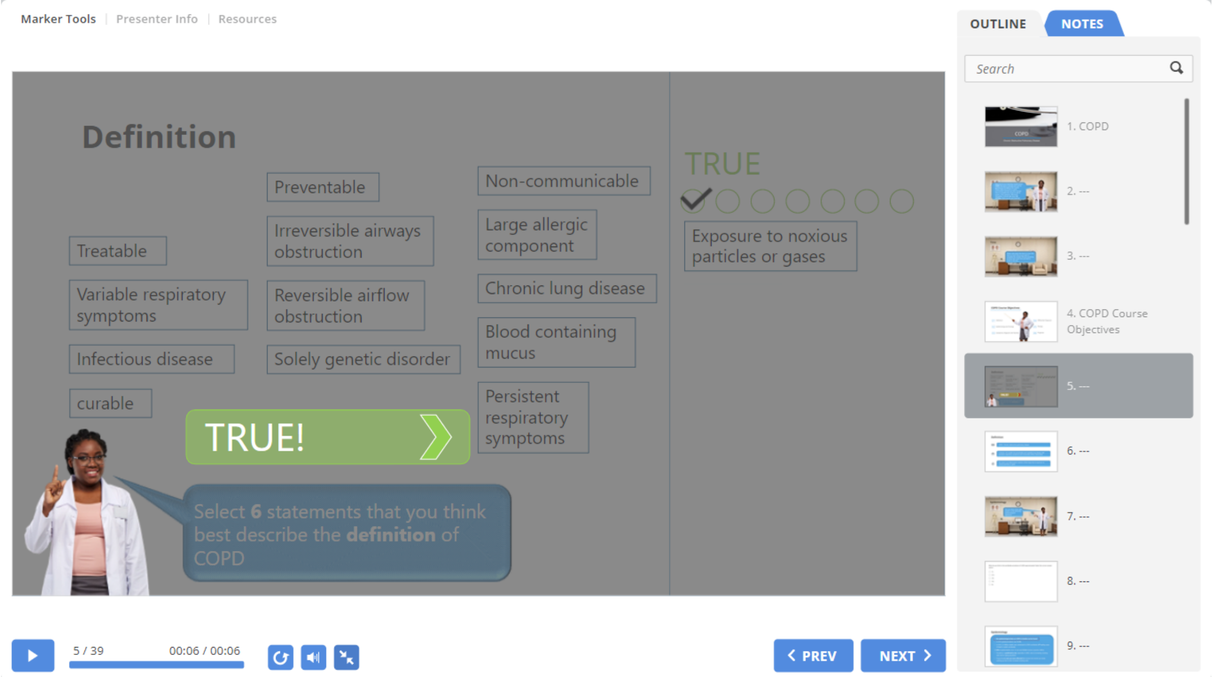

Marker Tools | Presenter Info | Resources
Definition
Treatable
Variable respiratory symptoms
Infectious disease
curable
Preventable
Irreversible airways obstruction
Reversible airflow obstruction
Solely genetic disorder
Non-communicable
Large allergic component
Chronic lung disease
Blood containing mucus
Persistent respiratory symptoms
TRUE!
Select 6 statements that you think best describe the definition of COPD
TRUE
Exposure to noxious particles or gases
5 / 39
00:06 / 00:06
PREV
NEXT
OUTLINE
NOTES
Search
1. COPD
2. ---
3. ---
4. COPD Course Objectives
5. ---
6. ---
7. ---
8. ---
9. ---

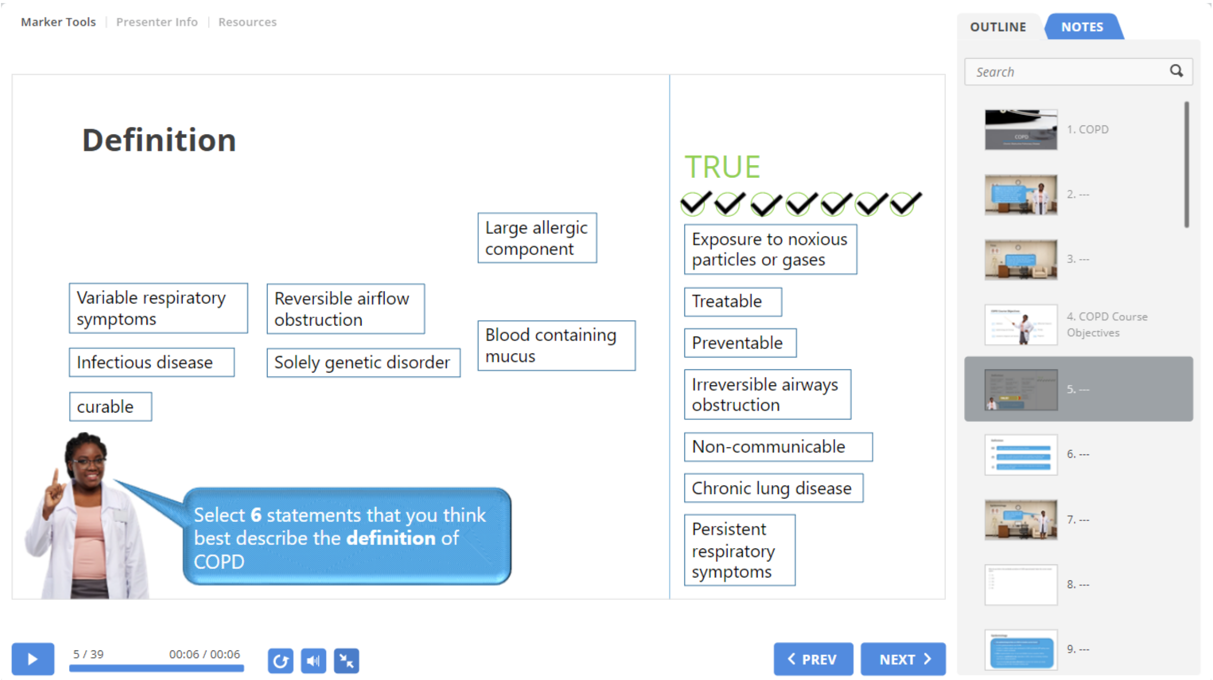

Marker Tools
Presenter Info
Resources
Definition
Large allergic component
Variable respiratory symptoms
Reversible airflow obstruction
Blood containing mucus
Infectious disease
Solely genetic disorder
curable
Select 6 statements that you think best describe the definition of COPD
TRUE
Exposure to noxious particles or gases
Treatable
Preventable
Irreversible airways obstruction
Non-communicable
Chronic lung disease
Persistent respiratory symptoms
5 / 39
00:06 / 00:06
PREV
NEXT
OUTLINE
NOTES
Search
1. COPD
2. ---
3. ---
4. COPD Course Objectives
5. ---
6. ---
7. ---
8. ---
9. ---

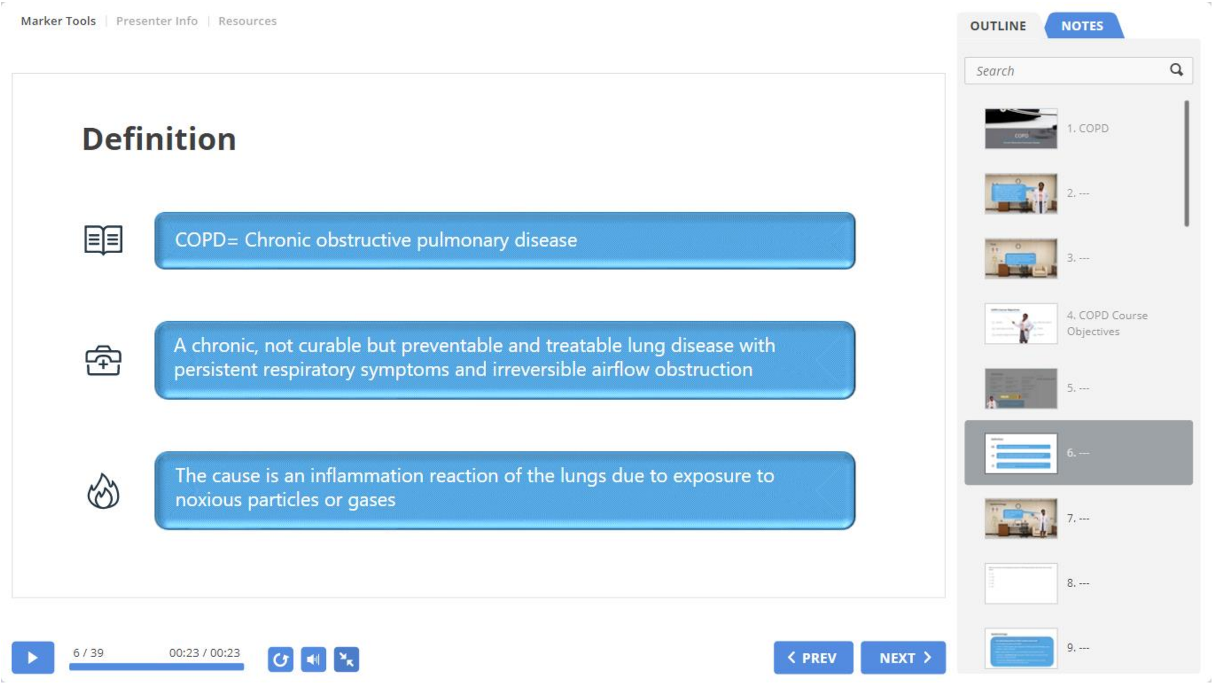

Marker Tools
Presenter Info
Resources
Definition
COPD= Chronic obstructive pulmonary disease
A chronic, not curable but preventable and treatable lung disease with persistent respiratory symptoms and irreversible airflow obstruction
The cause is an inflammation reaction of the lungs due to exposure to noxious particles or gases
6 / 39
00:23 / 00:23
PREV
NEXT
OUTLINE
NOTES
Search
1. COPD
2. ---
3. ---
4. COPD Course Objectives
5. ---
6. ---
7. ---
8. ---
9. ---

Marker Tools | Presenter Info | Resources

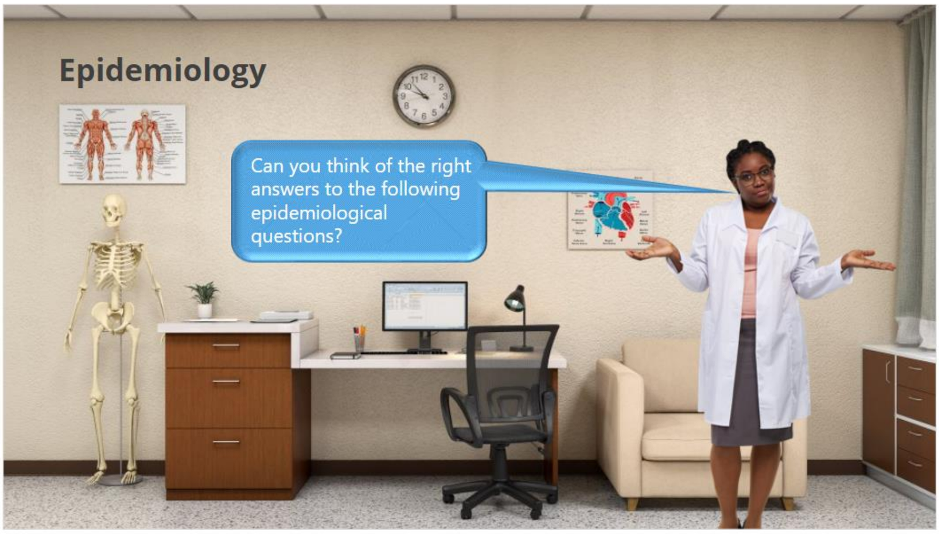

7 / 39 00:02 / 00:07

PREV NEXT

OUTLINE NOTES

Search

1. COPD
2. ---
3. ---
4. COPD Course Objectives
5. ---
6. ---
7. ---
8. ---
9. ---

Resources | Question 1 of 3 | Your Score: 0 of 30

What do you think is the worldwide prevalence of COPD approximately? Select the correct answer option:

- 0,5%
- 15%
- 5%
- 1%
- 10%

SUBMIT

OUTLINE NOTES

Search

1. COPD
2. ---
3. ---
4. COPD Course Objectives
5. ---
6. ---
7. ---
8. ---
8.1. What do you think is the worldwide preva...

Resources | Question 1 of 3 | Your Score: 0 of 30

What do you think is the worldwide prevalence of COPD approximately? Select the correct answer option:

- ○ 0,5%
- ○ 15%
- ○ 5%
- ○ 1%
- ◉ 10%

SUBMIT

OUTLINE | NOTES

Search

1. COPD
2. ---
3. ---
4. COPD Course Objectives
5. ---
6. ---
7. ---
8. ---
8.1. What do you think is the worldwide preva...

Resources | Question 1 of 3 | Your Score: 10 of 30

What do you think is the worldwide prevalence of COPD approximately? Select the correct answer option:

- ○ 0,5%
- ○ 15%
- ○ 5%
- ○ 1%
- ◉ 10%

Correct

That's right! You chose the correct response.

CONTINUE >

OUTLINE | NOTES

Search

1. COPD
2. ---
3. ---
4. COPD Course Objectives
5. ---
6. ---
7. ---
8. ---
8.1. What do you think is the worldwide preva...

Resources | Question 2 of 3 | Your Score: 20 of 30

Where does COPD rank in the list of overall causes of death in adults worldwide? Select the correct answer option:

- (•) 3rd
- ( ) 15th
- ( ) 10th
- ( ) 1st
- ( ) 30th

Correct

That's right! You chose the correct response.

CONTINUE

OUTLINE NOTES

Search

2. ---
3. ---
4. COPD Course Objectives
5. ---
6. ---
7. ---
8. ---
8.1. What do you think is the worldwide preva...
8.2. Where does COPD rank in the list of overall ca...

Resources | Question 3 of 3 | Your Score: 30 of 30

What percentage of those deaths occur in Low-and Middle-Income countries?

- (•) 90%
- ( ) 10%
- ( ) 70%
- ( ) 50%
- ( ) 30%

Correct

That's right! You chose the correct response.

VIEW RESULTS

OUTLINE NOTES

Search

3. ---
4. COPD Course Objectives
5. ---
6. ---
7. ---
8. ---
8.1. What do you think is the worldwide preva...
8.2. Where does COPD rank in the list of overall ca...
8.3. What percentage of those deaths oc...

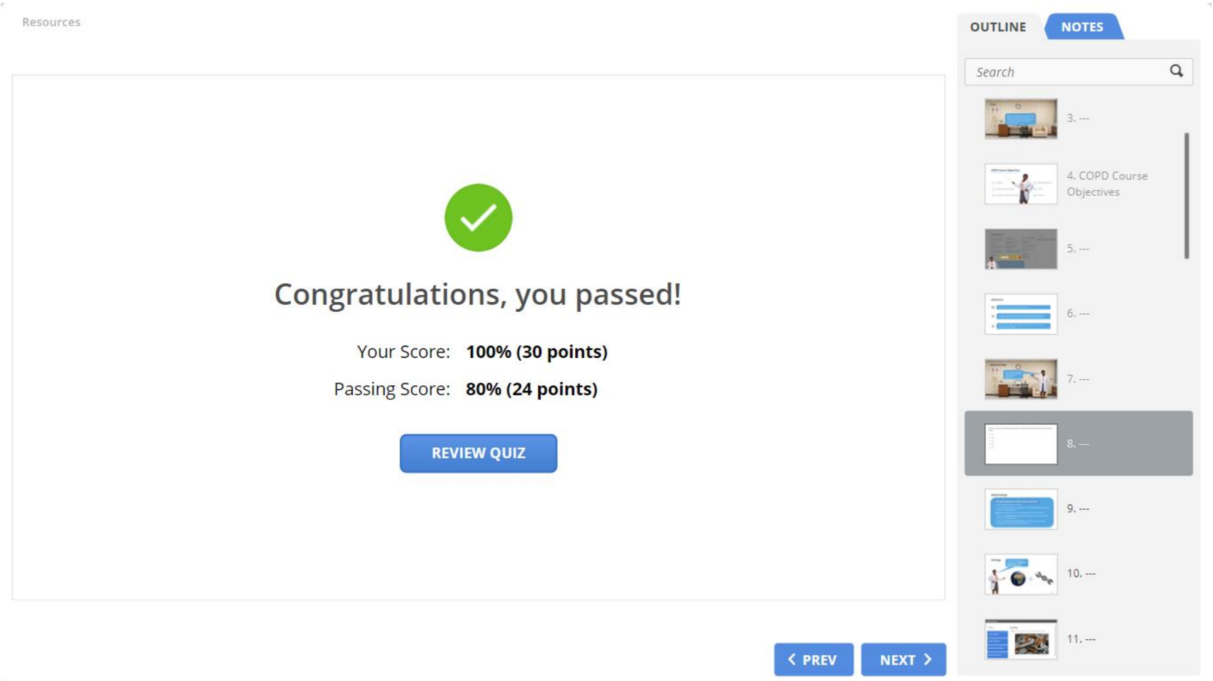
Resources
OUTLINE
NOTES
Search
3. ---
4. COPD Course Objectives
5. ---
6. ---
7. ---
8. ---
9. ---
10. ---
11. ---
Congratulations, you passed!
Your Score: 100% (30 points)
Passing Score: 80% (24 points)
REVIEW QUIZ
PREV
NEXT

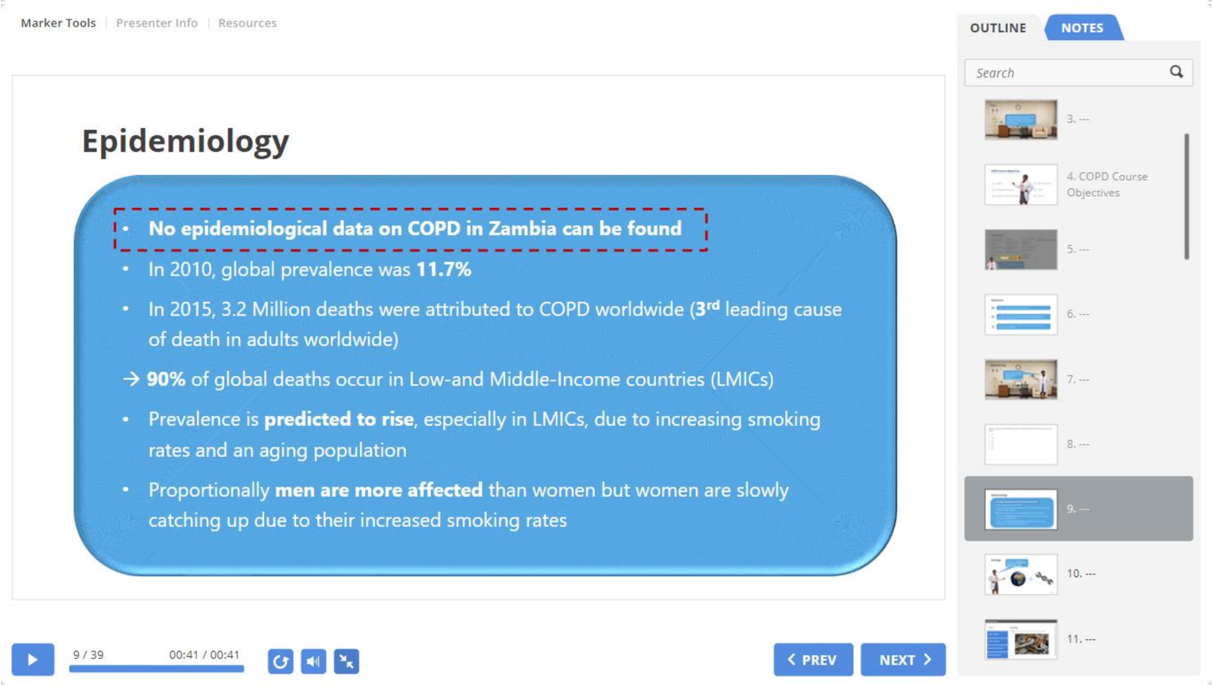
Marker Tools
Presenter Info
Resources
OUTLINE
NOTES
Search
Epidemiology
No epidemiological data on COPD in Zambia can be found
In 2010, global prevalence was 11.7%
In 2015, 3.2 Million deaths were attributed to COPD worldwide (3rd leading cause of death in adults worldwide)
→ 90% of global deaths occur in Low-and Middle-Income countries (LMICs)
Prevalence is predicted to rise, especially in LMICs, due to increasing smoking rates and an aging population
Proportionally men are more affected than women but women are slowly catching up due to their increased smoking rates
3. ---
4. COPD Course Objectives
5. ---
6. ---
7. ---
8. ---
9. ---
10. ---
11. ---
9 / 39
00:41 / 00:41
PREV
NEXT

Marker Tools | Presenter Info | Resources

Etiology

The causes of COPD are a mix between **environmental (exogenous)** and **host factors (endogenous)**

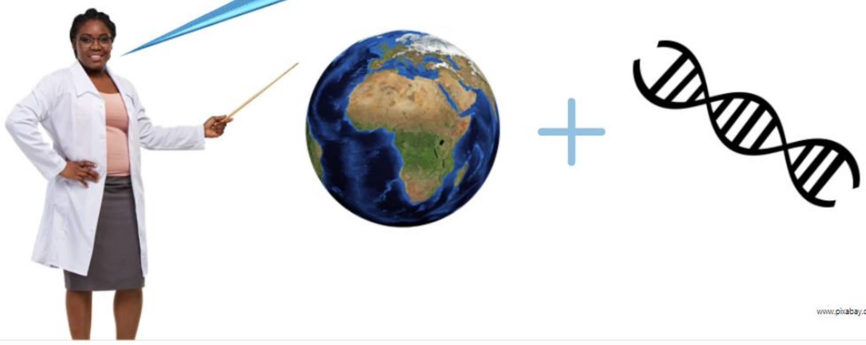

www.pixabay.com

10 / 39 00:03 / 00:07

PREV NEXT

OUTLINE NOTES

Search

3. ---
4. COPD Course Objectives
5. ---
6. ---
7. ---
8. ---
9. ---
10. ---
11. ---

Marker Tools | Presenter Info | Resources

Exogenous factors

- Smoking
- Indoor air pollution
- Outdoor air pollution
- Abnormal lung development
- Abnormal lung function

Smoking

In **90%** of cases, smoking is the cause of COPD in adults worldwide.

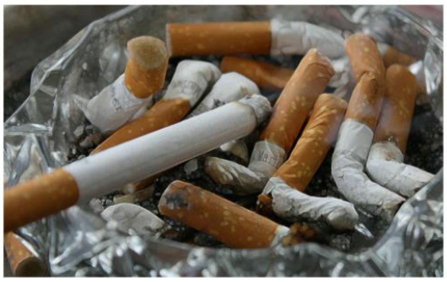

www.pixabay.com

PREV NEXT

OUTLINE NOTES

Search

3. ---
4. COPD Course Objectives
5. ---
6. ---
7. ---
8. ---
9. ---
10. ---
11. ---

Marker Tools | Presenter Info | Resources

OUTLINE NOTES

Search

3. ---

4. COPD Course Objectives

5. ---

6. ---

7. ---

8. ---

9. ---

10. ---

11. ---

Exogenous factors

- **Smoking**
- **Indoor air pollution**
- **Outdoor air pollution**
- **Abnormal lung development**
- **Abnormal lung function**

Indoor air pollution

The number of smokers in Subsaharan Africa are not high but lung diseases are very prevalent due to air pollution. Through **burning of biomass** (charcoal, dung, wood) in LMICs people with a lower socioeconomic status and women are more affected.

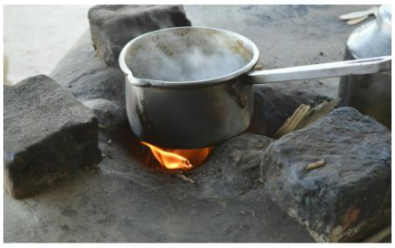

PREV NEXT

Marker Tools | Presenter Info | Resources

OUTLINE NOTES

Search

3. ---

4. COPD Course Objectives

5. ---

6. ---

7. ---

8. ---

9. ---

10. ---

11. ---

Exogenous factors

- **Smoking**
- **Indoor air pollution**
- **Outdoor air pollution**
- **Abnormal lung development**
- **Abnormal lung function**

Outdoor air pollution

Outdoor air pollution is a severe problem in LMICs.

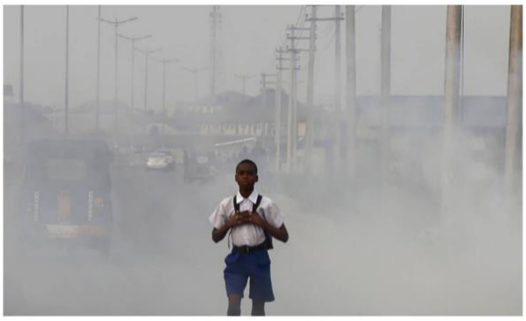

PREV NEXT

Marker Tools | Presenter Info | Resources

OUTLINE NOTES

Search

3. ---

4. COPD Course Objectives

5. ---

6. ---

7. ---

8. ---

9. ---

10. ---

11. ---

Exogenous factors

- Smoking
- Indoor air pollution
- Outdoor air pollution
- Abnormal lung development
- Abnormal lung function

Abnormal lung development

Due to **early childhood lung infections** or a **low birth weight**.

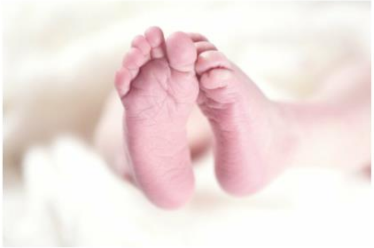

www.pixabay.com

PREV NEXT

Marker Tools | Presenter Info | Resources

OUTLINE NOTES

Search

3. ---

4. COPD Course Objectives

5. ---

6. ---

7. ---

8. ---

9. ---

10. ---

11. ---

Exogenous factors

- Smoking
- Indoor air pollution
- Outdoor air pollution
- Abnormal lung development
- Abnormal lung function

Abnormal lung function

Due to **Asthma**, a history of **Tuberculosis** or recurrent lower **respiratory tract infections**.

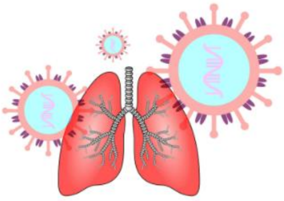

www.pixabay.com

PREV NEXT

Marker Tools | Presenter Info | Resources

Endogenous factors

Alpha 1 -Antitrypsin Defiency

Age

Alpha 1 -Antitrypsin Defiency

COPD patients that are **under 50 years** old should be tested for this genetic abnormality.

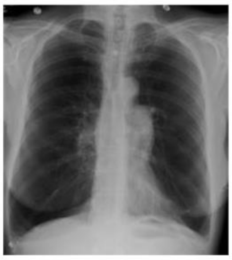

https://en.wikipedia.org/wiki/Alpha-1_antitrypsin_deficiency

OUTLINE NOTES

Search

4. COPD Course Objectives
5. ---
6. ---
7. ---
8. ---
9. ---
10. ---
11. ---
12. ---

PREV NEXT

Marker Tools | Presenter Info | Resources

Endogenous factors

Alpha 1 -Antitrypsin Defiency

Age

Age

COPD prevalence increases with age.

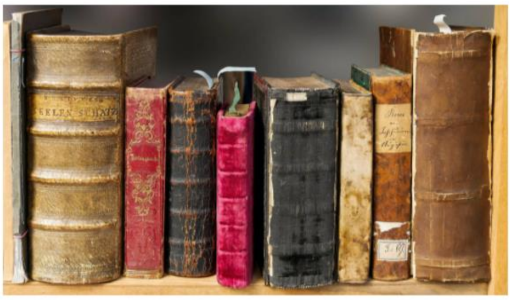

[illegible]

OUTLINE NOTES

Search

4. COPD Course Objectives
5. ---
6. ---
7. ---
8. ---
9. ---
10. ---
11. ---
12. ---

PREV NEXT

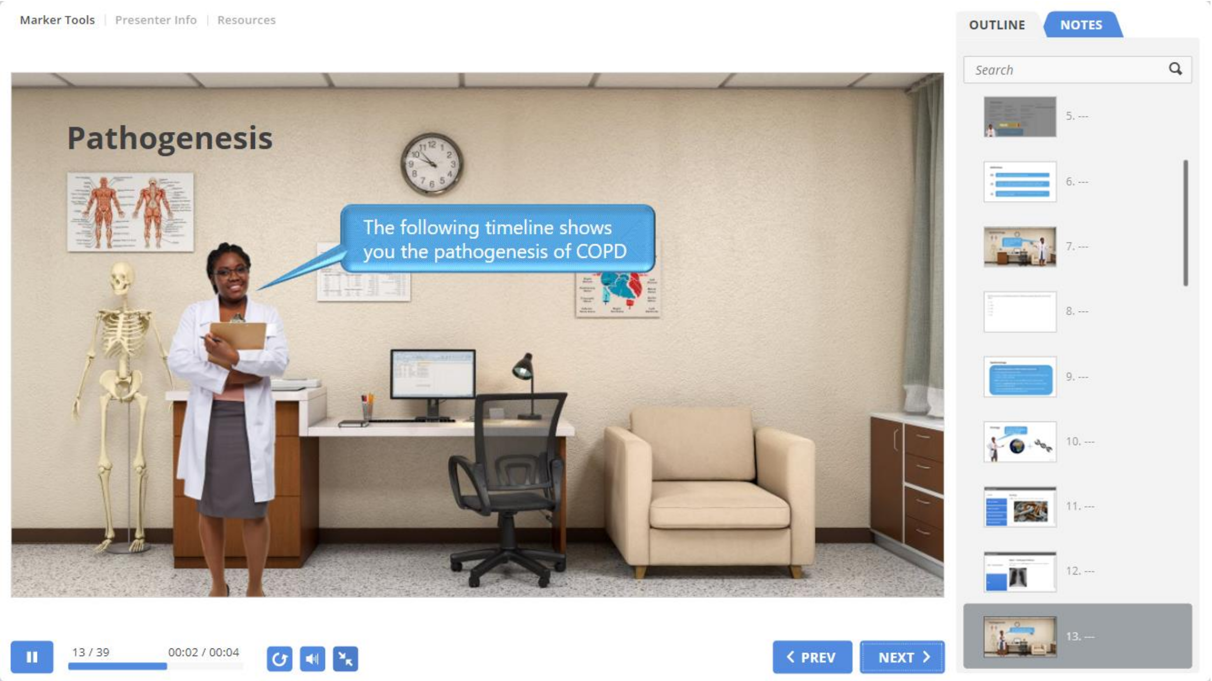

Marker Tools | Presenter Info | Resources
OUTLINE NOTES
Search
Pathogenesis
The following timeline shows you the pathogenesis of COPD
5. ---
6. ---
7. ---
8. ---
9. ---
10. ---
11. ---
12. ---
13. ---
13 / 39
00:02 / 00:04
PREV
NEXT

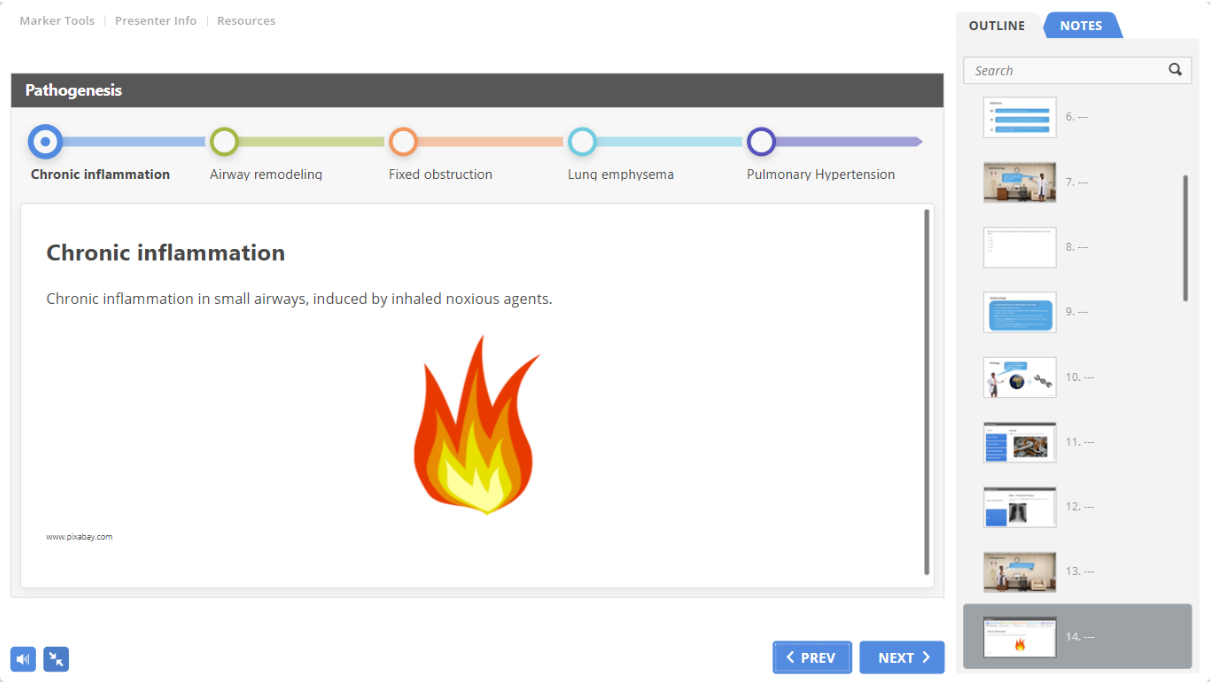

Marker Tools | Presenter Info | Resources
OUTLINE NOTES
Search
Pathogenesis
Chronic inflammation
Airway remodeling
Fixed obstruction
Lung emphysema
Pulmonary Hypertension
Chronic inflammation
Chronic inflammation in small airways, induced by inhaled noxious agents.
www.pixabay.com
6. ---
7. ---
8. ---
9. ---
10. ---
11. ---
12. ---
13. ---
14. ---
PREV
NEXT

Marker Tools | Presenter Info | Resources

OUTLINE NOTES

Search

6. ---
7. ---
8. ---
9. ---
10. ---
11. ---
12. ---
13. ---
14. ---

Pathogenesis

Chronic inflammation | **Airway remodeling** | Fixed obstruction | Lung emphysema | Pulmonary Hypertension

Airway remodeling

Fibrosis and loss of parenchyma, bronchial instability and hyper secretion of mucus.

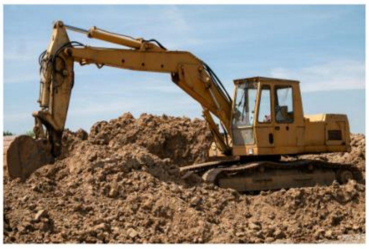

PREV NEXT

Marker Tools | Presenter Info | Resources

OUTLINE NOTES

Search

6. ---
7. ---
8. ---
9. ---
10. ---
11. ---
12. ---
13. ---
14. ---

Pathogenesis

Chronic inflammation | Airway remodeling | **Fixed obstruction** | Lung emphysema | Pulmonary Hypertension

Fixed obstruction

Obstruction with collapse of bronchioles/bronchi during forced expiration.

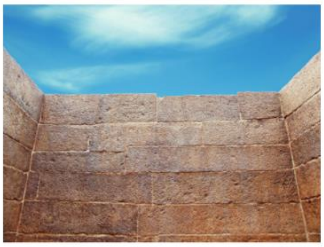

PREV NEXT

Marker Tools | Presenter Info | Resources

OUTLINE NOTES

Search

6. ---
7. ---
8. ---
9. ---
10. ---
11. ---
12. ---
13. ---
14. ---

Pathogenesis

Chronic inflammation · Airway remodeling · Fixed obstruction · **Lung emphysema** · Pulmonary Hypertension

Lung emphysema

Hyperinflation of the lung with reduced ventilation.

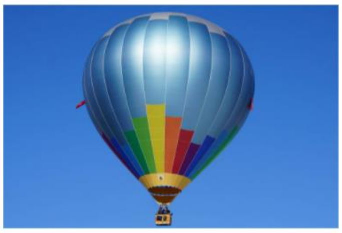

PREV NEXT

Marker Tools | Presenter Info | Resources

OUTLINE NOTES

Search

6. ---
7. ---
8. ---
9. ---
10. ---
11. ---
12. ---
13. ---
14. ---

Pathogenesis

Chronic inflammation · Airway remodeling · Fixed obstruction · Lung emphysema · **Pulmonary Hypertension**

Pulmonary Hypertension

Hypoxic pulmonary vasoconstriction leads to pulmonary hypertension and a chronic cor pulmonale.

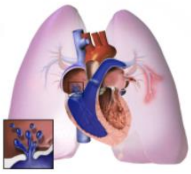

https://en.wikipedia.org/wiki/Pulmonary_hypertension

PREV NEXT

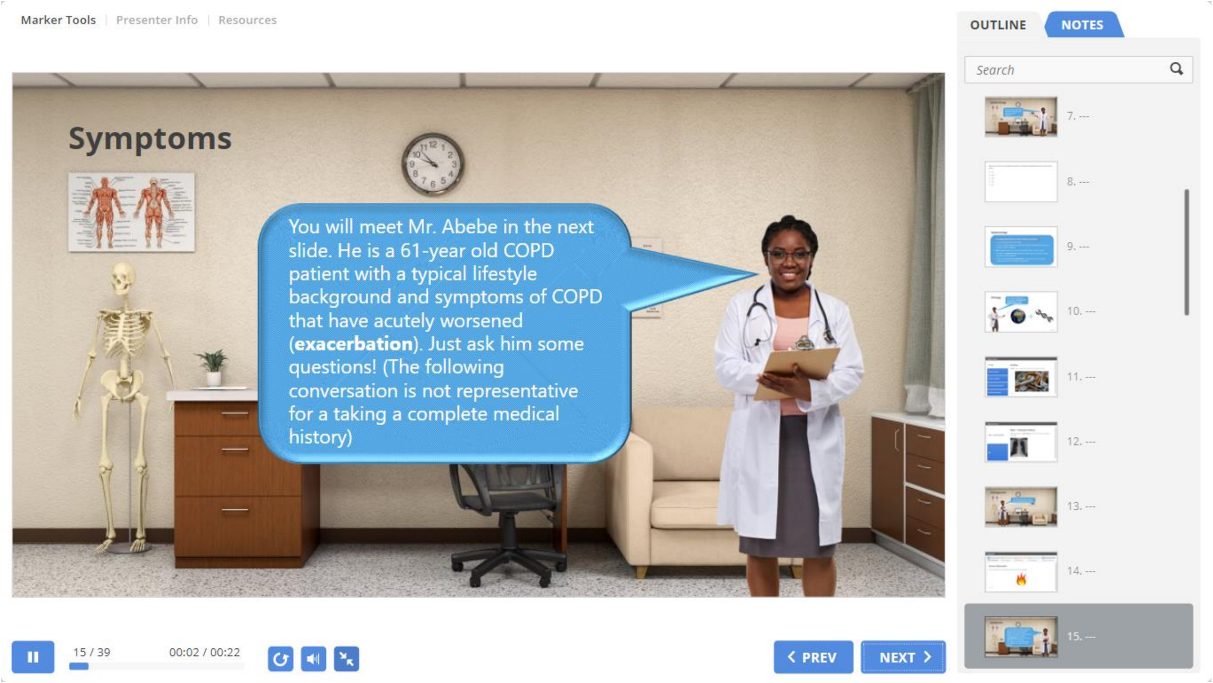
Marker Tools
Presenter Info
Resources
Symptoms
You will meet Mr. Abebe in the next slide. He is a 61-year old COPD patient with a typical lifestyle background and symptoms of COPD that have acutely worsened (**exacerbation**). Just ask him some questions! (The following conversation is not representative for a taking a complete medical history)
15 / 39
00:02 / 00:22
PREV
NEXT
OUTLINE
NOTES
Search
7. ---
8. ---
9. ---
10. ---
11. ---
12. ---
13. ---
14. ---
15. ---

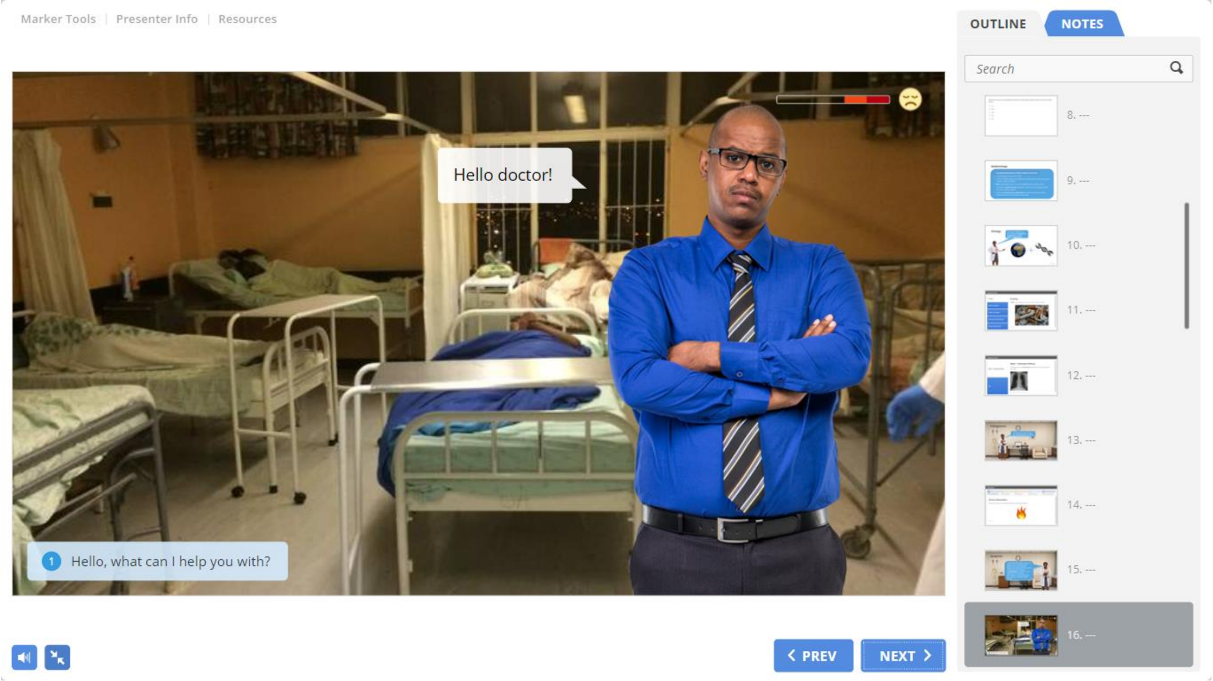
Marker Tools
Presenter Info
Resources
Hello doctor!
1 Hello, what can I help you with?
PREV
NEXT
OUTLINE
NOTES
Search
8. ---
9. ---
10. ---
11. ---
12. ---
13. ---
14. ---
15. ---
16. ---

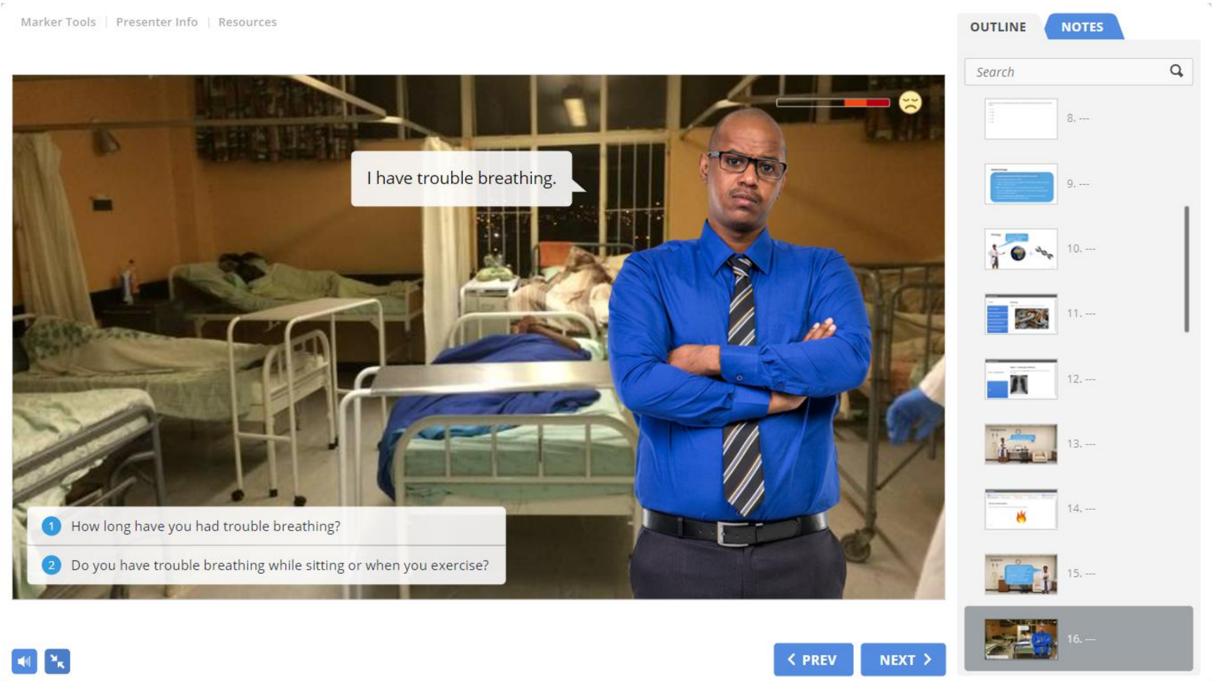
Marker Tools | Presenter Info | Resources
OUTLINE
NOTES
Search
8. ---
9. ---
10. ---
11. ---
12. ---
13. ---
14. ---
15. ---
16. ---
I have trouble breathing.
1 How long have you had trouble breathing?
2 Do you have trouble breathing while sitting or when you exercise?
PREV
NEXT

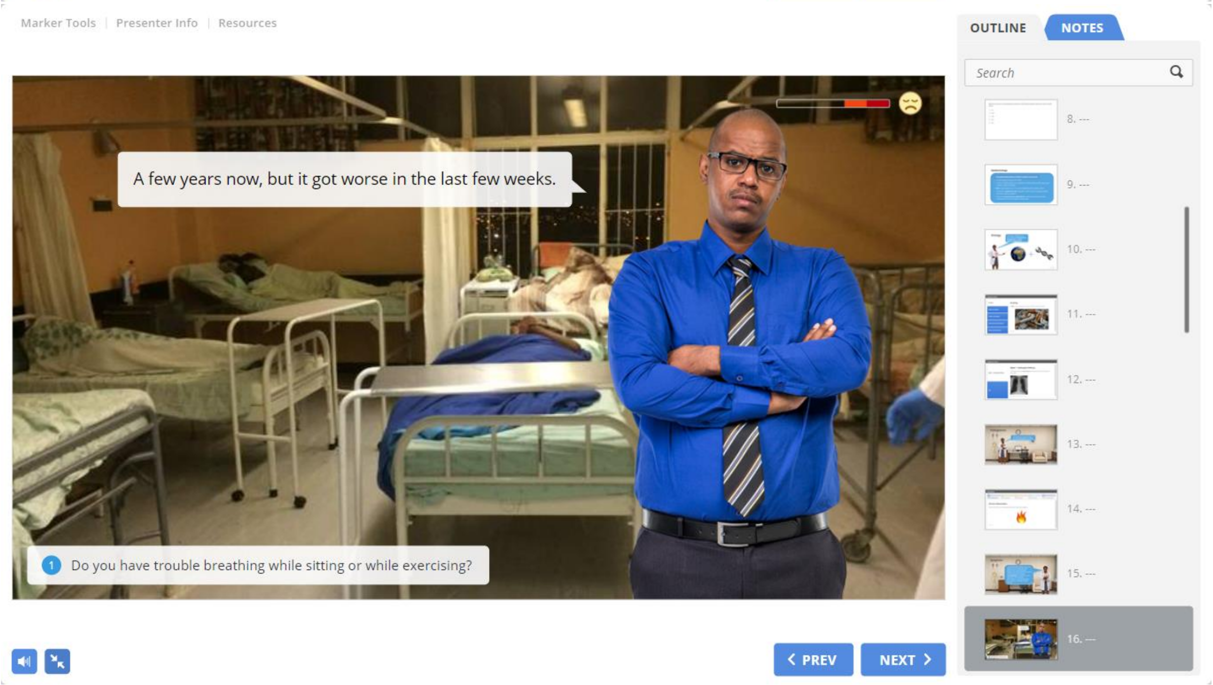
Marker Tools | Presenter Info | Resources
OUTLINE
NOTES
Search
8. ---
9. ---
10. ---
11. ---
12. ---
13. ---
14. ---
15. ---
16. ---
A few years now, but it got worse in the last few weeks.
1 Do you have trouble breathing while sitting or while exercising?
PREV
NEXT

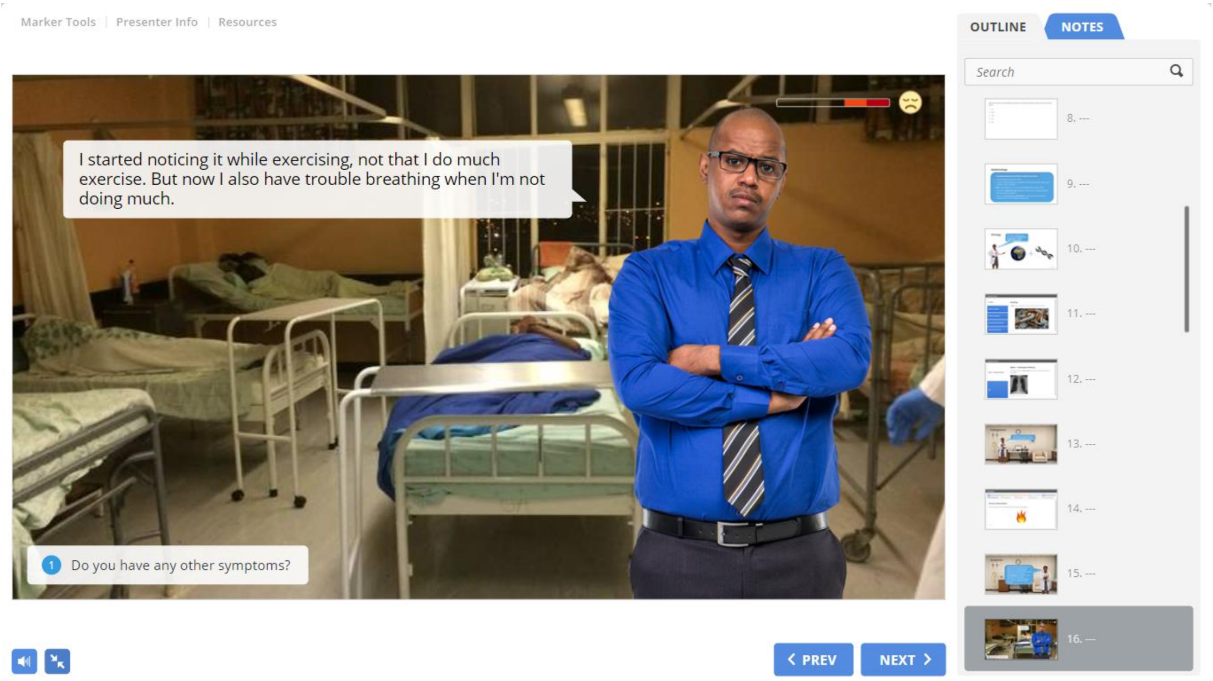
Marker Tools | Presenter Info | Resources
OUTLINE
NOTES
Search
I started noticing it while exercising, not that I do much exercise. But now I also have trouble breathing when I'm not doing much.
1 Do you have any other symptoms?
8. ---
9. ---
10. ---
11. ---
12. ---
13. ---
14. ---
15. ---
16. ---
PREV
NEXT

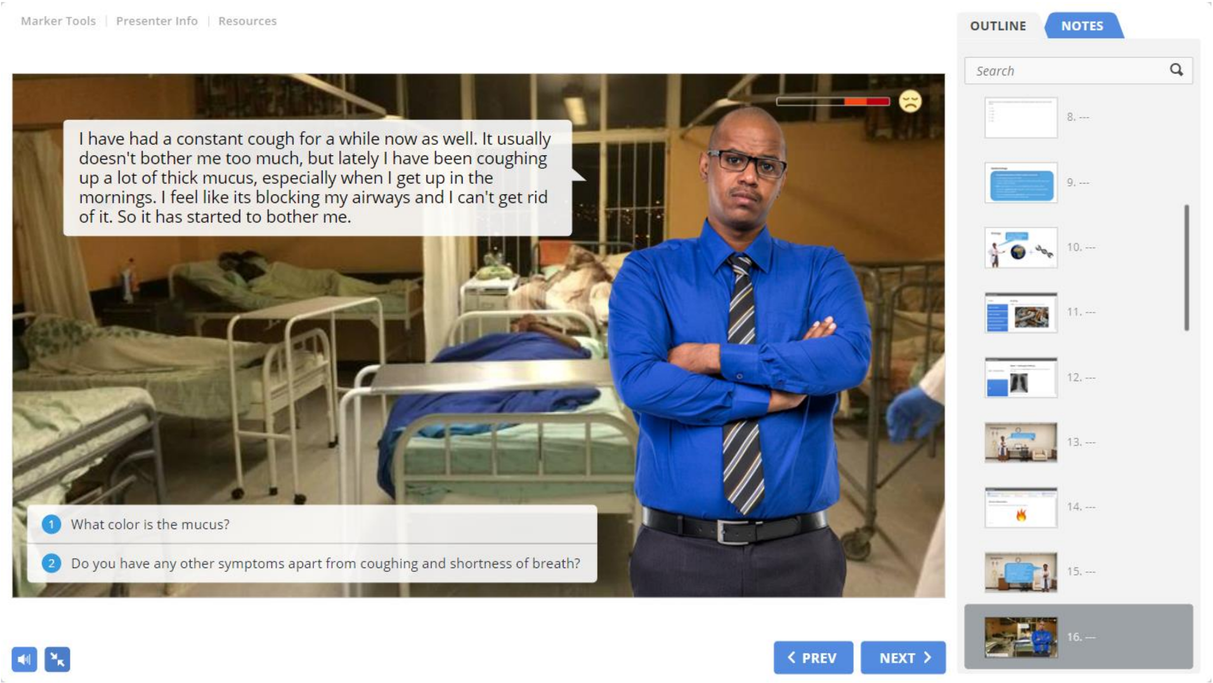
Marker Tools | Presenter Info | Resources
OUTLINE
NOTES
Search
I have had a constant cough for a while now as well. It usually doesn't bother me too much, but lately I have been coughing up a lot of thick mucus, especially when I get up in the mornings. I feel like its blocking my airways and I can't get rid of it. So it has started to bother me.
1 What color is the mucus?
2 Do you have any other symptoms apart from coughing and shortness of breath?
8. ---
9. ---
10. ---
11. ---
12. ---
13. ---
14. ---
15. ---
16. ---
PREV
NEXT

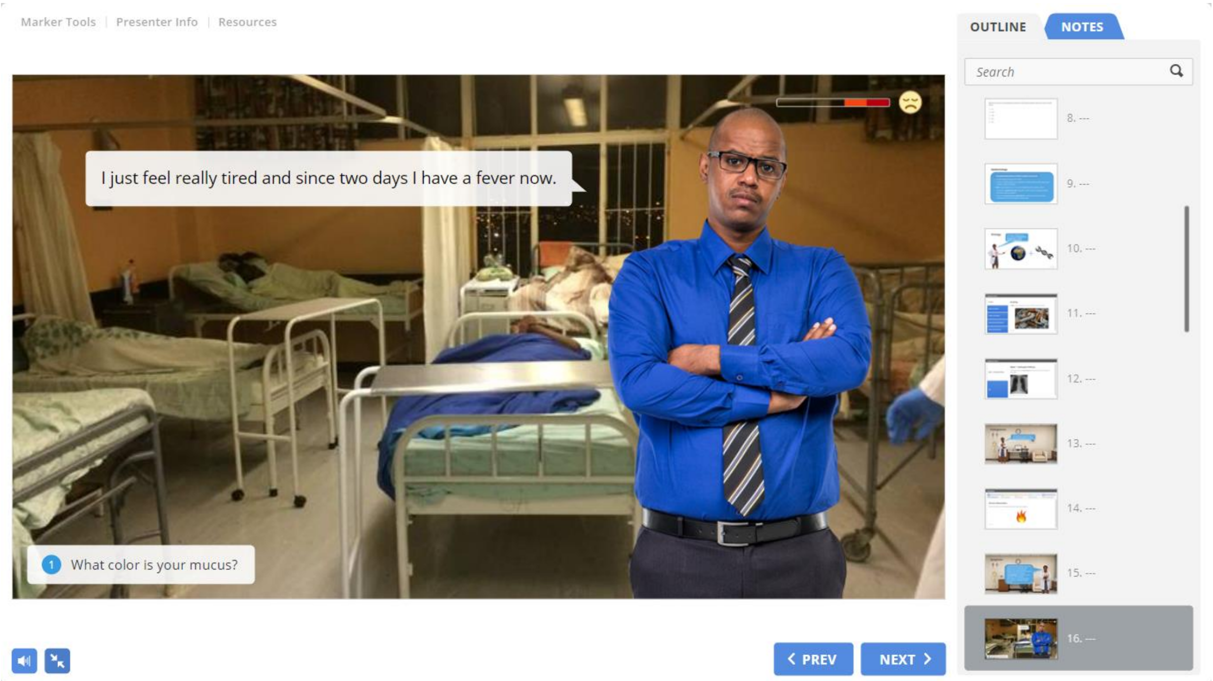
Marker Tools
Presenter Info
Resources
I just feel really tired and since two days I have a fever now.
1 What color is your mucus?
PREV
NEXT
OUTLINE
NOTES
Search
8. ---
9. ---
10. ---
11. ---
12. ---
13. ---
14. ---
15. ---
16. ---

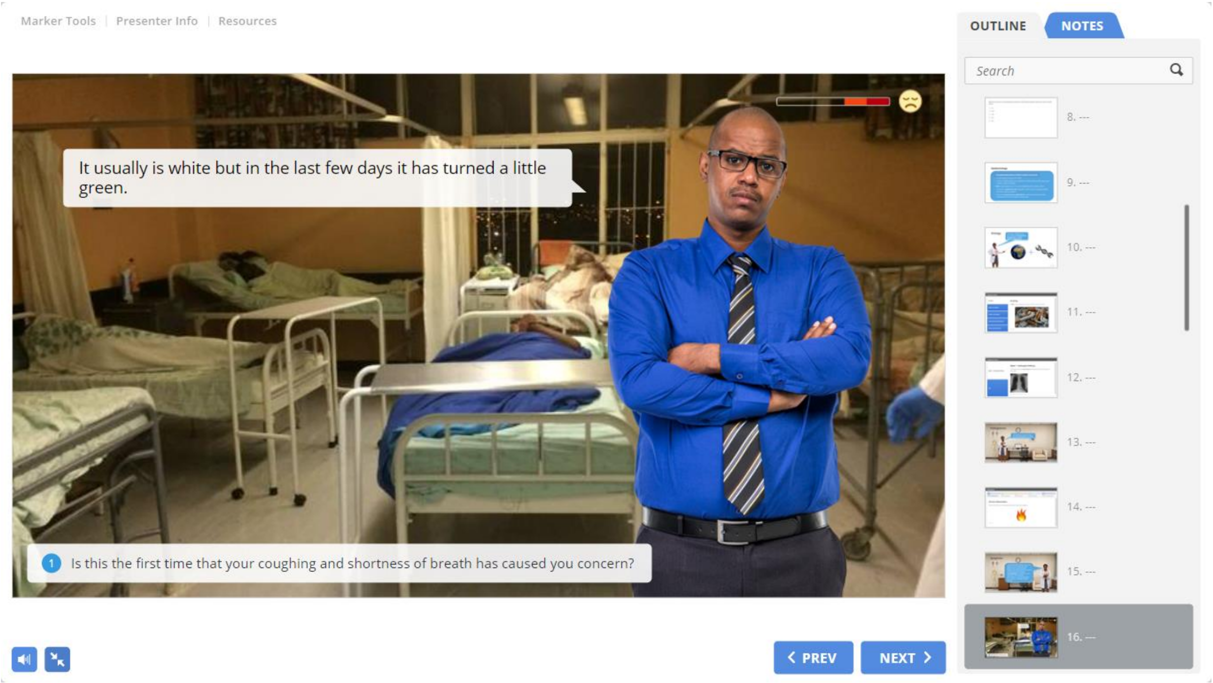
Marker Tools
Presenter Info
Resources
It usually is white but in the last few days it has turned a little green.
1 Is this the first time that your coughing and shortness of breath has caused you concern?
PREV
NEXT
OUTLINE
NOTES
Search
8. ---
9. ---
10. ---
11. ---
12. ---
13. ---
14. ---
15. ---
16. ---

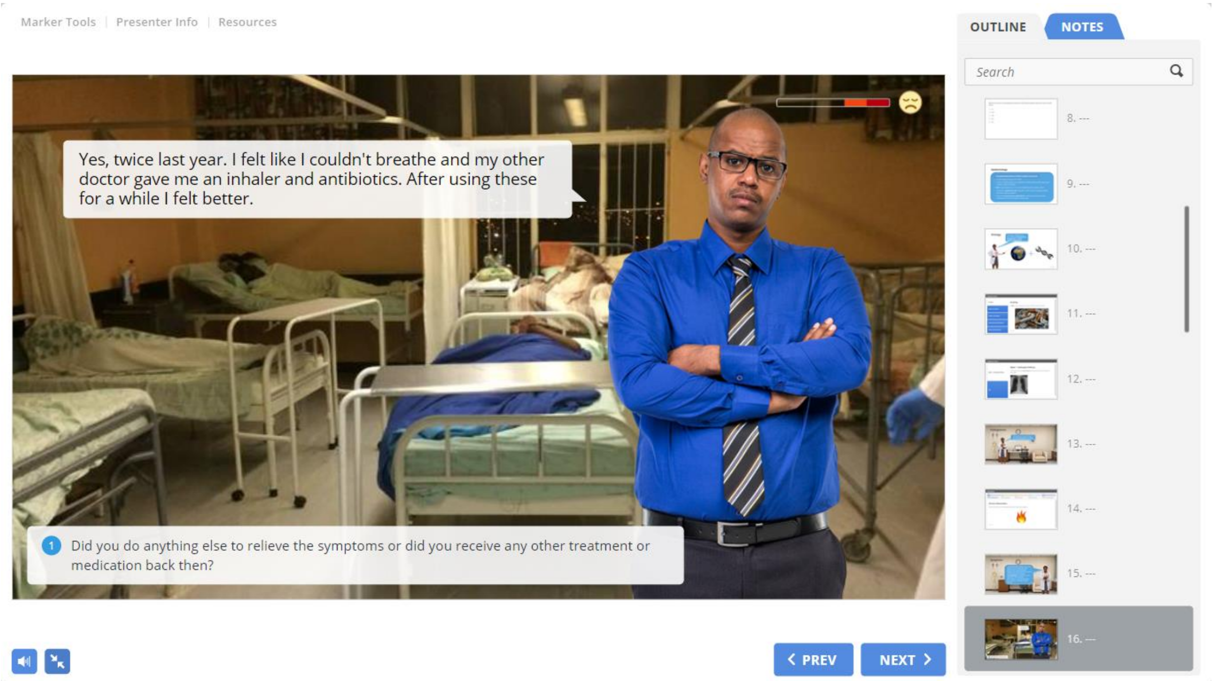
Marker Tools
Presenter Info
Resources
OUTLINE
NOTES
Search
8. ---
9. ---
10. ---
11. ---
12. ---
13. ---
14. ---
15. ---
16. ---
Yes, twice last year. I felt like I couldn't breathe and my other doctor gave me an inhaler and antibiotics. After using these for a while I felt better.
1 Did you do anything else to relieve the symptoms or did you receive any other treatment or medication back then?
PREV
NEXT

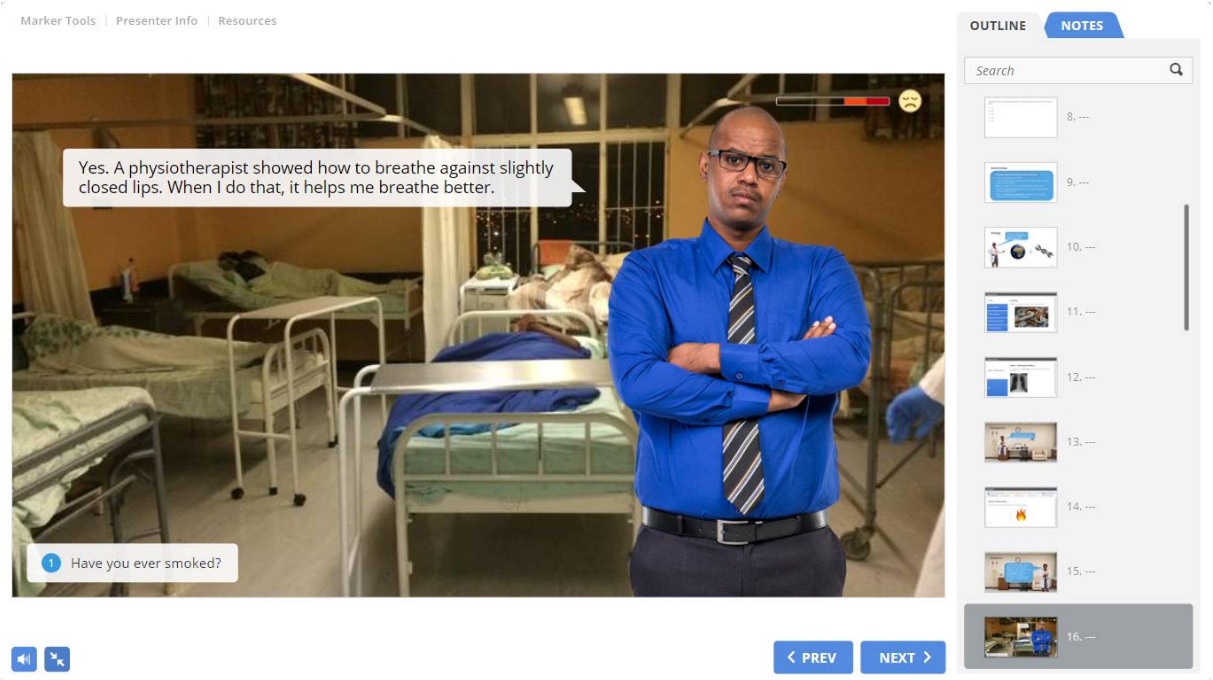
Marker Tools
Presenter Info
Resources
OUTLINE
NOTES
Search
8. ---
9. ---
10. ---
11. ---
12. ---
13. ---
14. ---
15. ---
16. ---
Yes. A physiotherapist showed how to breathe against slightly closed lips. When I do that, it helps me breathe better.
1 Have you ever smoked?
PREV
NEXT

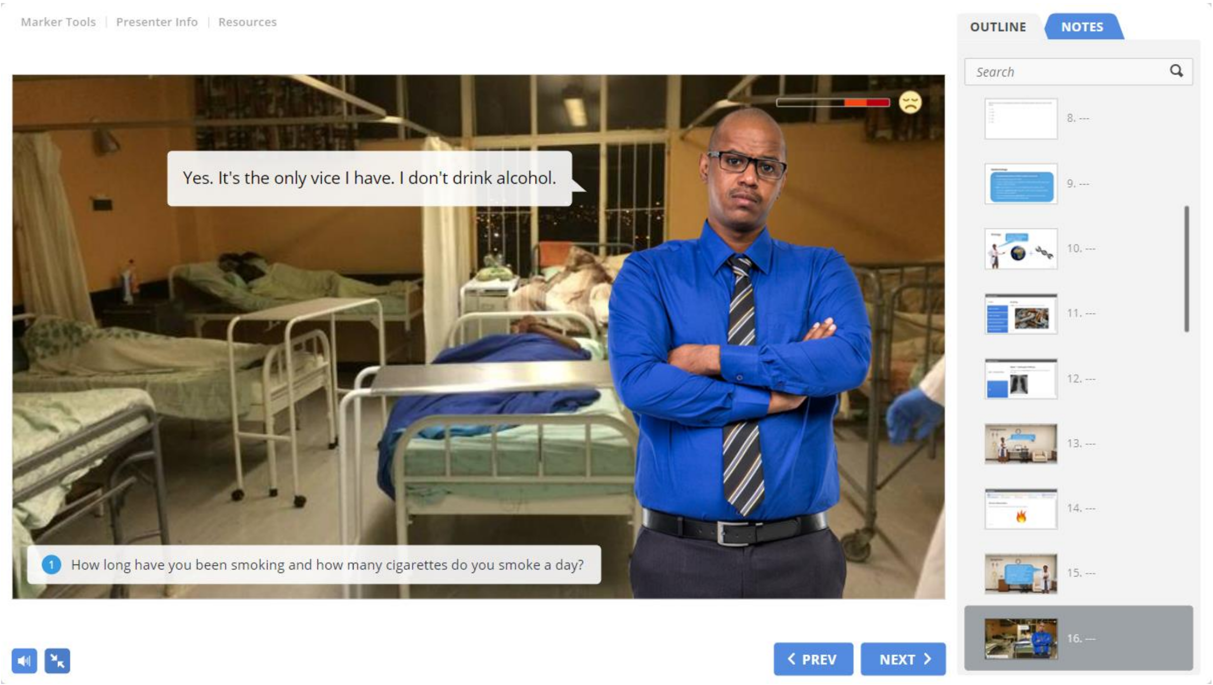
Marker Tools | Presenter Info | Resources
OUTLINE
NOTES
Search
Yes. It's the only vice I have. I don't drink alcohol.
1 How long have you been smoking and how many cigarettes do you smoke a day?
8. ---
9. ---
10. ---
11. ---
12. ---
13. ---
14. ---
15. ---
16. ---
PREV
NEXT

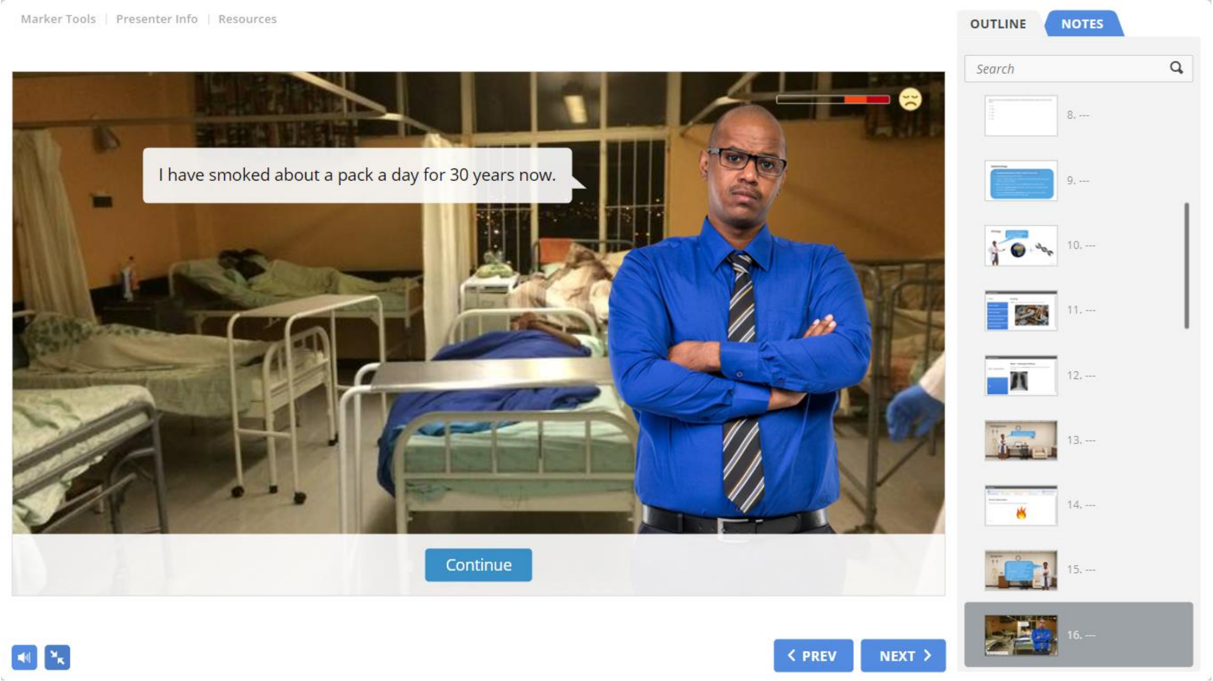
Marker Tools | Presenter Info | Resources
OUTLINE
NOTES
Search
I have smoked about a pack a day for 30 years now.
Continue
8. ---
9. ---
10. ---
11. ---
12. ---
13. ---
14. ---
15. ---
16. ---
PREV
NEXT

Resources | Question 1 of 1 | Your Score: 0 of 10

What are typical COPD symptoms? Drop the right symptoms on the lung. 5 symptoms are correct.

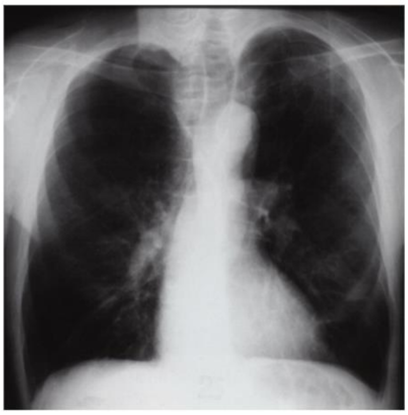

chronic cough

repeated lower respiratory tract infections

allergic reaction

high sputum production

recently developed cough

nausea

breathing with open mouth

no sputum production

cephalgia

dyspnea

angina pectoris

pursing of lips

SUBMIT

OUTLINE | NOTES

Search

10. ---

11. ---

12. ---

13. ---

14. ---

15. ---

16. ---

17. ---

17.1. What are typical COPD symptoms? Drop...

Resources | Question 1 of 1 | Your Score: 0 of 10

What are typical COPD symptoms? Drop the right symptoms on the lung. 5 symptoms are correct.

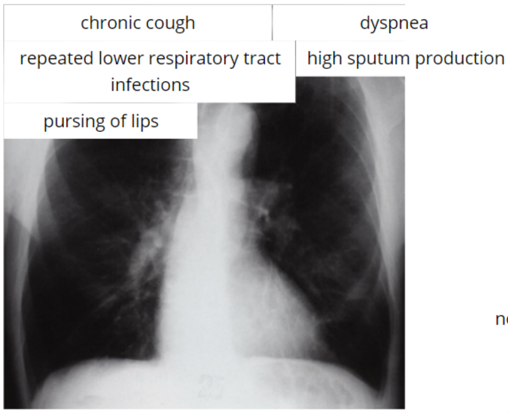

allergic reaction

recently developed cough

nausea

breathing with open mouth

no sputum production

cephalgia

angina pectoris

SUBMIT

OUTLINE | NOTES

Search

10. ---

11. ---

12. ---

13. ---

14. ---

15. ---

16. ---

17. ---

17.1. What are typical COPD symptoms? Drop...

Resources | Question 1 of 1 | Your Score: 10 of 10

What are typical COPD symptoms? Drop the right symptoms on the lung. 5 symptoms are correct.

chronic cough

dyspnea

repeated lower respiratory tract infections

high sputum production

pursing of lips

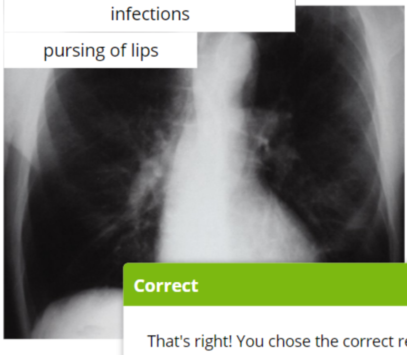

allergic reaction

recently developed cough

nausea

breathing with open mouth

no sputum production

cephalgia

Correct

That's right! You chose the correct response.

VIEW RESULTS

OUTLINE | NOTES

Search

10. ---

11. ---

12. ---

13. ---

14. ---

15. ---

16. ---

17. ---

17.1. What are typical COPD symptoms? Drop...

Resources

Congratulations, you passed!

Your Score: **100% (10 points)**

Passing Score: **80% (8 points)**

REVIEW QUIZ

PREV | NEXT

OUTLINE | NOTES

Search

10. ---

11. ---

12. ---

13. ---

14. ---

15. ---

16. ---

17. ---

18. ---

Marker Tools | Presenter Info | Resources

OUTLINE NOTES

Search

Summary of symptoms

- **Chronic cough** (may be purulent during infectious exacerbations)
- **High sputum production**
- **Dyspnea** (initially while exercising, later constantly)
- History of **repeated** lower respiratory tract **infections**
- **Pursing of lips**: during expiration patients close lips slightly → by breathing out with resistance air trapping in the lungs is prevented, maintaining an intrabronchial pressure that prevents collapse of small airways

10. ---
11. ---
12. ---
13. ---
14. ---
15. ---
16. ---
17. ---
18. ---

18 / 39 00:33 / 00:33

PREV NEXT

Marker Tools | Presenter Info | Resources

OUTLINE NOTES

Search

Diagnosis

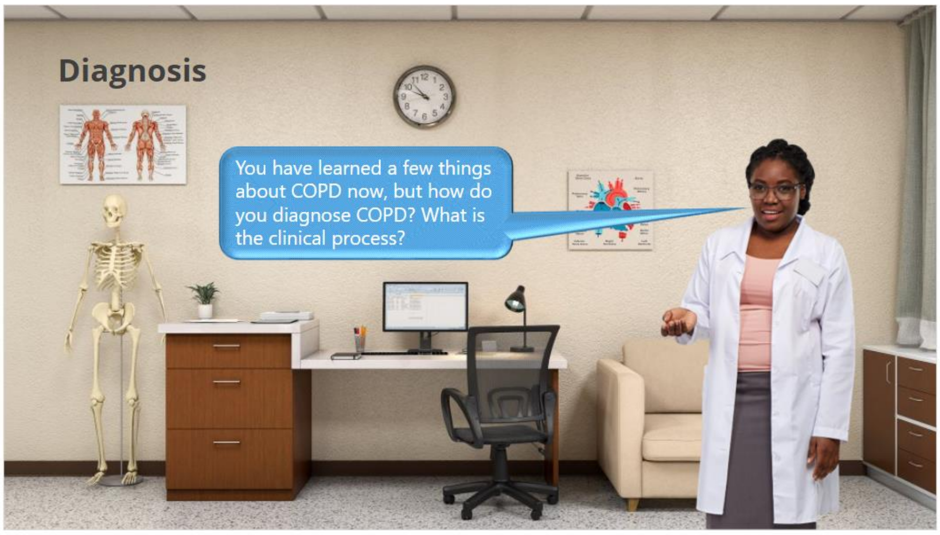

11. ---
12. ---
13. ---
14. ---
15. ---
16. ---
17. ---
18. ---
19. ---

19 / 39 00:02 / 00:08

PREV NEXT

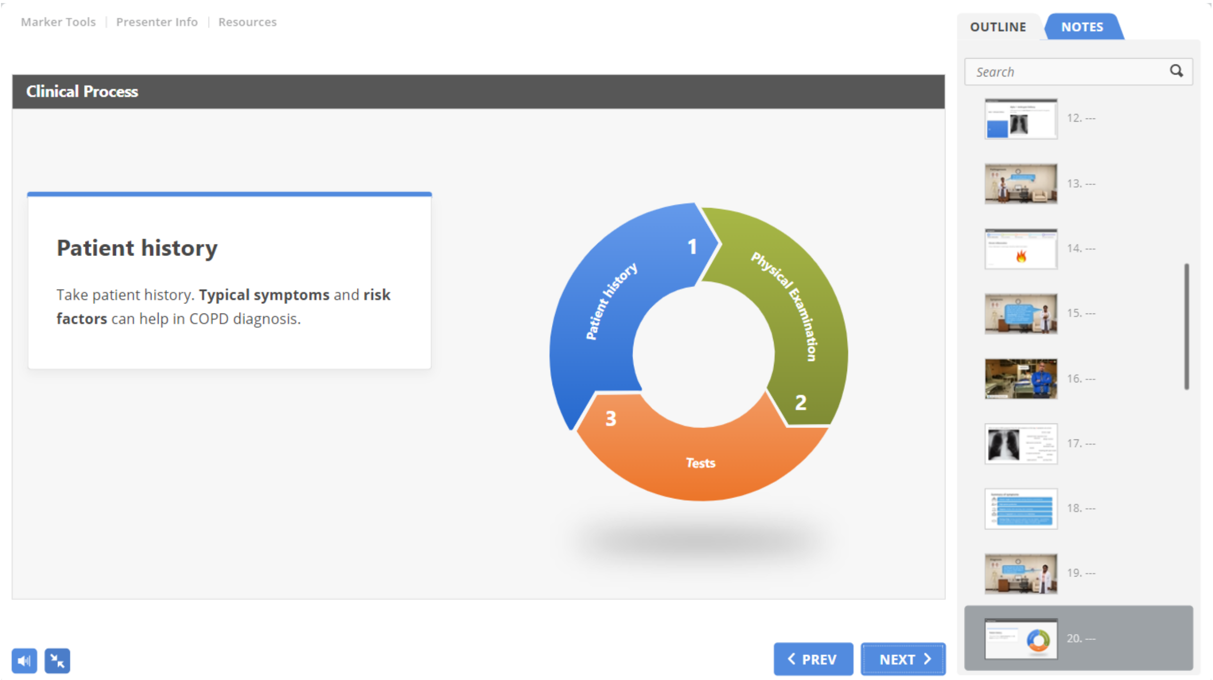
Marker Tools | Presenter Info | Resources
OUTLINE
NOTES
Search
Clinical Process
Patient history
Take patient history. **Typical symptoms** and **risk factors** can help in COPD diagnosis.
1
Patient history
Physical Examination
2
3
Tests
12. ---
13. ---
14. ---
15. ---
16. ---
17. ---
18. ---
19. ---
20. ---
PREV
NEXT

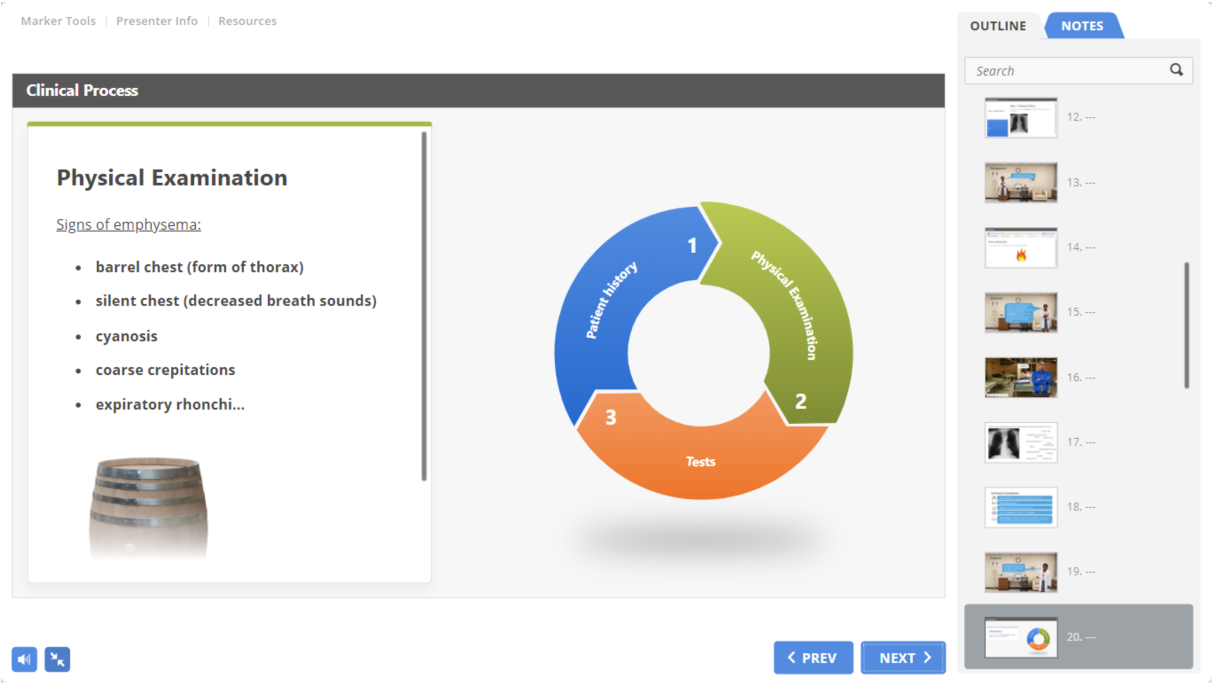
Marker Tools | Presenter Info | Resources
OUTLINE
NOTES
Search
Clinical Process
Physical Examination
Signs of emphysema:
barrel chest (form of thorax)
silent chest (decreased breath sounds)
cyanosis
coarse crepitations
expiratory rhonchi...
1
Patient history
Physical Examination
2
3
Tests
12. ---
13. ---
14. ---
15. ---
16. ---
17. ---
18. ---
19. ---
20. ---
PREV
NEXT

Marker Tools | Presenter Info | Resources

OUTLINE NOTES

Search

12. --- 13. --- 14. --- 15. --- 16. --- 17. --- 18. --- 19. --- 20. ---

Clinical Process

Tests

Usually with history and examination that show signs of COPD:

- **Spirometry**

Other possible test (for differential diagnoses) include:

- sputum culture
- chest x-ray
- ultrasound
- blood tests

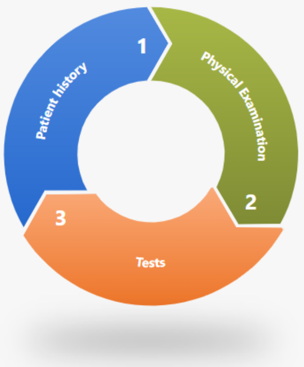

PREV NEXT

Marker Tools | Presenter Info | Resources

OUTLINE NOTES

Search

13. --- 14. --- 15. --- 16. --- 17. --- 18. --- 19. --- 20. --- 21. ---

Spirometry

What is spirometry?

What is spirometry?

- An objective lung functioning test to measure airflow limitation
- COPD and Asthma both show airflow obstruction, which means air is trapped inside the lung (vs. airflow restriction which means lungs can't expand much)

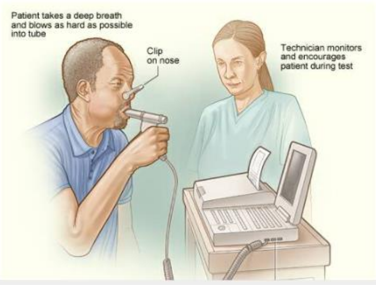

How to perform spirometry

Spirometric values in COPD

Alternative diagnostic tools for COPD

PREV NEXT

Marker Tools | Presenter Info | Resources

OUTLINE NOTES

Search

13. ---
14. ---
15. ---
16. ---
17. ---
18. ---
19. ---
20. ---
21. ---

Spirometry

What is spirometry? | How to perform spirometry | Spirometric values in COPD | Alternative diagnostic tools for COPD

How to perform spirometry

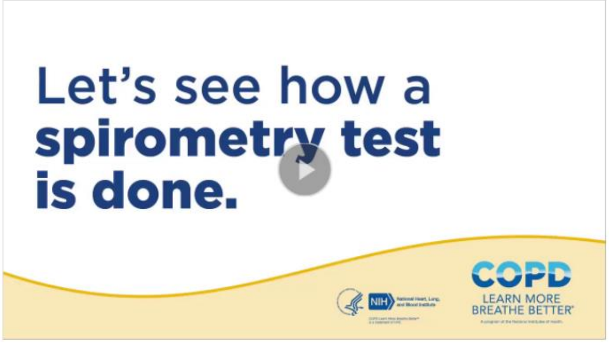

https://www.youtube.com/watch?v=Zs8FsSHajHs

PREV NEXT

Marker Tools | Presenter Info | Resources

OUTLINE NOTES

Search

13. ---
14. ---
15. ---
16. ---
17. ---
18. ---
19. ---
20. ---
21. ---

Spirometry

What is spirometry? | How to perform spirometry | Spirometric values in COPD | Alternative diagnostic tools for COPD

Spirometric values in COPD

- **low FEV1** (Forced Expiratory Volume in 1 second)
- **low FEV1/FVC ratio** (Forced Expiratory Volume in 1 second/Forced Vital Capacity ratio)
- To distinguish Asthma and COPD a fast-acting **bronchodilator** (i.e. Salbutamol) is needed (Asthma has a reversible airflow obstruction)
- If **post-bronchodilator FEV1/FVC ratio** is still **< 0.7 (70%)**, then a COPD is likely

Perfect example of COPD spirometry curve (blue=COPD):

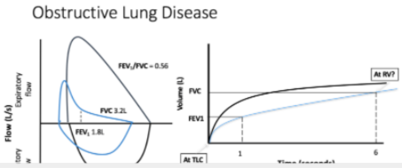

PREV NEXT

OUTLINE NOTES

Search

Spirometry

What is spirometry? | How to perform spirometry | **Spirometric values in COPD** | Alternative diagnostic tools for COPD

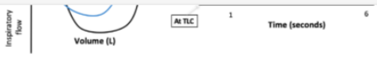

https://thecurbsiders.com/podcast/86-copd-diagnosis-treatment-pfts-nihilism

Real life example of COPD spirometry curve (blue=COPD): A Flow-Volume curve, like the first graph above, is pictured

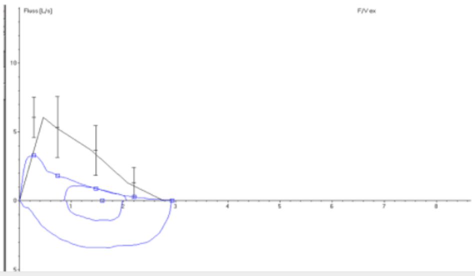

13. ---
14. ---
15. ---
16. ---
17. ---
18. ---
19. ---
20. ---
21. ---

PREV NEXT

Marker Tools | Presenter Info | Resources

OUTLINE NOTES

Search

Spirometry

What is spirometry? | How to perform spirometry | Spirometric values in COPD | **Alternative diagnostic tools for COPD**

Alternative diagnostic tools for COPD

- Spirometry is often not available in Zambia, alternative diagnostic tools have been developed
- Available tools are: Microspirometers (small, handheld spirometers), questionnaires or PEF devices (Peak Expiratory Flow)

Examples:

- COPD-6 Microspirometer
- CDQ (COPD Diagnostic Questionnaire)
- CAPTURE Questionnaire in combination with PEF

13. ---
14. ---
15. ---
16. ---
17. ---
18. ---
19. ---
20. ---
21. ---

PREV NEXT

Resources | Question 1 of 1 | Your Score: 0 of 10

OUTLINE NOTES

Search

15. ---

16. ---

17. ---

18. ---

19. ---

20. ---

21. ---

22. ---

22.1. Match the spirometric curves with their descri...

Match the spirometric curves with their descriptions:

COPD	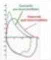
Restriction	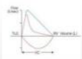
Asthma	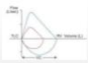
Normal	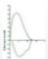

SUBMIT

Resources | Question 1 of 1 | Your Score: 0 of 10

OUTLINE NOTES

Search

15. ---

16. ---

17. ---

18. ---

19. ---

20. ---

21. ---

22. ---

22.1. Match the spirometric curves with their descri...

Match the spirometric curves with their descriptions:

COPD

Restriction

Asthma

Normal

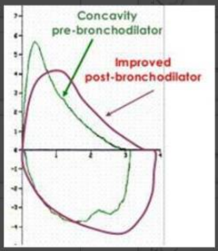

SUBMIT

Resources | Question 1 of 1 | Your Score: 0 of 10

OUTLINE NOTES

Search

15. ---

16. ---

17. ---

18. ---

19. ---

20. ---

21. ---

22. ---

22.1. Match the spirometric curves with their descri...

Match the spirometric curves with their descriptions:

COPD 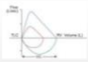

Restriction 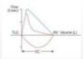

Asthma 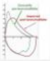

Normal 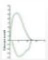

SUBMIT

Resources | Question 1 of 1 | Your Score: 10 of 10

OUTLINE NOTES

Search

15. ---

16. ---

17. ---

18. ---

19. ---

20. ---

21. ---

22. ---

22.1. Match the spirometric curves with their descri...

Match the spirometric curves with their descriptions:

COPD 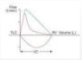

Restriction 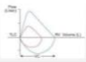

Asthma 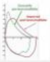

Normal 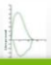

Correct

That's right! You chose the correct response.

VIEW RESULTS

Resources

Congratulations, you passed!

Your Score: **100% (10 points)**

Passing Score: **80% (8 points)**

REVIEW QUIZ

OUTLINE NOTES

Search

15. ---
16. ---
17. ---
18. ---
19. ---
20. ---
21. ---
22. ---
23. ---

PREV NEXT

Marker Tools | Presenter Info | Resources

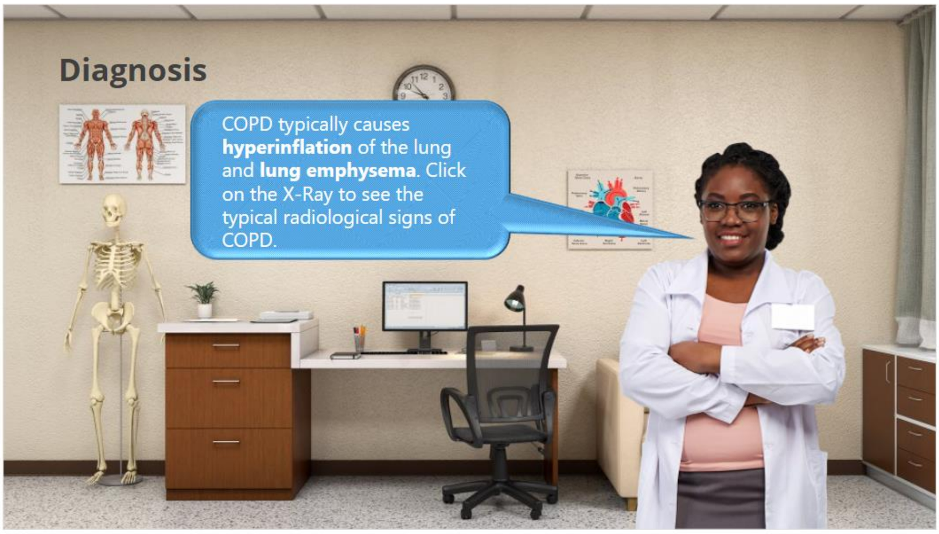

23 / 39 00:01 / 00:11

PREV NEXT

OUTLINE NOTES

Search

15. ---
16. ---
17. ---
18. ---
19. ---
20. ---
21. ---
22. ---
23. ---

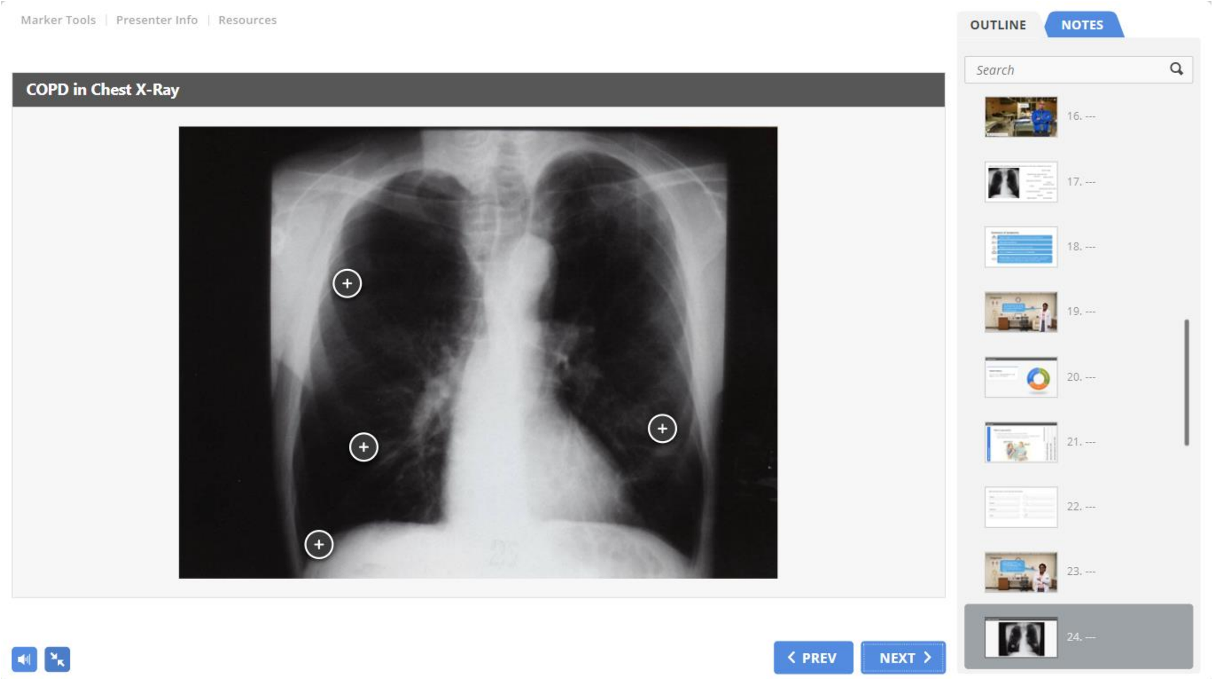
Marker Tools | Presenter Info | Resources
COPD in Chest X-Ray
OUTLINE
NOTES
Search
16. ---
17. ---
18. ---
19. ---
20. ---
21. ---
22. ---
23. ---
24. ---
PREV
NEXT

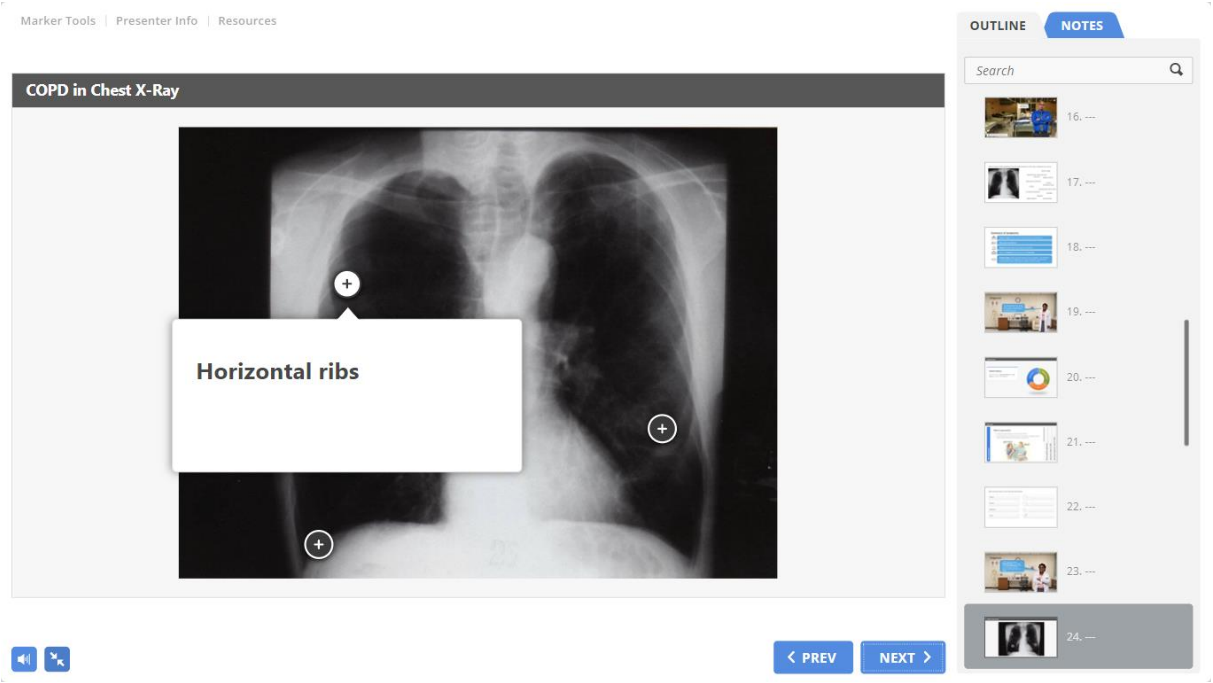
Marker Tools | Presenter Info | Resources
COPD in Chest X-Ray
Horizontal ribs
OUTLINE
NOTES
Search
16. ---
17. ---
18. ---
19. ---
20. ---
21. ---
22. ---
23. ---
24. ---
PREV
NEXT

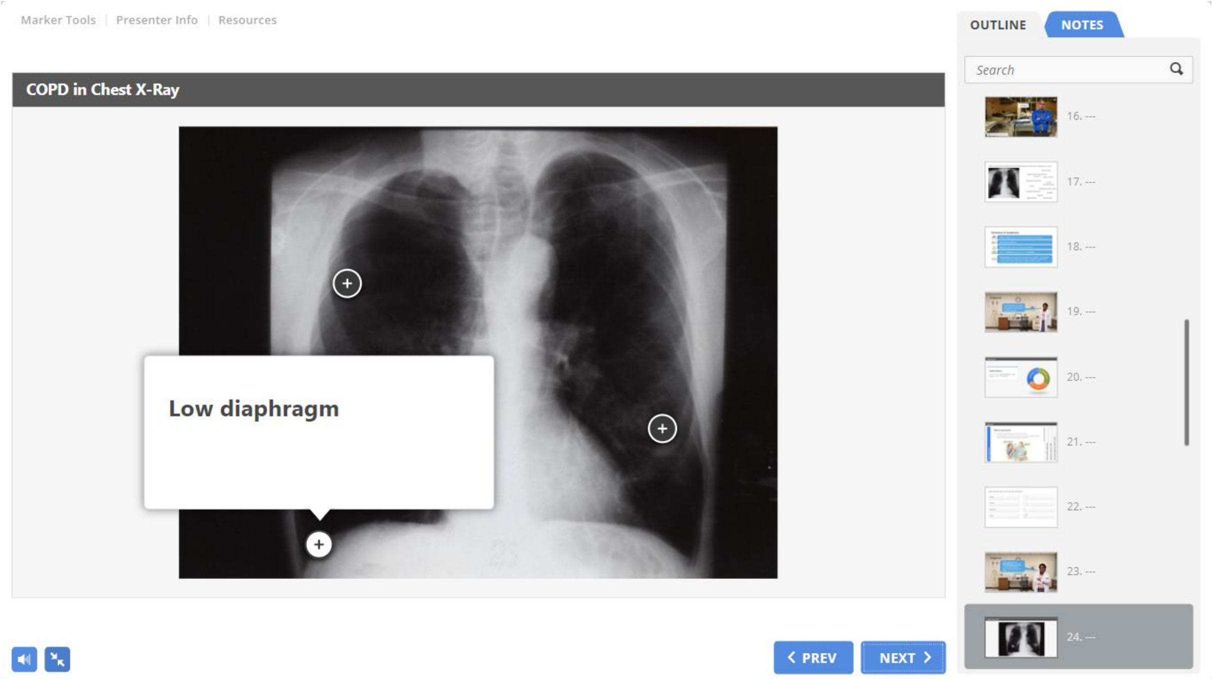
Marker Tools
Presenter Info
Resources
COPD in Chest X-Ray
Low diaphragm
PREV
NEXT
OUTLINE
NOTES
Search
16. ---
17. ---
18. ---
19. ---
20. ---
21. ---
22. ---
23. ---
24. ---

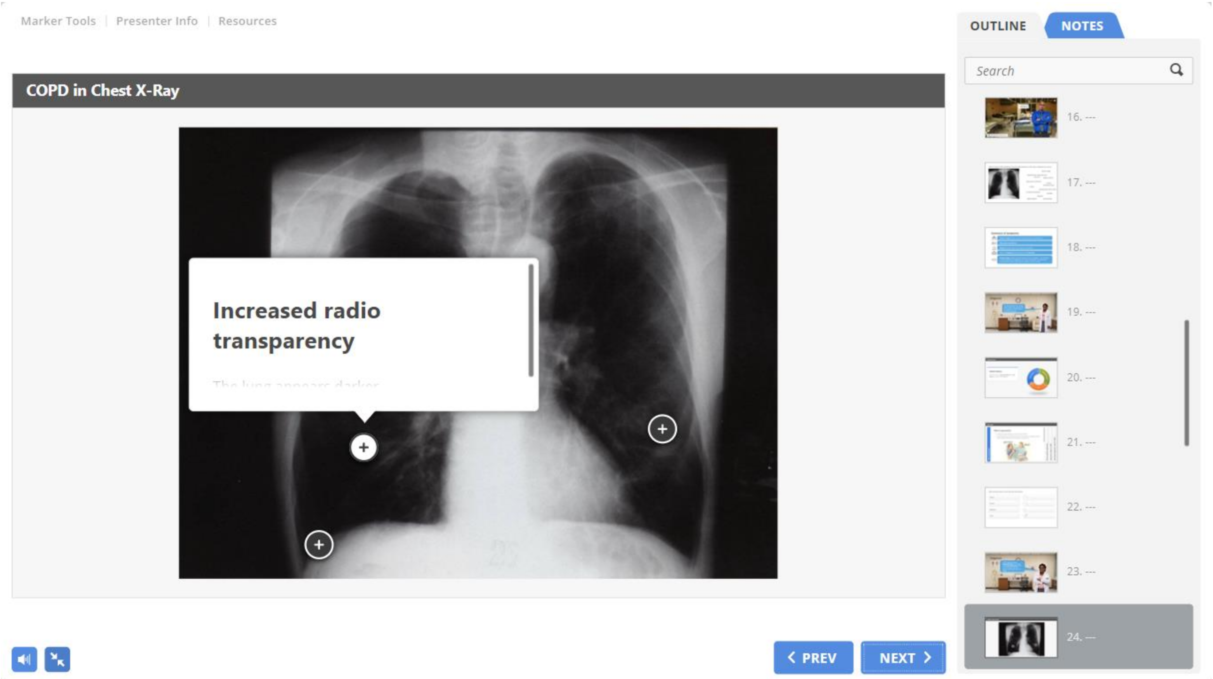
Marker Tools
Presenter Info
Resources
COPD in Chest X-Ray
Increased radio transparency
PREV
NEXT
OUTLINE
NOTES
Search
16. ---
17. ---
18. ---
19. ---
20. ---
21. ---
22. ---
23. ---
24. ---

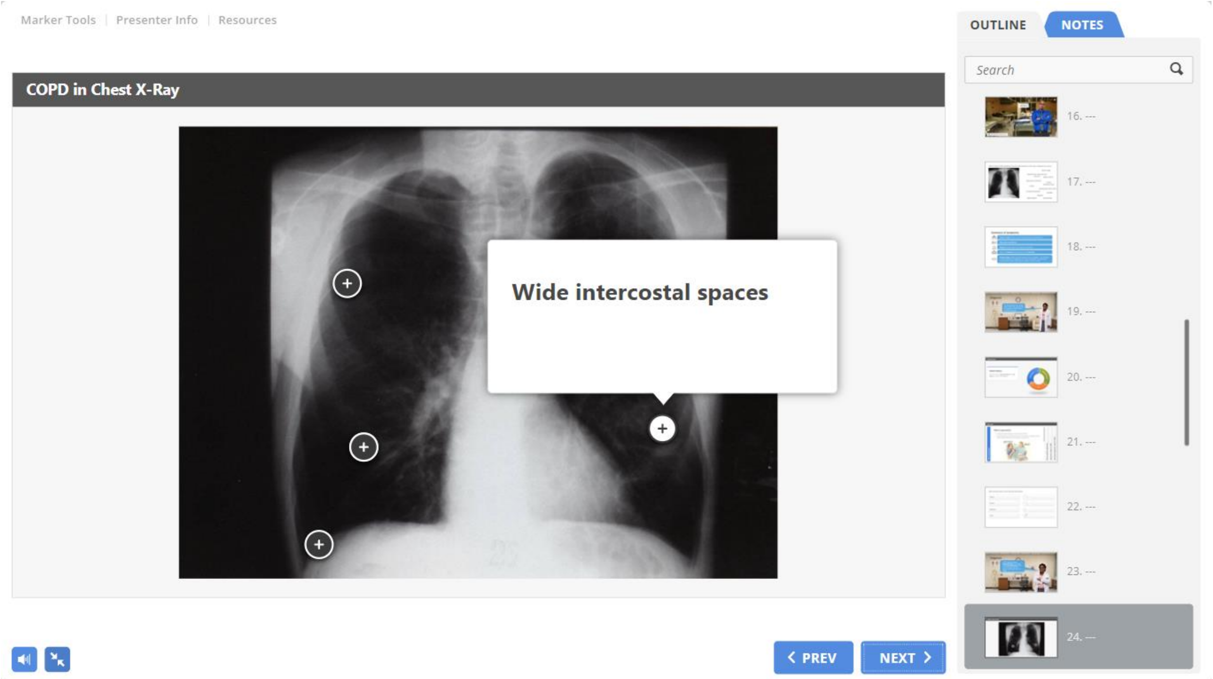
Marker Tools | Presenter Info | Resources
COPD in Chest X-Ray
Wide intercostal spaces
PREV
NEXT
OUTLINE
NOTES
Search
16. ---
17. ---
18. ---
19. ---
20. ---
21. ---
22. ---
23. ---
24. ---

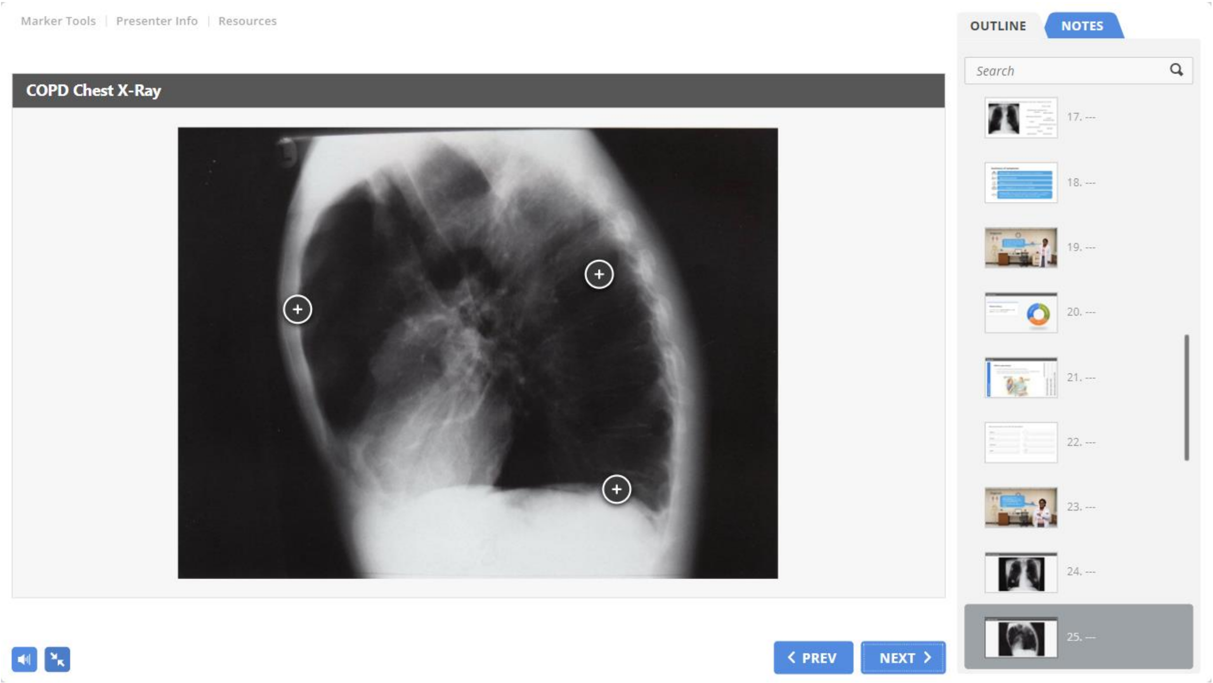
Marker Tools | Presenter Info | Resources
COPD Chest X-Ray
PREV
NEXT
OUTLINE
NOTES
Search
17. ---
18. ---
19. ---
20. ---
21. ---
22. ---
23. ---
24. ---
25. ---

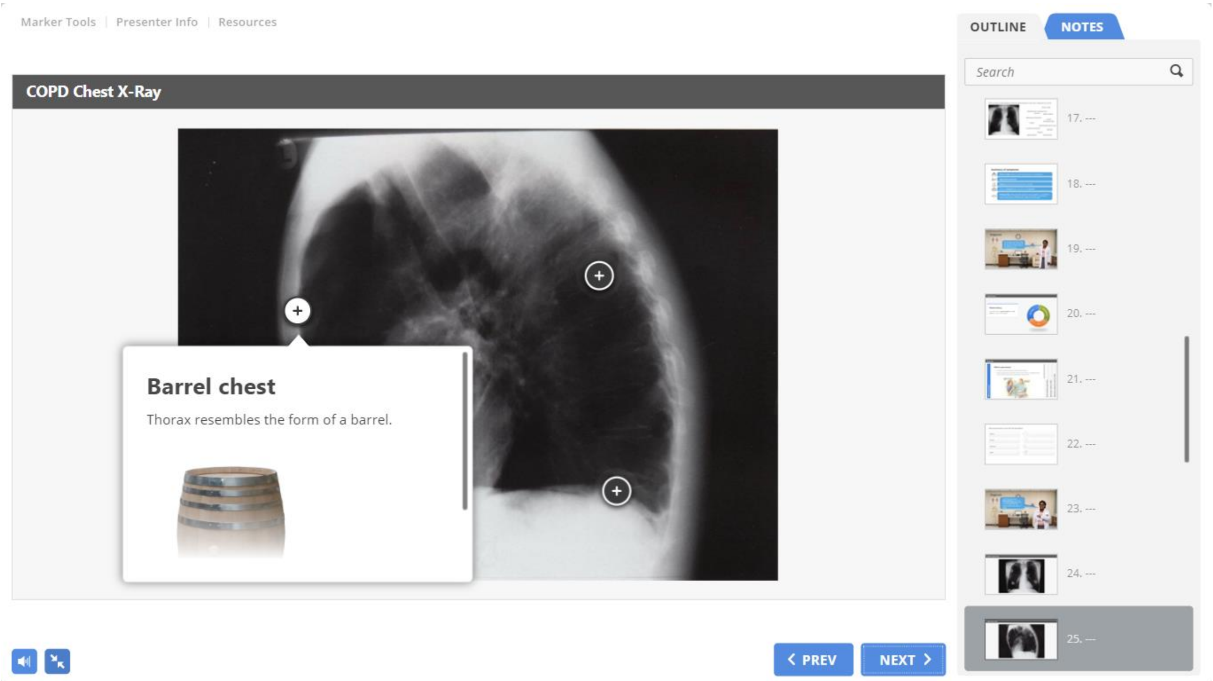
Marker Tools | Presenter Info | Resources
COPD Chest X-Ray
Barrel chest
Thorax resembles the form of a barrel.
OUTLINE
NOTES
Search
17. ---
18. ---
19. ---
20. ---
21. ---
22. ---
23. ---
24. ---
25. ---
PREV
NEXT

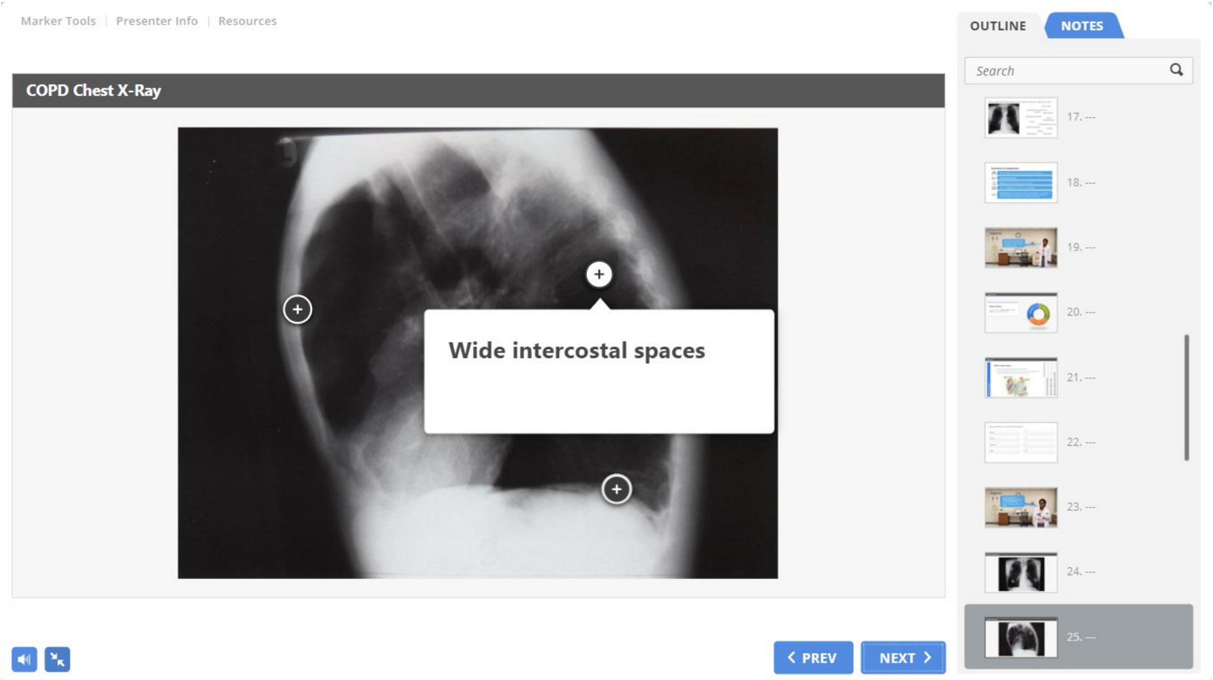
Marker Tools | Presenter Info | Resources
COPD Chest X-Ray
Wide intercostal spaces
OUTLINE
NOTES
Search
17. ---
18. ---
19. ---
20. ---
21. ---
22. ---
23. ---
24. ---
25. ---
PREV
NEXT

Marker Tools | Presenter Info | Resources

OUTLINE NOTES

Search

17. ---
18. ---
19. ---
20. ---
21. ---
22. ---
23. ---
24. ---
25. ---

COPD Chest X-Ray

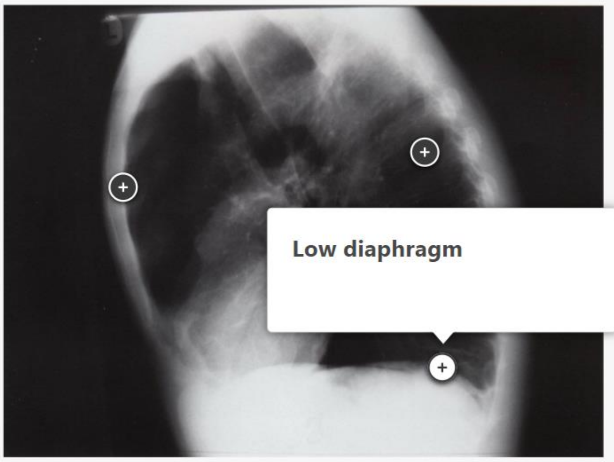

PREV NEXT

Marker Tools | Presenter Info | Resources

OUTLINE NOTES

Search

18. ---
19. ---
20. ---
21. ---
22. ---
23. ---
24. ---
25. ---
26. ---

Classification of severity

2 classifications made by GOLD (Global Initiative of Obstructive Lung Disease)

Severity of airflow limitation
Determined by spirometry

GOLD group	FEV1 (% of predicted)
1	≥80
2	50-79
3	30-49
4	<30

ABCD-Scheme
Determined by symptoms (CAT+mMRC) + number of exacerbations

Patient group	Exacerbations/ year	mMRC	CAT
A	≤1	0-1	<10
B	≤1	≥2	≥10
C	≥2	0-1	<10
D	≥2	≥2	≥10

26 / 39 01:06 / 01:06

PREV NEXT

Marker Tools | Presenter Info | Resources

OUTLINE NOTES

Search

19. ---
20. ---
21. ---
22. ---
23. ---
24. ---
25. ---
26. ---
27. ---

Symptom evaluation

CAT Questionnaire (COPD Assessment Test) → measures symptoms

- Comprises 8 questions about cough, sputum production, chest tightness, dyspnea, limitation of physical activity, fear of leaving the house, sleep, and energy; each to be recorded on a scale of 1 – 6

mMRC Questionnaire (Modified British Medical Research Council) → measures dyspnea

- 0 = dyspnea on strong exertion
- 1 = dyspnea when climbing stairs
- 2 = dyspnea when walking on even ground
- 3 = dyspnea when walking on even ground, < 100 m
- 4 = dyspnea when getting dressed / undressed

27 / 39 00:22 / 00:22

PREV NEXT

Resources | Question 1 of 2 | Your Score: 10 of 20

OUTLINE NOTES

Search

21. ---
22. ---
23. ---
24. ---
25. ---
26. ---
27. ---
28. ---

28.1. Our patient Mr. Abebe said he has a shortness ...

Our patient Mr. Abebe said he has a shortness of breath already when he isn't doing much. After further inquiry he states that he already has trouble breathing when he gets dressed. He scored 20 in the CAT Questionnaire and he had two exacerbations in the last year. His FEV1 value is 30%. Which COPD group would he be in (1-4)? Select the correct answer option:

- ○ Group 2
- ○ Group 4
- ◉ Group 3
- ○ Group 1

Correct

That's right! You chose the correct response.

CONTINUE

Which group would he be in according to the ABCD-Scheme? Select the correct answer option:

- B
- C
- D (selected)
- A

Correct

That's right! You chose the correct response.

VIEW RESULTS

OUTLINE NOTES
Search
22. ---
23. ---
24. ---
25. ---
26. ---
27. ---
28. ---
28.1. Our patient Mr. Abebe said he has a shortness ...
28.2. Which group would he be in according to the...

Congratulations, you passed!

Your Score: **100% (20 points)**

Passing Score: **80% (16 points)**

REVIEW QUIZ

PREV NEXT

OUTLINE NOTES
Search
22. ---
23. ---
24. ---
25. ---
26. ---
27. ---
28. ---
29. ---
30. ---

Marker Tools | Presenter Info | Resources

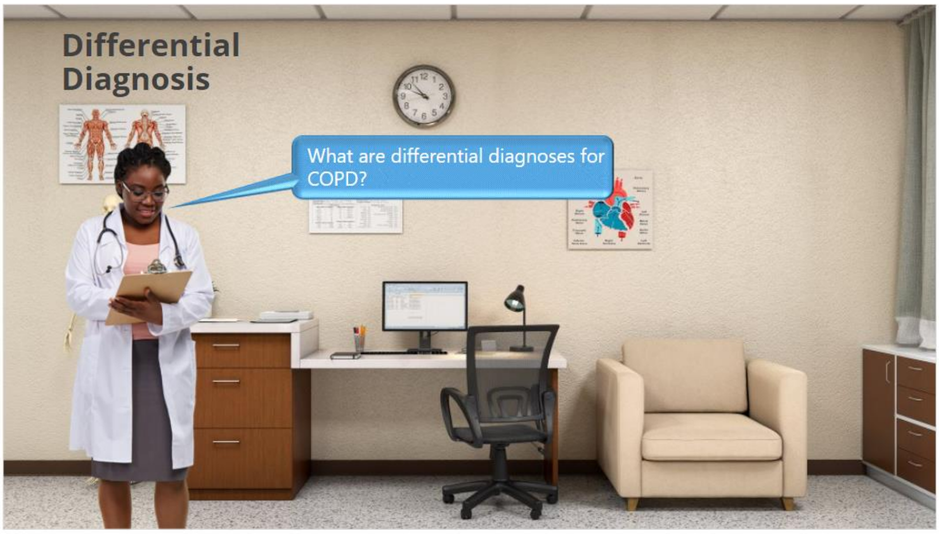

29 / 39 00:03 / 00:04

PREV NEXT

OUTLINE NOTES

Search

22. ---
23. ---
24. ---
25. ---
26. ---
27. ---
28. ---
29. ---
30. ---

Resources | Question 1 of 2 | Your Score: 0 of 20

Select possible differential diagnoses for COPD. (4 answers are correct)

- [ ] Gastroesophageal reflux
- [ ] Bowel carcinoma
- [ ] Tuberculosis
- [ ] Trauma
- [ ] Heart attack
- [ ] Congestive Heart Failure
- [ ] Asthma
- [ ] Tonsillitis

SUBMIT

OUTLINE NOTES

Search

23. ---
24. ---
25. ---
26. ---
27. ---
28. ---
29. ---
30. ---
30.1. Select possible differential diag...

Resources | Question 1 of 2 | Your Score: 10 of 20

OUTLINE NOTES

Search

Select possible differential diagnoses for COPD. (4 answers are correct)

- [ ] Heart attack
- [x] Tuberculosis
- [x] Asthma
- [ ] Bowel carcinoma
- [ ] Trauma
- [x] Congestive Heart Failure
- [ ] Tonsillitis
- [x] Gastroesop[illegible]

Correct

That's right! You chose the correct response.

27. ---

28. ---

29. ---

30. ---

30.1. Select possible differential diag...

30.2. Select possible differential diag...

31. ---

32. ---

33. ---

CONTINUE

Resources | Question 2 of 2 | Your Score: 20 of 20

OUTLINE NOTES

Search

Select possible differential diagnoses for COPD. (3 answers are correct)

- [x] Bronchial carcinoma
- [x] Bronchiectasis
- [ ] Stomach ulcera
- [ ] Tooth abcess
- [ ] Hypochondria
- [x] Pulmonary embolism

Correct

That's right! You chose the correct response.

27. ---

28. ---

29. ---

30. ---

30.1. Select possible differential diag...

30.2. Select possible differential diag...

31. ---

32. ---

33. ---

VIEW RESULTS

Search

27. ---
28. ---
29. ---
30. ---
31. ---
32. ---
33. ---
34. ---
35. ---

Congratulations, you passed!

Your Score: **100% (20 points)**

Passing Score: **80% (16 points)**

REVIEW QUIZ

OUTLINE NOTES

Search

27. ---
28. ---
29. ---
30. ---
31. ---
32. ---
33. ---
34. ---
35. ---

Differential Diagnosis

- Asthma: early onset in life, reversible airflow obstruction, symptoms vary daily, symptom-free intervals, allergic component → **Bronchodilator test**
- Tuberculosis: **Chest X-Ray** and **microbiological confirmation**
- Congestive Heart Failure: Dilated heart in **chest X-Ray** and pulmonary edema → **restriction**, not obstruction
- Bronchiectasis: High sputum production, **chest X-Ray** shows dilated bronchi and thick bronchial walls
- Bronchial carcinoma: **Chest X-Ray**
- Gastroesophageal reflux: can cause chronic cough
- Pulmonary embolism
- Vocal cord dysfunction

→ Diagnosis by exclusion, bronchial carcinoma and Tuberculosis need to be ruled out!

Therapy

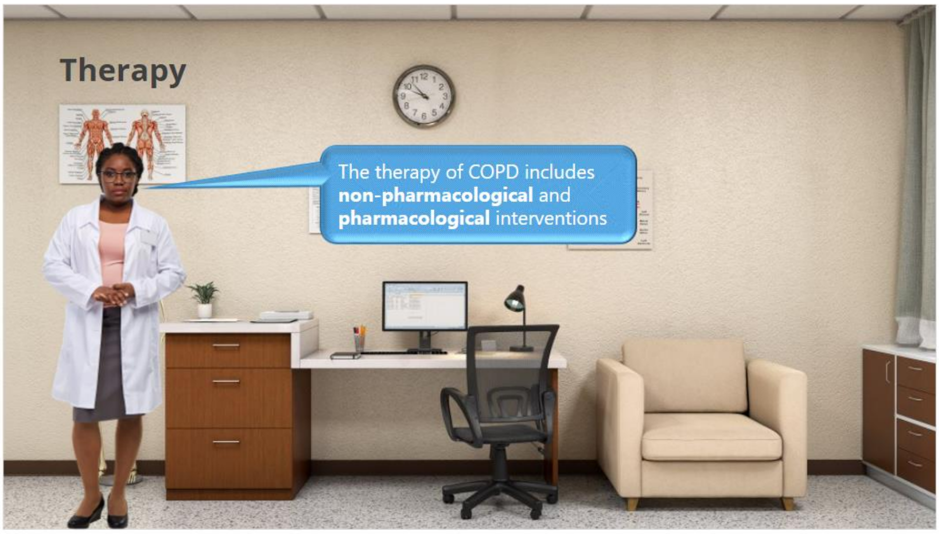

Marker Tools | Presenter Info | Resources

OUTLINE NOTES

Search

27. ---
28. ---
29. ---
30. ---
31. ---
32. ---
33. ---
34. ---
35. ---

Non-pharmacological

- Smoking Cessation
- Vaccinations
- Pulmonary rehabilitation
- Osteoporosis prophylaxis
- Oxygen therapy

Smoking Cessation

Quitting smoking is one of the most important interventions for COPD management.

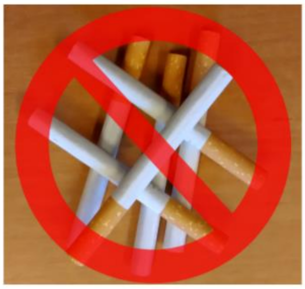

www.pixabay.com

PREV NEXT

Non-pharmacological

- Smoking Cessation
- Vaccinations
- Pulmonary rehabilitation
- Osteoporosis prophylaxis
- Oxygen therapy

Vaccinations

Pneumococcal and **Influenza vaccine**.

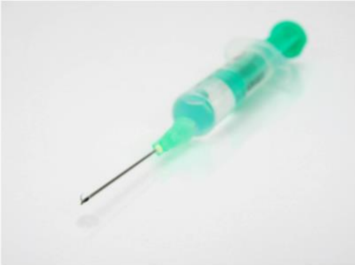

www.pixabay.com

27. ---
28. ---
29. ---
30. ---
31. ---
32. ---
33. ---
34. ---
35. ---

Non-pharmacological

- Smoking Cessation
- Vaccinations
- Pulmonary rehabilitation
- Osteoporosis prophylaxis
- Oxygen therapy

Pulmonary rehabilitation

- education
- respiratory exercises
- physical activity
- self-management interventions

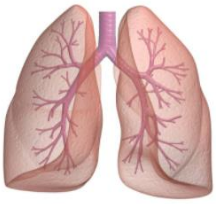

27. ---
28. ---
29. ---
30. ---
31. ---
32. ---
33. ---
34. ---
35. ---

Marker Tools | Presenter Info | Resources

Non-pharmacological

- Smoking Cessation
- Vaccinations
- Pulmonary rehabilitation
- **Osteoporosis prophylaxis**
- Oxygen therapy

Osteoporosis prophylaxis

With **Vitamin D3** and **Calcium**. The lifestyle of COPD patients but also the treatment with corticosteroids can increase risk of osteoporosis.

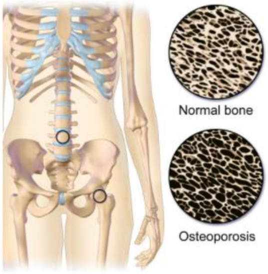

OUTLINE | NOTES

Search

27. ---
28. ---
29. ---
30. ---
31. ---
32. ---
33. ---
34. ---
35. ---

PREV | NEXT

Marker Tools | Presenter Info | Resources

Non-pharmacological

- Smoking Cessation
- Vaccinations
- Pulmonary rehabilitation
- Osteoporosis prophylaxis
- **Oxygen therapy**

Oxygen therapy

Oxygen therapy is indicated if paO2 is <55 mmHg or <60 mmHg with right heart failure.

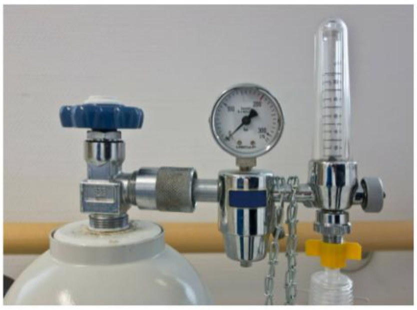

OUTLINE | NOTES

Search

27. ---
28. ---
29. ---
30. ---
31. ---
32. ---
33. ---
34. ---
35. ---

PREV | NEXT

Pharmacological

- Bronchodilators
- Roflumilast
- Theophyllin

Bronchodilators

- **Beta-2 Agonists**: Short acting (SABA) or long acting (LABA)
- **Anticholinergics**: Short acting (SAMA) or long acting (LAMA)

--> Short acting bronchodilators for acute relief when needed, long acting bronchodilators for long-term therapy

Corticosteroids:

- **ICS** (inhaled corticosteroids): only in combination with LABA

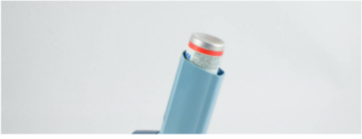

Marker Tools | Presenter Info | Resources

Pharmacological

- Bronchodilators
- Roflumilast
- Theophyllin

Roflumilast

Selective PDE4-inhibitor with strong anti-inflammatory effect, indicated in severe COPD III/IV.

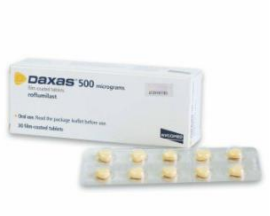

https://rtmagazine.com/products-treatment/pharmaceuticals/us-pharmaceuticals/roflumilast-partly-reverses-smoking-related-mucociliary-dysfunction/

OUTLINE NOTES
Search
27. ---
28. ---
29. ---
30. ---
31. ---
32. ---
33. ---
34. ---
35. ---
PREV NEXT

Marker Tools | Presenter Info | Resources

OUTLINE NOTES

Search

27. ---
28. ---
29. ---
30. ---
31. ---
32. ---
33. ---
34. ---
35. ---

Pharmacological

Bronchodilators

Roflumilast

Theophyllin

Theophyllin

Because of low efficiency and relevant adverse effects / interactions only used as reserve.

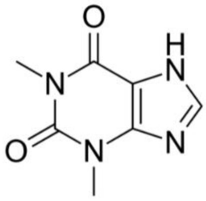

https://en.wikipedia.org/wiki/Theophylline

PREV NEXT

Marker Tools | Presenter Info | Resources

OUTLINE NOTES

Search

27. ---
28. ---
29. ---
30. ---
31. ---
32. ---
33. ---
34. ---
35. ---

Therapy

Group	Therapy
A	Bronchodilators (short or long acting)
B	LAMA or LABA, if not enough: LAMA+LABA
C	LAMA, with continuous exacerbations: LAMA+LABA or LABA+ICS
D	LAMA+LABA<br>With continuous exacerbations: LAMA+LABA+ICS<br>Possibly Roflumilast

35 / 39 00:22 / 00:22

PREV NEXT

Resources | Question 1 of 1 | Your Score: 10 of 10

OUTLINE NOTES

Search

29. ---
30. ---
31. ---
32. ---
33. ---
34. ---
35. ---
36. ---
36.1. Which therapy would be adequate for Mr....

Which therapy would be adequate for Mr. Abebe?

- ( ) Beta-blocker
- ( ) LAMA
- (•) LAMA+LABA+ICS
- ( ) LAMA+ICS
- ( ) LABA

Correct

That's right! You chose the correct response.

VIEW RESULTS

Resources

OUTLINE NOTES

Search

29. ---
30. ---
31. ---
32. ---
33. ---
34. ---
35. ---
36. ---
37. ---

Congratulations, you passed!

Your Score: **100% (10 points)**

Passing Score: **80% (8 points)**

REVIEW QUIZ

PREV NEXT

Prognosis

- **Important: early diagnosis!**
- COPD can be treated and its progression can be slowed when diagnosed early
- 30% of COPD patients are diagnosed when already in GOLD stages 3 or 4
- → for patients in GOLD stages 3 and 4 life expectancy is significantly reduced

Risk factors for an unfavourable prognosis:
➢Many exacerbations
➢Old age
➢Hypercapnia
➢Long-term therapy with corticosteroids
➢Other severe comorbidity (heart failure…)

Marker Tools | Presenter Info | Resources

Sources

- GOLD Report 2020
- Amboss, Chapter „COPD“
- Herold, Gerd: Innere Medizin, Köln. 2019., pages 348-354

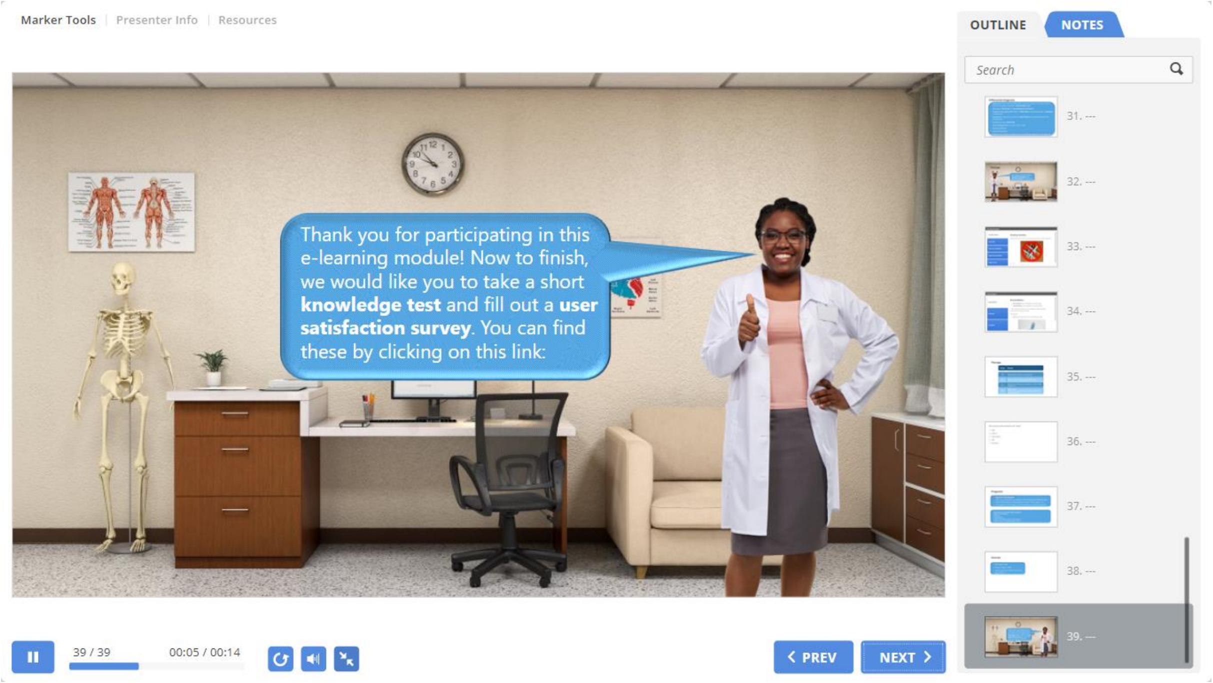
Marker Tools
Presenter Info
Resources
Thank you for participating in this e-learning module! Now to finish, we would like you to take a short **knowledge test** and fill out a **user satisfaction survey**. You can find these by clicking on this link:
39 / 39
00:05 / 00:14
PREV
NEXT
OUTLINE
NOTES
Search
31. ---
32. ---
33. ---
34. ---
35. ---
36. ---
37. ---
38. ---
39. ---
