# Supplementary material for: Real-life Evaluation of an Interactive Versus Noninteractive e-Learning Module on Chronic Obstructive Pulmonary Disease for Medical Licentiate Students in Zambia: Web-Based, Mixed Methods Randomized Controlled Trial
Source: JMIR Med Educ. 2022 Feb 24;8(1):e34751. doi: 10.2196/34751 (PMC8914755; doi:10.2196/34751)
Supplement: Multimedia Appendix 4 [file mededu_v8i1e34751_app4.pdf]

User satisfaction questionnaire

For each statement below circle the response that best characterizes how you feel about the statement.

1= Strongly Disagree, 2= Disagree, 3= Neither Agree Nor Disagree, 4= Agree, 5= Strongly Agree

	Strongly Disagree	Disagree	Neither Agree Nor Disagree	Agree	Strongly Agree
**1.** I enjoyed completing the learning unit.	1	2	3	4	5
**2.** I am satisfied with the learning unit.	1	2	3	4	5
**3.** My knowledge of COPD increased significantly.	1	2	3	4	5
**4.** My interest in COPD increased.	1	2	3	4	5
**5.** The key messages of the learning unit were clear.	1	2	3	4	5
**6.** The unit provides information relevant for medical practice.	1	2	3	4	5
**7.** It was easy to learn with the learning unit.	1	2	3	4	5
**8.** I would recommend the learning unit to a friend.	1	2	3	4	5

System Usability Scale

		Strongly disagree 1	2	3	4	Strongly agree 5
1.	I think that I would like to use this e-learning method frequently.	○	○	○	○	○
2.	I found the e-learning method unnecessarily complex.	○	○	○	○	○
3.	I thought the e-learning method was easy to use.	○	○	○	○	○
4.	I think that I would need the support of a technical person to be able to use this e-learning method.	○	○	○	○	○
5.	I found the various functions in this e-learning method were well integrated.	○	○	○	○	○
6.	I thought there was too much inconsistency in this e-learning method.	○	○	○	○	○
7.	I would imagine that most people would learn to use this e-learning method very quickly.	○	○	○	○	○
8.	I found the e-learning method very hard to use.	○	○	○	○	○
9.	I felt very confident using the e-learning method.	○	○	○	○	○
10.	I needed to learn a lot of things before I could get going with this e-learning method.	○	○	○	○	○

System Usability Scale, © Digital Equipment Corporation, 1986

Knowledge Tests 1+2

First knowledge test:

1. What is part of the definition of COPD? (COPD definition)
A) not preventable disease
B) variable respiratory symptoms
C) reversible airflow obstruction
D) not curable
E) exposure to particles, gases, and pollen

2. Which of the following is a symptom of COPD? (COPD symptoms)
A) dry cough
B) sinusitis
C) cyanosis
D) oral thrush
E) pectus excavatum

3. What is a typical medical finding in a physical examination of a COPD patient? (COPD PE)
A) prolonged inspiration
B) bronchial breathing sound in basal parts of the lungs
C) high dorsobasal lung borders
D) increased bronchophony
E) hyper resonant percussion sound

4. A long-time, 73-year old COPD patient is admitted to hospital with worsening dyspnea, chronic cough, and fatigue with phases of somnolence. Her usual treatment encompasses long-term beta-2-agonists, anticholinergics, Theophylline, thiazides, and a long-term oxygen therapy. Due to the exacerbation the therapy is being modified. Which additional therapy is indicated? (COPD therapy)
A) beta-blocker because of tachycardia
B) temporary application of systemic glucocorticoids as anti-inflammatory therapy
C) restrictive intake of fluids because of pulmonary hypertension
D) inhibition of leukotrienes as mediators through leukotriene-receptor inhibitor
E) anti-histamines for H1-Receptor inhibition

5. Which pathogens are typical for exacerbations? (COPD pathogens/vaccinations)
A) Hemophilus ducreyi and Streptococcus pyogenes
B) Hemophilus influenzae and Streptococcus pneumoniae
C) Mycobacterium tuberculosis and Respiratory-Syncytial-Virus
D) Legionella pneumophila and Bordetella pertussis
E) Streptococcus pneumoniae and Adenovirus

6. A 19-year old patient is presenting herself with a cough. She has been coughing for about a month now and taking ACC and Paracetamol has not helped. She has been smoking 10-20 cigarettes/day since she was 14 years old. These are her spirometry results: VC (Vital capacity) 95% of reference value, FEV1 (Forced Expiratory Volume in 1 Second) 60% of reference value. After the application of beta-2-agonist the FEV1 value normalizes. Which diagnosis is correct? (COPD Dif.Diagnosis)
A) lung emboli
B) bronchial asthma
C) COPD

D) house-dust allergy
E) recurrent laryngeal nerve paralysis

7. Which genetic disposition is a risk factor for COPD? (COPD risk factors)
A) alpha-2-antitrypsin deficiency
B) beta-1-antitrypsin deficiency
C) alpha-1-antitrypsin deficiency
D) 5-alpha reductase deficiency
E) C1-esterase inhibitor deficiency

8. Your patient has a FEV1 of 48%. Which GOLD group (1-4) would he be in? (GOLD 1-4)
A) 1
B) 2
C) 3
D) 4

9. Which radiological sign is **the least** typical for lung emphysema? (COPD X-Ray)
A) barrel chest
B) increased radio transparency of lung
C) low diaphragm level
D) wide intercostal spaces
E) perihilar edema

10. Which of the following is **not** essential in the management of COPD? (COPD management)
A) diagnostics of osteoporosis
B) physical training to increase muscle growth
C) yearly active immunization against influenza
D) inhaling of beta-blockers
E) special breathing physiotherapy

11. You are treating a patient in COPD group C with continuous exacerbations. Which therapy is indicated according to the ABCD-Scheme? (COPD therapy 2)
A) LAMA+LABA or LABA+ICS
B) LAMA or LABA
C) LAMA+LABA+ICS
D) LAMA
E) Beta-blocker

12. Which of the following spirometric findings can be found in COPD patients? (% of reference value) (COPD Spiro 1)
A) PEF 95% (Peak Expiratory Flow)
B) FEV1 89% (Forced Expiratoy Volume in 1 Sec.)
C) FEV1/FVC 53% (Forced Expiratory Volume in 1 Sec./Forced Vital Capacity)
D) RV 55% (Residual Volume)
E) TLC 40% (Total Lung Capacity)

13. Which Flow-Volume curve is typical for severe COPD?

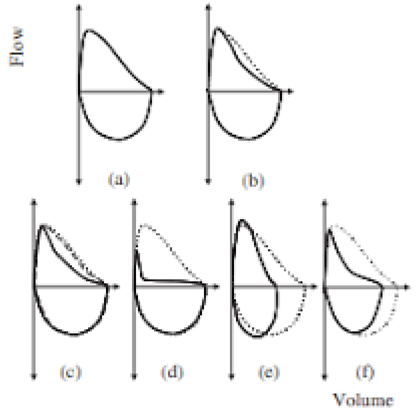

https://copdx.org.au/copd-x-plan/confirm-diagnosis/c2-diagnosis/c23-spirometry/

A) (a)
B) (b)
C) (c)
D) (d)
E) (e)
F) (f)

14. Mr. Balewa is 75 years old and a long-term COPD patient. He has smoked 20 cigarettes a day for 50 years of his life and now has a chronic cough and dyspnea when climbing stairs. He scored 9 in the COPD Assessment Test (CAT). Last year, he had two exacerbations. In which group of the ABCD-Scheme is Mr. Balewa?
A) Group A
B) Group B
C) Group C
D) Group D

15. Which statements about the worldwide epidemiology of COPD is correct? (COPD epidemiology)
A) Global prevalence of COPD is 6%.
B) COPD is the 3rd leading cause of death in adults.
C) 70% of COPD deaths occur in Low-and Middle-Income countries.
D) Women are more affected than men.
E) Prevalence is predicted to decrease due to efficient prevention programs.

Second knowledge test (after a few weeks):

1. What is part of the definition of COPD? (COPD definition)
A) preventable disease
B) communicable disease
C) reversible airflow obstruction
D) curable
E) large allergic component

2. Which of the following is a symptom of COPD? (COPD symptoms)
A) sinusitis
B) epiglottitis

C) dry cough
D) pursing of lips
E) low sputum production

3. What is a typical sign in the physical examination of a COPD patient? (COPD PE)
A) distinct inspiratory stridor
B) bronchial breathing over periphery lung parts
C) high-pitched crepitations on both sides dorsal
D) positive bronchophony
E) attenuated breath sounds on both sides

4. A long-time, 73-year old COPD patient is admitted to hospital with worsening dyspnea, chronic cough, and fatigue with phases of somnolence. Which medicament is **the least** indicated? (COPD therapy)
A) long-term beta-2-agonists
B) systemic glucocorticoids
C) anticholinergics
D) leukotriene-receptor inhibitor
E) Theophylline

5. Which vaccinations are **especially** important in COPD patients? (COPD pathogens/vaccinations)
A) Tetanus and Diphtheria
B) Measles, Mumps, Rubella
C) Polio and Hepatitis B
D) Influenza and Pneumococcal disease
E) Varicella and Herpes Zoster

6. A 19-year old patient is presenting herself with a cough. She has been coughing for about a month now and taking ACC and Paracetamol has not helped. She has been smoking 10-20 cigarettes/day since she was 14 years old. What diagnostics do you need to differentiate COPD from Asthma? (COPD Dif.Diagnosis)
A) Chest X-Ray
B) Bronchodilator test
C) Blood test
D) Sputum diagnostics
E) Ultrasound

7. Which statement is true about risk factors for COPD? (COPD risk factors)
A) Men are more affected from COPD because of indoor air pollution.
B) In 50% of the times smoking is the cause for COPD.
C) Alpha-2-antitrypsin deficiency can be considered a risk factor.
D) Early childhood overweight can help cause COPD later in life.
E) A history of Tuberculosis is a risk factor for COPD.

8. Your patient has a FEV1 of 60%. Which GOLD group (1-4) would he be in? (GOLD 1-4)
A) 1
B) 2
C) 3
D) 4

9. Which of the following is a radiological sign of lung emphysema? (COPD X-Ray)
A) horizontal rips

B) general hypo transparency of the lung
C) high level of diaphragm
D) narrowing of space between rips
E) ‘crazy paving’-pattern

10. Which of the following is essential in the management of COPD? (COPD management)
A) diagnostics for osteoporosis
B) regular monitoring of thyroid hormone levels
C) routine ultrasound of kidneys
D) monitoring of liver parameters
E) diagnostics of Crohn’s disease

11. You are treating a patient in COPD group B. Which therapy is indicated according to the ABCD-Scheme? (COPD therapy 2)
A) Theophyllin
B) LAMA+ICS
C) LABA+ICS
D) LAMA or LABA
E) LAMA+LABA+ICS

12. Which of the following spirometric findings can be found in COPD patients? (% of reference value) (COPD Spiro 1)
A) PEF 120% (Peak Expiratory Flow)
B) FEV1 50% (Forced Expiratoy Volume in 1 Sec.)
C) FEV1/FVC 93% (Forced Expiratory Volume in 1 Sec./Forced Vital Capacity)
D) RV 65% (Residual Volume)
E) TLC 53% (Total Lung Capacity)

13. Which Flow-Volume curve is typical for asthma? (COPD Spiro 2)

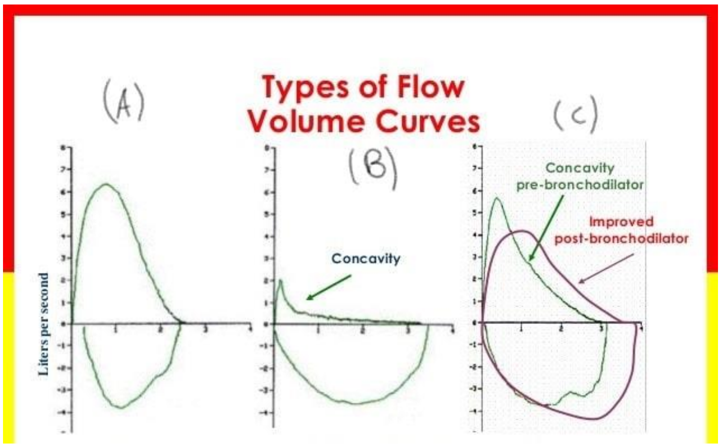

https://copdbloge.blogspot.com/2019/05/copd-spirometry-reversibility.html
A) (a)
B) (b)
C) (c)

14. Mr. Balewa is 75 years old and a long-term COPD patient. He has smoked 20 cigarettes a day for 50 years of his life and now has severe dyspnea, also when he is just getting dressed, chronic cough and fatigue. He scored 11 in the COPD Assessment Test (CAT). Last year, he had one exacerbation. In which group of the ABCD-Scheme is Mr. Balewa? (COPD ABCD)
A) Group A

B) Group B
C) Group C
D) Group D

15. Which statements about the worldwide epidemiology of COPD is correct? (COPD epidemiology)
A) Global prevalence of COPD is 5%.
B) COPD is the 4th leading cause of death in adults.
C) 90% of COPD deaths occur in Low-and Middle-Income countries.
D) Women are more affected than men.
E) Prevalence is predicted to decrease due to efficient prevention programs.
