# Supplementary material for: Real-life Evaluation of an Interactive Versus Noninteractive e-Learning Module on Chronic Obstructive Pulmonary Disease for Medical Licentiate Students in Zambia: Web-Based, Mixed Methods Randomized Controlled Trial
Source: JMIR Med Educ. 2022 Feb 24;8(1):e34751. doi: 10.2196/34751 (PMC8914755; doi:10.2196/34751)
Supplement: Multimedia Appendix 5 [file mededu_v8i1e34751_app5.pdf]

Study information sheet

An evaluation study comparing two e-learning methods for chronic obstructive pulmonary disease in Zambia

Dear participant,

please read or listen to the following explanation carefully and discuss it with the study operator Elena Schnieders (E-MAIL) or the local study operator Misho Mbewe (E-MAIL) or LMMU Prof (hon) Florian Neuhann if you wish to. It tells you important information about a study that we conduct here. COPD (chronic obstructive pulmonary disease) is, as you may know, an increasing burden for health care systems and people in low-and middle-income countries. Studies have shown that knowledge of diagnosis and management of the disease is insufficient in Sub-Saharan Africa. After reading this you may decide if you want to participate in this study or not. Please take your time and ask a member of our research team if you have any questions. This study is organized by the University Hospital Heidelberg in Germany and the School of Medicine and Clinical Sciences, Levy Mwanawasa Medical University, Lusaka, Zambia.

Intention of the study

We would like to evaluate two e-learning methods in self-directed learning in order to optimise competency of future Medical Licentiates (BSc Clin Sciences) health care personal with regard to COPD.

Details of the study

This study will be conducted online. If you are willing to participate, you will be randomly assigned to one of 2 different types of e-learning material about the same topic. Once you have completed the course (about 40 minutes), you will be asked to answer a knowledge test about what you have learned and you will be asked to fill out a small questionnaire about your satisfaction with the course (about 15 minutes). In a few weeks you will be asked to take another, altered knowledge test (about 15 minutes). The date for this will be communicated to you by the study operators. A few study participants will be asked to participate in an evaluation discussion round, in which the sound will be recorded (about 30 minutes). You may choose if you would like to participate or not. Due to Covid-19, the study process might be subject to changes on short notice, but you will be informed if that is the case.

Risks and benefits of the study

There are no risks involved in this study. By participating in this study, you will not be exposed to a higher risk of infection with the Corona virus, as the study is conducted online. You benefit from this study, because you will improve your knowledge about the topic, which is becoming an increasing problem for your future patients. In participating you contribute to the continuous improvement of

the learning – teaching quality at Levy Mwanawasa Medical University and the BSc Clin Sciences program.

Information about privacy, data protection and voluntariness

This study complies with the data protection rules of Zambia, Germany and Europe. During the process of this study, some of your personal information will be collected. Confidentiality and pseudonymity will be maintained, and it will not be possible to identify you in any scientific writing. Pseudonymity means that your name and other personal data will be linked to a study ID and only this study ID will be used for further processing. During the time of the study, personal information and pseudonymization key will be kept safe and only known to members of the research team and will be deleted as soon as possible. Once the pseudonymization key is deleted, your data will be anonymized and cannot be traced back to you. The study operators will do all necessary steps to keep your data safe in accordance with international data protection standards. Pseudonymized/anonymized study data will be saved on the server of Heidelberg University with access for Zambian study team members and will not be transferred from there. You have the right to obtain information of all your personal data, have it changed, or have it deleted at any point if you wish. All study data will be deleted one year after publication.

Your participation in this study is entirely voluntary. If you decide to take part in this study, you are free to withdraw at any time without explaining your reasons. Just contact any study operator. Refusal to participate or withdrawal from the study will not involve loss of any benefits to which you are otherwise entitled. The participation in the evaluation study will not be counted in your overall assessment for your BSc studies.

In case of withdrawal from the study, personal data already obtained can be destroyed upon request. If you agree, your data may continue to be evaluated confidentially. Should you wish to change your decision at a later date, please contact the study operator. Please note that data from previous analyses or data that have already been pseudonymized cannot be deleted.

If you decide to take part, you will be given this information sheet for your records and you will be asked to send a mail to E-MAIL with an informed consent form.

If you do not want to participate anymore or have any questions concerning the study and your data, you may contact the local study operator Misho Mbewe (E-MAIL) or study operator Elena Schnieders (E-MAIL) or Prof (hon) Florian Neuhann, School of Medicine and Clinical Sciences, LMMU (E-MAIL). The main responsible person for all your data is: Elena Schnieders. In case of unlawful data processing, you may contact the following supervisory authorities:

Zambia Information and Communication Technology Authority, which regulates data privacy and protection issues. (Phone: +260211378200, Web: www.zicta.zm)

The representative for data protection and information freedom in Baden- Württemberg, Germany. (E-Mail: poststelle@lfdi.bwl.de, Web: www.baden-wuerttemberg.datenschutz.de)

If you have any other questions concerning your data protection, you may contact the data protection officer of Heidelberg University as well. Just write a mail to: datenschutz@med.uni-heidelberg.de

Will I receive a compensation for participating?

Yes, you will receive an airtime voucher in the end of the study, as you will need to use some of your internet for participating in the study.

Additional information

If you have any other questions about this study and study results, please do not hesitate to contact the study operator Elena Schnieders (Mail: E-MAIL) or the local study operator.

I would be very thankful if you participated in this study!
